# A partial human LCK defect causes a T cell immunodeficiency with intestinal inflammation

**DOI:** 10.1084/jem.20230927

**Published:** 2023-11-14

**Authors:** Victor G. Lui, Manfred Hoenig, Berenice Cabrera-Martinez, Ryan M. Baxter, Josselyn E. Garcia-Perez, Olivia Bailey, Atanu Acharya, Karl Lundquist, Jesusa Capera, Paul Matusewicz, Frederike A. Hartl, Marco D’Abramo, Josephine Alba, Eva-Maria Jacobsen, Doris Niewolik, Myriam Lorenz, Ulrich Pannicke, Ansgar S. Schulz, Klaus-Michael Debatin, Wolfgang W. Schamel, Susana Minguet, James C. Gumbart, Michael L. Dustin, John C. Cambier, Klaus Schwarz, Elena W.Y. Hsieh

**Affiliations:** 1 Department of Immunology and Microbiology, School of Medicine, University of Colorado Anschutz Medical Campus, Aurora, CO, USA; 2 Department of Pediatrics, University Medical Center Ulm, Ulm, Germany; 3 School of Physics, Georgia Institute of Technology, Atlanta, GA, USA; 4 BioInspired Syracuse and Department of Chemistry, Syracuse University, Syracuse, NY, USA; 5 Nuffield Department of Orthopaedics Rheumatology and Musculoskeletal Sciences, The Kennedy Institute of Rheumatology, University of Oxford, Oxford, UK; 6 Faculty of Biology, University of Freiburg, Freiburg, Germany; 7 BIOSS Centre for Biological Signalling Studies and CIBSS Centre for Integrative Biological Signalling Studies, University of Freiburg, Freiburg, Germany; 8 Center of Chronic Immunodeficiency, University Clinics and Medical Faculty, University, Freiburg, Germany; 9 Department of Chemistry, Sapienza University of Rome, Rome, Italy; 10 Department of Biology, Université de Fribourg, Fribourg, Switzerland; 11 Institute for Transfusion Medicine, University of Ulm, Ulm, Germany; 12 Human Immunology and Immunotherapy Initiative, University of Colorado Anschutz School of Medicine, Aurora, CO, USA; 13 Institute for Clinical Transfusion Medicine and Immunogenetics Ulm, German Red Cross Blood Service Baden-Wuerttemberg-Hessen, Ulm, Germany; 14 Department of Pediatrics, Section of Allergy and Immunology, Children’s Hospital Colorado, University of Colorado Anschutz School of Medicine, Aurora, CO, USA

## Abstract

Lymphocyte-specific protein tyrosine kinase (LCK) is essential for T cell antigen receptor (TCR)–mediated signal transduction. Here, we report two siblings homozygous for a novel *LCK* variant (c.1318C>T; P440S) characterized by T cell lymphopenia with skewed memory phenotype, infant-onset recurrent infections, failure to thrive, and protracted diarrhea. The patients’ T cells show residual TCR signal transduction and proliferation following anti-CD3/CD28 and phytohemagglutinin (PHA) stimulation. We demonstrate in mouse models that complete (*Lck*^*−/−*^) versus partial (*Lck*^*P440S/P440S*^) loss-of-function LCK causes disease with differing phenotypes. While both *Lck*^*−/−*^ and *Lck*^*P440S/P440S*^ mice exhibit arrested thymic T cell development and profound T cell lymphopenia, only *Lck*^*P440S/P440S*^ mice show residual T cell proliferation, cytokine production, and intestinal inflammation. Furthermore, the intestinal disease in the *Lck*^*P440S/P440S*^ mice is prevented by CD4^+^ T cell depletion or regulatory T cell transfer. These findings demonstrate that P440S LCK spares sufficient T cell function to allow the maturation of some conventional T cells but not regulatory T cells—leading to intestinal inflammation.

## Teaser

Lui et al. describe a human *LCK* variant causing partial loss-of-function (LOF), including defective TCR signaling and T cell development. Using mouse models, they show that while complete LCK LOF results in SCID, partial LCK LOF causes CID with autoimmunity.

## Graphical Abstract

**Figure:**
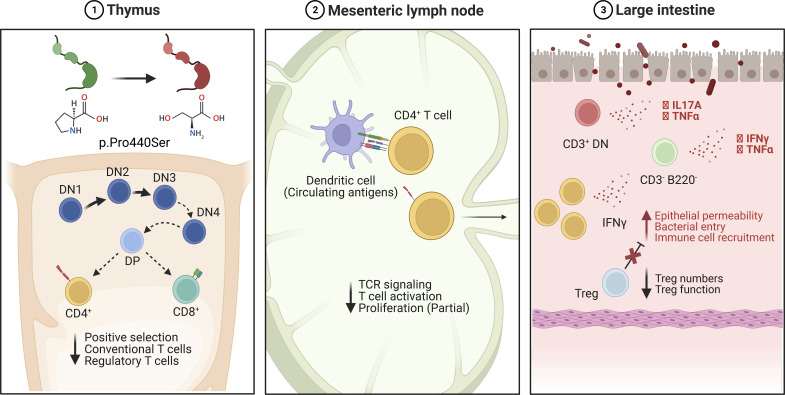

## Introduction

Combined immunodeficiencies (CID) comprise a heterogeneous cluster of disorders characterized by partial reduction (not absence) in T cell number and/or function. Patients with CID suffer from increased susceptibility to various infectious agents and often exhibit autoimmunity and/or inflammation, suggesting that residual T cell numbers/function are causative. Mutations that perturb proximal T cell receptor (TCR) signal strength and thereby result in CID include defects in TCR α constant chain (TCRα) (Morgan et al., 2011), cluster of differentiation of three subunits (CD3γ, CD3δ, CD3ε, CD3ζ) (de Saint Basile et al., 2004, Gil et al., 2011; Dadi et al., 2003; Arnaiz-Villena et al., 1992), CD8 (de la Calle-Martin et al., 2001), RAS homolog family member H (Crequer et al., 2012), lymphocyte-specific protein tyrosine kinase (LCK) (Hauck et al., 2012; Li et al., 2016), linker of activation of T cells (LAT) (Keller et al., 2016; Bacchelli et al., 2017), IL-2–inducible T cell kinase (ITK) (Huck et al., 2009; Serwas et al., 2014), and ζ chain–associated protein kinase 70 (ZAP70) (Chan et al., 1994; Elder et al., 1994). Complete loss-of-function (LOF) variants/mutations of some of these genes can underlie severe CID (SCID), defined by a complete lack of T cell numbers/function. In contrast, less severe degrees of TCR-signal attenuation often permit survival of some T cells, including autoreactive T cells, normally deleted in the thymus, resulting in CID. This spectrum of TCR signaling impairment and association of immunodeficiency and autoimmunity has been shown for humans and mice with defects in ZAP70 and other proteins involved in TCR-dependent signaling (Arpaia et al., 1994; Chan et al., 1994; Roifman et al., 2010; Tanaka et al., 2010; Sakaguchi et al., 2012; Siggs et al., 2007). However, the mechanisms underlying autoimmunity/inflammation initiated by each of these genes in CID patients have not yet been clearly delineated.

LCK is a Src family kinase (SFK) essential for TCR signal transduction and T cell activation (Molina et al., 1992; van Oers et al., 1996a). Its protein structure comprises three Src homology (SH) domains—SH4 (involved in membrane localization), SH3, and SH2. These SH domains are followed structurally by a catalytic kinase domain and a short C-terminal tail (Abraham et al., 1991; Eck et al., 1994). LCK associates with the intracellular domains of CD4 and CD8 (Veillette et al., 1988; Kim et al., 2003). Coaggregation of TCR and CD4 leads to rapid activation of LCK outside lipid rafts, followed by its translocation into lipid rafts and activation of colocalized FYN tyrosine kinase (Filipp et al., 2004). The activity of LCK is itself tightly controlled by conformational changes governed by phosphorylation and dephosphorylation of two regulatory tyrosine residues—one activating phosphotyrosine located in the kinase domain of LCK (Y394) and one inhibitory phosphotyrosine in the C-terminal tail of the protein (Y505) (Abraham and Veillette, 1990; Amrein and Sefton, 1988).

When the C-terminal inhibitory tyrosine Y505 is phosphorylated, it associates with its own SH2 domain, preventing activation (“closed” conformation). Dephosphorylation of Y505 by CD45 results in conformational opening and acquisition of susceptibility to activation by phosphorylation of Y394 (Yamaguchi and Hendrickson, 1996; Ostergaard et al., 1989; Mustelin et al., 1989). The Y505 inhibitory residue is a C-terminal Src kinase (CSK) substrate (Chow et al., 1993; Bergman et al., 1992). CSK access to Y505 is controlled by recruitment to the plasma membrane through CSK-binding protein, which is phosphorylated by LCK to create a docking site for CSK. This is a negative feedback loop where lower global LCK activity is expected to lead to (1) less phosphorylation of Y505 and (2) greater kinase activity per LCK molecule. In addition to Y505, CD45 dephosphorylates other LCK substrates to prevent basal TCR signaling. Inhibition of TCR signaling by CD45 is partly overcome by the exclusion of CD45 from TCR microclusters at the immunological synapse (Varma et al., 2006; Leupin et al., 2000; Davis and van der Merwe, 2006; Kupfer and Singer, 1989; Kupfer et al., 1986; Douglass and Vale, 2005).

LCK mediates T cell activation by initiating signaling pathways that drive mobilization of calcium through activation of phospholipase C-γ (PLCγ) and protein kinase C (PKC) as well as activation of the extracellular signal-regulated kinase (ERK)/mitogen-activated protein kinase (MAPK) pathway (Lovatt et al., 2006). Signaling through LCK is essential for the development of T cell effector potential, particularly for effective cytokine gene transcription. The *Lck*^−/−^ mouse model has revealed the criticality of Lck in TCR signaling, thymocyte ontogeny, and mature T cell activation (van Oers et al., 1996b; Chiang and Hodes, 2016; Molina et al., 1992). However, studies of the impact of partial attenuation of Lck function on thymic T cell development and T cell effector and regulatory functions, and the development of autoimmunity have not been reported.

Mutations affecting LCK protein expression, phosphorylation, regulation, and other structural attributes can be expected to affect its function in TCR signal transduction. Indeed, two human LCK mutations have been found to be associated with T cell immunodeficiency (Hauck et al., 2012; Li et al., 2016). However, how these mutations (1) affect the regulation and activity/function of LCK in TCR signal transduction and (2) cause immunodeficiency and autoimmunity/inflammation remain incompletely understood. We investigated the molecular and cellular effects of a novel *LCK* variant (c.1318C>T; p.P440S) in two siblings presenting with severe T cell lymphopenia, early-onset viral and fungal infections, failure to thrive, and chronic diarrhea. To understand how the P440S LCK variant impacts thymic T cell development, T cell effector and regulatory functions, and the development of autoimmunity/inflammation, we used a knock-in mouse model of the human variant. We hypothesized that complete LOF *LCK* variants (i.e., *Lck*^−/−^) cause a SCID phenotype while partial LOF *LCK* variants, such as the novel P440S variant (*Lck*^*P440S/P440S*^), cause CID with autoimmunity.

## Results

### Clinical presentation, immunological phenotype, and treatment course of siblings with a homozygous P440S LCK variant

**Figure 1.**
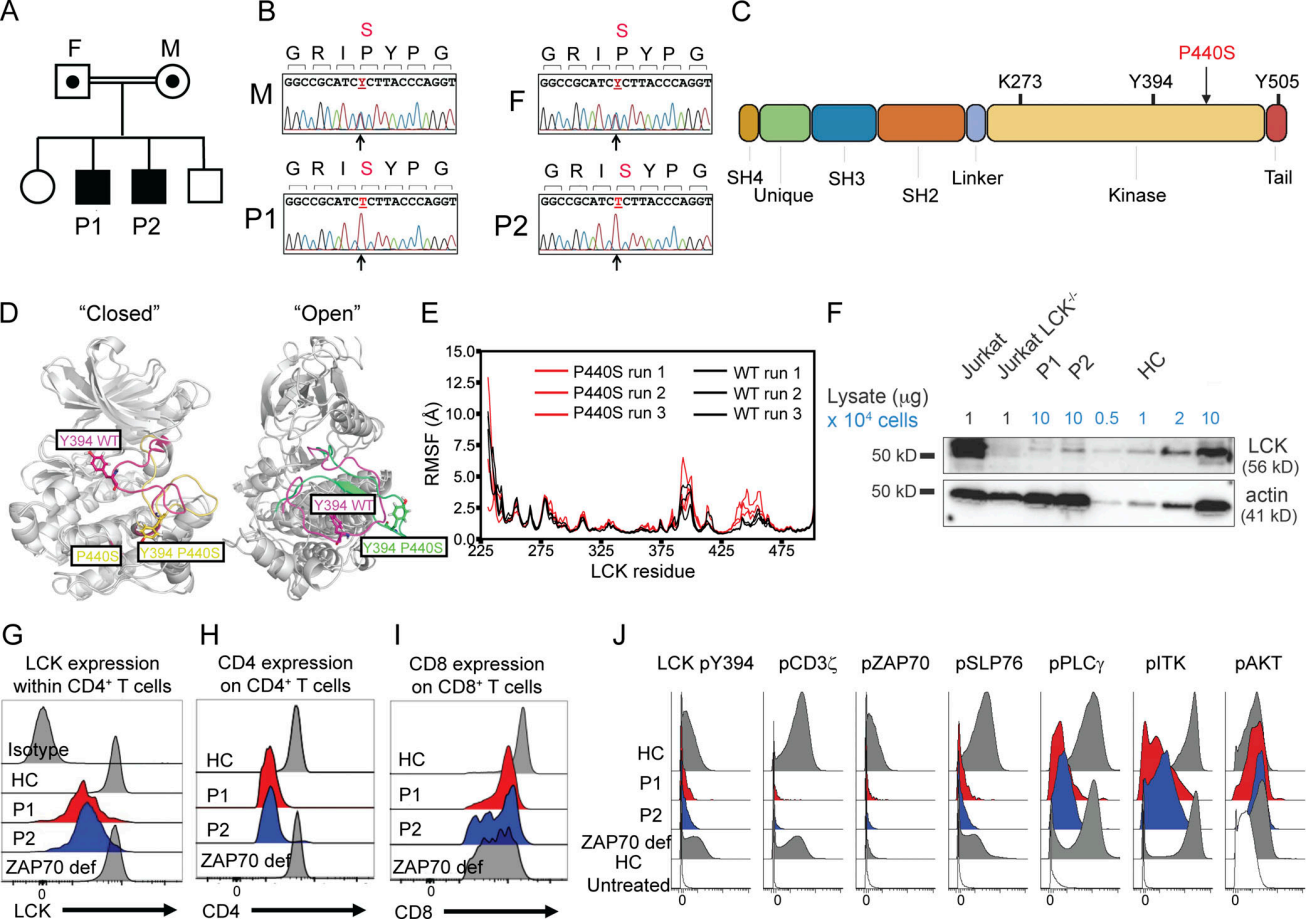
**Identification of novel human LCK P440S variant. (A)** Pedigree of patients’ families carrying the novel homozygous P440S LCK missense mutation. **(B)** Sequencing results of the LCK mutation site within parents and patient siblings. **(C)** Illustration of LCK protein structure. **(D)** Superimposition of the closed and open forms of WT LCK and P440S LCK. Left panel: Superimposition of the two closed forms; the A-loop regions of WT LCK and P440S LCK in magenta and yellow, respectively. Right panel: Superimposition of the two open forms; the A-loop regions of WT LCK and P440S LCK in magenta and green, respectively. **(E)** Root mean square fluctuations of the selected residues of WT LCK (black lines) and P440S LCK (red lines) as provided by molecular dynamics simulations. **(F)** Immunoblot of Jurkat cell lines and sorted CD3^+^ cells from patient PBMCs. **(G)** Intracellular flow staining of LCK total protein within patient CD4^+^ T cells. **(H and I)** Flow cytometry staining of CD4 and CD8 surface expression on patient T cells. **(J)** Mass cytometry measurement of intracellular phosphorylated signaling proteins within CD4^+^ T cells from patient PBMCs treated with pervanadate compared with untreated HC. Source data are available for this figure: SourceData F1.

**Figure S1.**
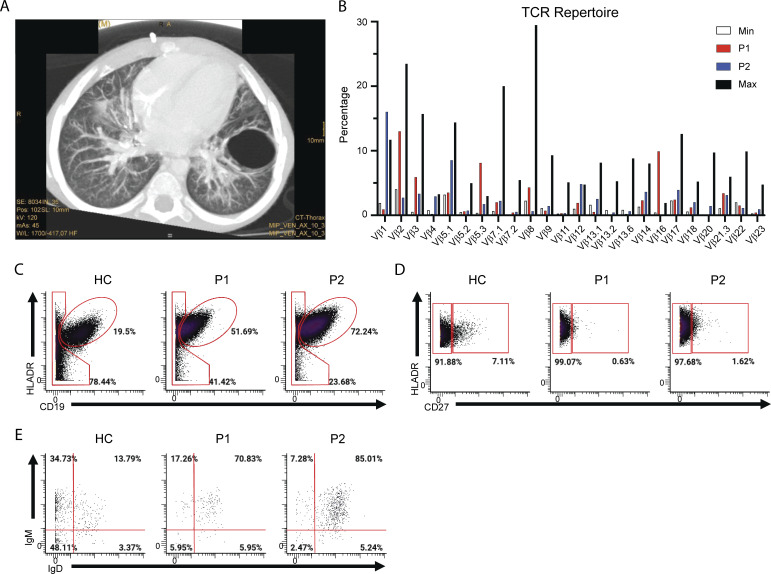
**Patient CT scan and TCR repertoire. (A)** Bronchiectasis and large bulla in patient P1 CT. **(B)** Frequency of Vβ usage in patients P1 and P2. **(C–E)** Frequencies of B cells (CD19^+^HLADR^+^) (C) memory B cells (CD19^+^HLADR^+^CD27^+^) (D), and memory B cell subsets (isotype switched memory [IgM^−^IgD^−^], IgM memory [IgM^+^IgD^−^], pre-switched [IgM^+^IgD^+^], c-delta class switched [IgM^−^IgD^+^]) (E) from mass cytometry immunophenotyping of age-matched HC and patient PBMCs. Percentages of parent gates are shown.

We investigated two brothers born to healthy consanguineous parents from Saudi Arabia (Fig. 1 A). These brothers (P1, P2) presented with infant-onset failure to thrive, recurrent viral and fungal respiratory and gastrointestinal (GI) infections, and chronic diarrhea (Table 1). P1 developed a pulmonary emphysematous bulla (Fig. S1 A) after recurrent respiratory tract infections and, starting at the age of 3 years, esophageal strictures due to recurrent candidiasis. Infection history included HSV stomatitis, oral candidiasis, cryptosporidiosis, and norovirus and salmonella enteritis. Both patients tolerated standard childhood vaccinations, including live vaccines Bacille Calmette-Guerin (BCG), oral polio, and measles, mumps, and rubella. P1 demonstrated protective specific antibody titers to tetanus and *Streptococcus pneumoniae* prior to immunoglobulin replacement (IVIG) at 4 years of age (Table 1). Given P1’s history of infection that suggested an inborn error of immunity (IEI), his younger brother P2 was treated with IVIG beginning at 0.6 years of age.

**Table 1.** Clinical presentation of P440S LCK patients

Laboratory data upon presentation	P1	P2
Age at presentation	First days after birth	1.5 mo
Vaccination	BCG, Hep B at birth, Hep B, polio (oral), DTP, HiB at 2, 4, 6, 18 mo, MMR at 12 mo	BCG, Hep B at birth, Hep B, polio (oral), DTP, HiB at 2, 4, 6 mo
Clinical condition before HSCT	Age: 6.7 years Weight: 17.0 kg (<3rd percentile) Feeding via PEG Coarse “strawy” hair, thrush, distended abdomen, hepato-/splenomegaly Pulmonary CT: Bronchiectasis and emphysematous bulla left lower lobe	Age: 2.8 years Weight: 9.0 kg (<3rd percentile) Hepato-/splenomegaly
Complete blood count (reference ranges)		
Leukocytes (cells/μl)	6,500 (5,200–11,000)	**3,600** (5,200–11,000)
Hemoglobin (g/dl)	**11.2** (11.9–14.7)	**9.0** (10.8–12.8)
Platelet count (cells/μl)	**690,000** (247–436 × 10e3)	**202,000** (286–509 × 10e3)
Neutrophils (cells/μl)	3,130 (2,000–9,000)	**1,510** (2,000–9,000)
Eosinophils (cells/μl)	**0** (80–600)	**0** (80–600)
Basophils (cells/μl)	6 (0-120)	0 (0–120)
Monocytes (cells/μl)	710 (80–720)	430 (80–720)
Lymphocytes (cells/μl)	2,450 (2,300–5,400)	**1,656** (2,300–5,400)
T cell (CD3^+^) phenotype (reference ranges)		
CD3^+^ (cells/μl)	**494** (1,400–3,700)	**298** (1,400–3,700)
CD3^+^CD4^+^ T cells (cells/μl)	**93** (700–2,200)	**232** (700–2,200)
CD3^+^CD8^+^ T cells (cells/μl)	**345** (490–1,300)	**33** (490–1,300)
Naïve T cells (% CD45RA^+^/CCR7^+^ of CD3^+^CD4^+^ cells/μl)	**1** (45–83)	**3** (45–83)
TCRαβ^+^/TCRγδ^+^ T cells (cells/μl)	**445** (1,320–3,780)/**49** (90–500)	**248** (1,320–3,780)/**33** (90–500)
TCRαβ^+^/TCRγδ^+^ T cells (% of CD3^+^)	89/10	86/11
TRECs (number of copies)	**0** (>8)	**0** (>8)
NK-like T cells (cells/μl) CD3^+^CD56^+^CD16^+^	25 (2–80)	**0** (2–80)
T cell (CD3^+^) function (% of control)		
(SI, [% of control]) PHA CD3/CD28 MLC Tetanus Candidin	111 (40) 101 (52) 10 (10) 1 (0) 6 (0)	349 (34) 374 (54) 23 (11) 1 (0) 33 (41)
B cell phenotype (reference ranges)		
CD19^+^ (cells/μl)	963 (390–1,400)	1291 (390–1,400)
Naïve B cells (% CD19^+^IgD^+^CD27^−^ of CD19^+^ cells/μl)	99 (47.3–77.0)	99 (54.0–88.4)
Transitional B cells (% CD19^+^IgM^++^CD38^++^ of CD19^+^)	50 (4.6–8.3)	12 (3.1–12.3)
Memory B cells (% CD19^+^CD27^+^ of CD19^+^ cells/μl)	0.5 (18.6–46.7)	0.7 (7.8–37.1)
Switched memory B cells (% CD19^+^CD27^+^IgM^−^ of CD19^+^ cells/μl)	0 (10.9–30.4)	0.1 (4.7–21.2)
Marginal zone–like B cells (% CD19^+^IgM^+^IgD^+^CD27^+^ of CD19^+^ cells/μl)	0.4 (5.2–20.4)	0.6 (2.7–19.8)
KRECs (number of copies)	209 (>4)	260 (>4)
Serum immunoglobulins		
IgG serum level (g/liter)	8.5^a^ (2.9^b^) (5–14.6)	7.5^a^ (4.5–9.2)
IgM serum level (g/liter)	0.26 (0.24–2.10)	1.74 (0.19–1.46)
IgA serum level (g/liter)	0.08 (0.27–1.95)	0.76 (0.2–1.0)
Specific antibodies^b^	Tetanus, *S. pneumoniae*	n.d

Age-matched reference values for B cell subpopulations taken from Piątosa et al. (2010); age-matched reference values for other blood cell counts taken from Shearer et al. (2003) or certified internal control series. Reference values are age matched. Abnormal values are in bold. Hep B: hepatitis B; DTP: diphtheria, tetanus, pertussis; HiB: hemophilus influenzae type B; MMR: measles, mumps, and rubella; PEG: percutaneous endoscopic gastrostomy; MLC: mixed lymphocyte culture.

a IgG serum level after start of substitution.

b Reported IgG serum level/specific antibodies before the start of substitution.

Immunophenotyping of patients P1 and P2 at ages 6 and 2.7 years old, respectively, demonstrated severe T cell lymphopenia with a significantly decreased naïve compartment, undetectable TCR excision circles, and slightly increased frequency of TCRγδ^+^ cells (Table 1). The CD4/CD8 ratio was inverted in P1 but increased for P2 (Table 1). The TCR repertoire showed some prominent Vβ-rearrangements but was not oligoclonal (Fig. S1 B). Compared with healthy controls (HC), both siblings’ T cells demonstrated decreased thymidine uptake in response to the T cell mitogen phytohemagglutinin (PHA) (P1: 40% of control; P2: 34% of control), TCR/CD28 costimulation (anti-CD3/CD28; P1: 52% of control; P2: 54% of control), and no response to tetanus and candida antigens (Table 1). While total B cell numbers were within the normal range, isotype-switched memory B cells were significantly decreased, likely secondary to the significant T cell lymphopenia (Table 1). Natural killer (NK) and NKT cells were significantly reduced in P2 but within the normal range for P1 (Table 1).

Given the suspicion of IEI, a hematopoietic stem cell transplant (HSCT) was undertaken in P1 and P2 at the age of 6.3 and 3 years, respectively, using the mother as the haploidentical donor. HSCT induction regimen details are found in Table S1. Both patients developed mixed donor chimerism of non-T cells soon after HSCT (Table S2). P1, unfortunately, died on day +102 after transplantation of pneumonia. P2 was treated for BCG-itis with unilateral axillary lymphadenitis on day +135 and discharged 7 mo after HSCT (Table S1). Both patients received CD34-positive selected stem cell boosts from the same donor (mother, as per primary HSCT) due to poor engraftment (Tables S1 and S2).

3 mo after HSCT, P2 was analyzed by trio whole-exome sequencing (Fig. 1 B and Table S3) which revealed a homozygous missense variant in exon 12 of the *LCK* gene (NM_005356.5: c.1318C>T; NP_001036236.1: p.P440S). Residue P440 is located in the catalytic domain of LCK (Fig. 1 C) and is evolutionarily conserved among LCK of vertebrates (data not shown). This variant was predicted to be pathogenic due to several pathogenicity scores including a combined annotation dependent depletion (CADD) score of 26.1 (Table S4). The parents are healthy first cousins, and both are carriers of the *LCK* missense variant (Fig. 1, A and B). The two unaffected siblings were not available for analysis.

### The P440S mutation causes LCK protein instability and defective TCR signal transduction

In silico 3D protein modeling of P440S LCK suggested distortion of the activating loops, likely resulting in (1) significant instability of the region surrounding the P440S variant and (2) altered interaction of Y394 with the neighboring amino acids in the kinase pseudo substrate domain (Fig. 1 D). Indeed, the greater root mean square fluctuation of the primary sequence surrounding P440S predicts higher mobility of this region compared with wild type (WT) LCK (Fig. 1 E). One of these three simulations showed instability of the Y394 activation loop for P440S LCK. To validate this in silico prediction, LCK protein levels in peripheral blood mononuclear cells (PBMC) were evaluated by immunoblotting and flow cytometry. These experiments revealed a ∼10-fold reduction in LCK protein levels in T cells of both patients when compared with an age- and sex-matched HC (Fig. 1, F and G). CD4 surface expression was also decreased in CD4^+^ T cells from P1 and P2 compared to HC and a ZAP70-deficient patient (ZAP70 p.A570T, ZAP70 def) (Fig. 1 H). CD8 surface expression on P1, P2, and ZAP70 def CD8^+^ T cells were all decreased relative to WT (Fig. 1 I). Notably, the downregulation of CD4 in P1 and P2 was more pronounced than CD8. These observations support the uniqueness of the LCK and ZAP70 defects. Decreased CD4 surface expression in P1 and P2 is consistent with the role of LCK in stabilizing CD4 coreceptor retention on the cell surface (Horkova et al., 2023; Pelchen-Matthews et al., 1992). Selective impairment of CD8^+^ T cell development and survival is characteristic in patients with ZAP70 defects (Arpaia et al., 1994; Ashouri et al., 2022; Schim van der Loeff et al., 2014).

To assess TCR signaling in the patients’ T cells, we performed mass cytometry (Table S5) on their PBMC treated with pervanadate and measured the phosphorylation of protein targets involved in TCR signal transduction. Both patients demonstrated decreased phosphorylation of LCK Y394, CD3ζ, ZAP70, and SH2 domain–containing leukocyte protein of 76 kD (SLP76). However, some preservation of phosphorylation of more distal signaling proteins including PLCγ, ITK, and protein kinase B (AKT) serine/threonine kinase phosphorylation was observed (Fig. 1 J). Pervanadate inhibits CD45 and other tyrosine phosphatases, allowing spontaneous accumulation of phosphorylated substrates and assembly of signalosomes. In a synapse, close cellular contact excludes CD45 and acts locally like pervanadate (Davis and van der Merwe, 2006). Hence, the use of pervanadate here serves as a surrogate for TCR stimulation to induce maximal phosphorylation. When PBMC were treated with pervanadate, we expected to see phosphorylation of the proteins downstream of TCR signal transduction that are seen when TCR signaling cascades are intact, as seen in HC T cells treated with pervanadate. However, while in the ZAP70-deficient patient, LCK Y394 phosphorylation was intact, phosphorylation of other proximal TCR-signaling intermediaries such as CD3ζ, ZAP70, and SLP76 was abrogated (Fig. 1 J).

In P1 and P2, downstream phosphorylation of signaling proteins PLCγ1, ITK, and AKT was less affected than the proximal signaling responses. Proximal defects may exist but may be selectively obscured by signal amplification. P1 and P2 demonstrated a similar signaling impairment as the ZAP70-deficient patient, presumably due to the requirement for LCK to phosphorylate ZAP70 during TCR signal transduction (Fig. 1 J). The lack of proximal phosphorylation induced by pervanadate suggests that early TCR signaling is impaired. However, whether this TCR signaling defect is due to decreased T cell LCK expression, regulation, or function cannot be addressed in primary human PBMC.

As expected, B cell antigen receptor downstream signal transduction was unaffected by P440S LCK (data not shown). However, while the numbers of total B cells remained similar to age-matched HC, isotype-switched CD27^hi^ memory B cells were decreased, likely secondary to lack of CD4^+^ T cell helper function (Table 1 and Fig. S1, C–E).

### P440S LCK cell lines exhibit defective TCR signal transduction even when P440S LCK protein levels are normalized to WT LCK

**Figure S2.**
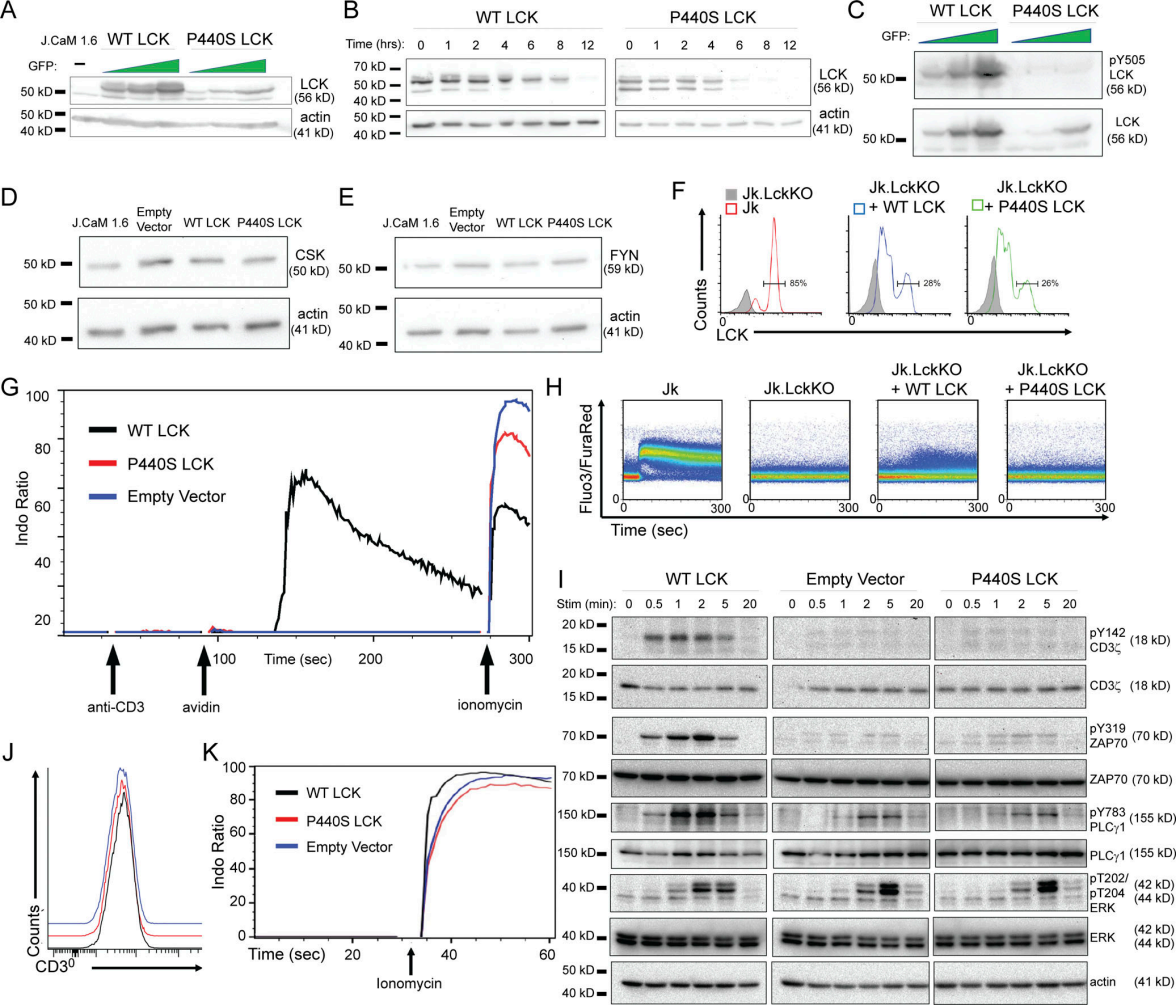
**P440S LCK cell line supplemental data. (A)** Immunoblot of GFP-sorted transduced J.CaM 1.6 cell lines expressing WT or P440S LCK protein. **(B–E)** Immunoblots of cycloheximide protein stability assay (B), LCK pY505 (C), CSK (D), and FYN (E) on transduced J.CaM 1.6 cell lines. **(F)** LCK expression in inducible Jurkat cell line expression system upon treatment with doxycycline. **(G and H)** Calcium mobilization of transduced J.CaM 1.6 cell lines (G) and inducible Jurkat cell line expression system (H) stimulated with anti-CD3. **(I)** Phospho-specific immunoblots of TCR signaling intermediates at indicated stimulation time points. **(J and K)** CD3 surface expression (J) and ionomycin-induced calcium response (K) of transduced J.CaM 1.6 cell lines. Source data are available for this figure: SourceData FS2.

**Figure 2.**
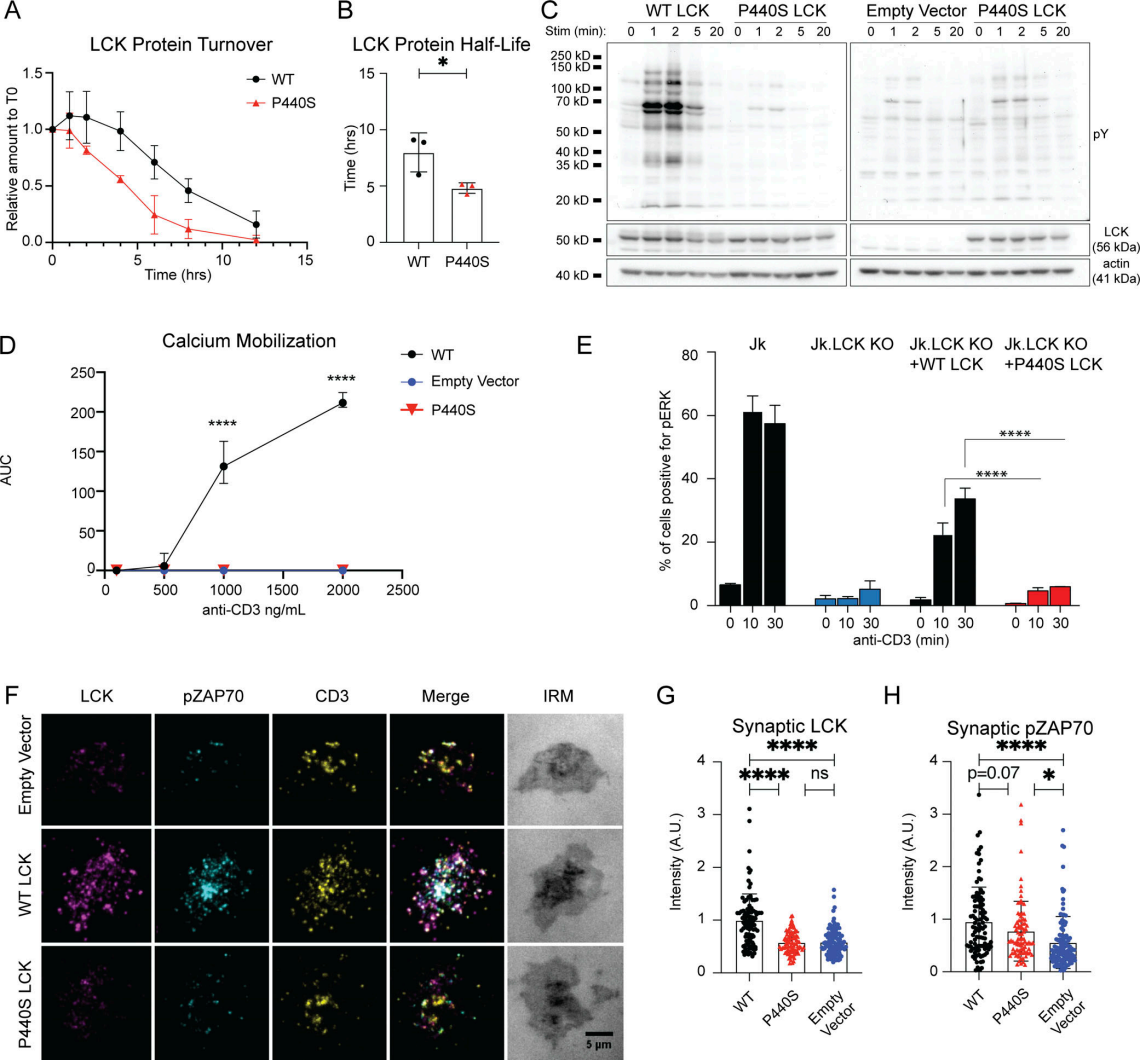
**P440S mutation causes protein instability, decreased protein expression, and defective TCR signaling. (A and B)** Protein stability assay (A) and protein half-life (B) of WT and P440S LCK. **(C and D)** Immunoblot of TCR-mediated global tyrosine phosphorylation (C) and titration curve of TCR-mediated calcium responses (D) of transduced J.CaM 1.6 cell lines. **(E)** Measurement of TCR-mediated pERK activation in inducible Jurkat cell line expression system. **(F–H)** Transduced J.CaM 1.6 lines stimulated on SLB visualized by TIRF (F) and measurement of resultant synaptic LCK recruitment (G) and synaptic ZAP70 phosphorylation (H). Data in A–H are representative of results from at least two independent experiments. Experiments in A, B, D, and E have three samples per group. Data points in F–H are measurements of single cells with at least 90 samples per group. Error bars represent mean and SEM. *P < 0.05, ****P < 0.0001. Not significant unless stated by an asterisk in the figure. Unpaired *t* test was used to test for statistical significance in B and D. Ordinary one-way ANOVA with Tukey’s multiple comparisons test was used to test for statistical significance in E, G, and H. Source data are available for this figure: SourceData F2.

To understand whether the observed TCR signal transduction defect in P1 and P2 was due to the reduced LCK protein expression or function (or both), we transduced the LCK-deficient J.CaM 1.6 cell line using a bicistronic lentiviral vector encoding LCK and a green fluorescent protein (GFP) reporter. Transfectants were produced that express either WT or P440S LCK. LCK protein levels in the P440S cell line were significantly lower than in the WT cell line, even when sorted based on equivalent GFP expression, corroborating the observations in the human PBMCs (Fig. S2 A). To test the possibility that P440S LCK has reduced protein stability, we examined steady-state LCK protein stability by inhibiting protein translation with cycloheximide and measured LCK protein levels over time. This experiment revealed that P440S LCK protein half-life is shorter than WT LCK (Fig. 2, A and B; and Fig. S2 B), and is therefore consistent with findings from the in silico 3D protein stability analysis (Fig. 1, D and E). Additionally, immunoblotting of the transduced J.CaM 1.6 cell line lysates demonstrated decreased basal levels of pY505 on P440S LCK compared with WT LCK (Fig. S2 C). Importantly, WT and P440S cell lines expressed equivalent levels of CSK and FYN (Fig. S2, D and E).

**Figure S3.**
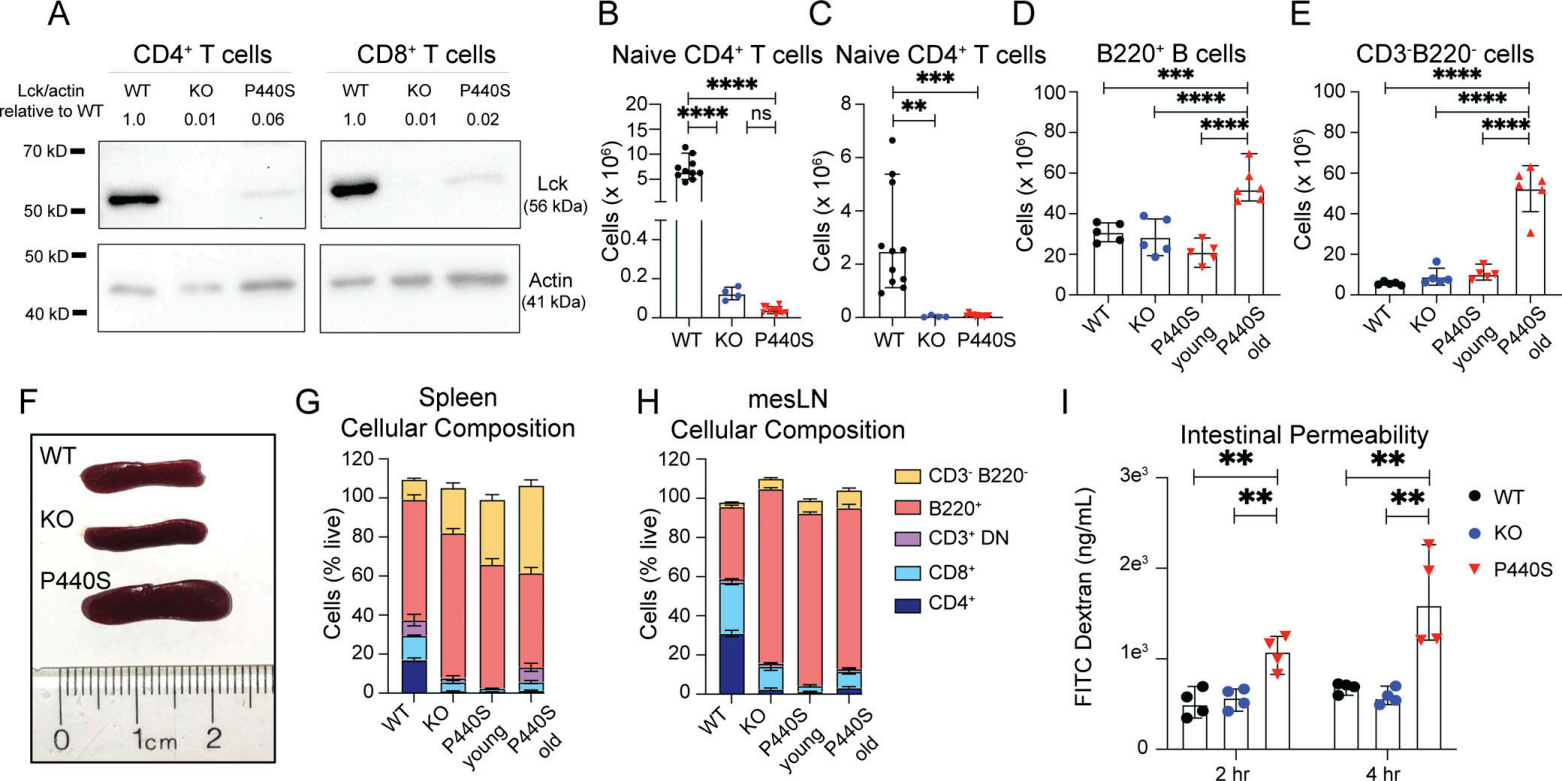
**P440S Lck mouse phenotype supplemental data. (A)** Lck immunoblot on FACS-sorted splenic CD4^+^ and CD8^+^ T cells from mice of the indicated genotypes. **(B and C)** CD62L^hi^CD44^lo^ naïve CD4^+^ T cell counts from spleen (B) and mesLN (C). **(D and E)** Splenic total counts of B cells (B220^+^) (D) and non-T/B cells (CD3^−^B220^−^) (E) from mice of the indicated genotypes. **(F)** Image of whole spleens from mice. **(G and H)** Cellular composition of spleens (G) and mesLN (H) via flow cytometry. **(I)** FITC-dextran intestinal permeability assay performed on mice. Young P440S mice were 5–6 wk of age. All other mice were 20 wk of age. Experiment in A is representative of results from two independent experiments from pooled mice. Experiments in B and C are representative of results from three independent experiments with 4–11 mice per group. Experiments in D–I are representative of two independent experiments with four to six mice per group. Error bars represent median and 95% CI. **P < 0.01, ***P < 0.001, ****P < 0.0001. Not significant unless stated by asterisk in figure. Ordinary one-way ANOVA with Tukey’s multiple comparisons test was used to test for statistical significance for all experiments. Source data are available for this figure: SourceData FS3.

To investigate the effects of P440S LCK on TCR signaling (independent of the LCK protein expression defect), we sorted the transduced J.CaM 1.6 cell lines based on GFP expression and confirmed comparable WT and P440S LCK protein expression by immunoblot (Fig. S2 A). To circumvent compensation effects that may arise from the constitutive LCK expression in the transduced J.CaM 1.6 cell line, we transfected Jurkat (Jk) cells lacking expression of endogenous LCK (Jk.LckKO) with doxycycline-inducible WT (Jk.LckKO + WT LCK) or P440S LCK (Jk.LckKO + P440S LCK). Upon doxycycline treatment, ∼30% of the transduced cells expressed LCK (Fig. S2 F), normalizing LCK expression levels across the induced cell lines. Both the transduced J.CaM 1.6 and inducible Jk.LckKO cell lines were stimulated by crosslinking TCR with soluble anti-CD3. In these P440S LCK cell line systems, we observed nearly absent TCR-mediated (1) global tyrosine phosphorylation (Fig. 2 C), (2) calcium mobilization (Fig. 2 D and Fig. S2, G and H), and (3) phosphorylation of ERK (pERK) (Fig. 2 E). Phosphorylation of CD3ζ Y142, and ZAP70 Y319 in the empty vector and P440S LCK transduced J.CaM 1.6 cell lines was impaired (Fig. S2 I). We also observed delayed and diminished PLCζ Y783 phosphorylation, consistent with the inability to mobilize a TCR-mediated calcium. Surface expression of CD3 and ionomycin-induced calcium responses were comparable within the transduced J.CaM 1.6 cell lines (Fig. S2, J and K). These findings demonstrate an intrinsic functional defect, in addition to the expression defect caused by protein instability, regardless of whether P440S LCK is introduced in a constitutively expressed or inducible system.

Stimulation experiments using soluble anti-CD3 do not benefit from CD45 exclusion from TCR microclusters. To determine if P440S LCK can generate a signaling response under conditions that simulate synapse formation, we stimulated the transduced J.CaM 1.6 cell lines using supported lipid bilayers (SLBs) presenting laterally mobile anti-CD3 along with adhesion/costimulatory molecules intercellular adhesion molecule 1 (ICAM-1, or CD54), CD58, and CD80. We then examined early TCR signaling events at the synaptic interface using total internal reflection fluorescence (TIRF) microscopy, a subcellular assessment of TCR signalosome output that is quantified at a single cell level (Fig. 2 F). In the P440S LCK J.CaM 1.6 cell line, the TCR-mediated recruitment of P440S LCK was below the detection limit established by the comparison to the response of the empty vector negative control and WT LCK positive control cell lines (Fig. 2 G). However, P440S LCK J.CaM 1.6 generated an intermediate synaptic ZAP70 phosphorylation response between the empty vector negative control and WT LCK positive control cell lines (Fig. 2 H). These findings, while they are in a constructed system with supraphysiologic stimuli, suggest that P440S LCK fail to recruit to TCR microclusters but can partially phosphorylate ZAP70 at synapses.

### The *Lck*^*P440S/P440S*^ knock-in mouse recapitulates the patients’ clinical and immunological phenotype while the *Lck*^*−/−*^ mouse does not

The findings from the study of primary human cells and cell lines demonstrated that the P440S mutation leads to significantly decreased LCK protein levels and impaired TCR signal transduction. To investigate the in vivo impact of P440S LCK, we compared the *Lck*^*−/−*^ (KO) mouse to a newly generated CRISPR/Cas9 knock-in mouse bearing the murine homolog of the human LCK P440S variant (*Lck*^*P440S/P440S*^, P440S). Founder animals were screened for off-target effects and propagated. Like the carrier parents, heterozygous P440S mice did not exhibit detectable phenotypic or immunological abnormalities (data not shown).

**Figure 3.**
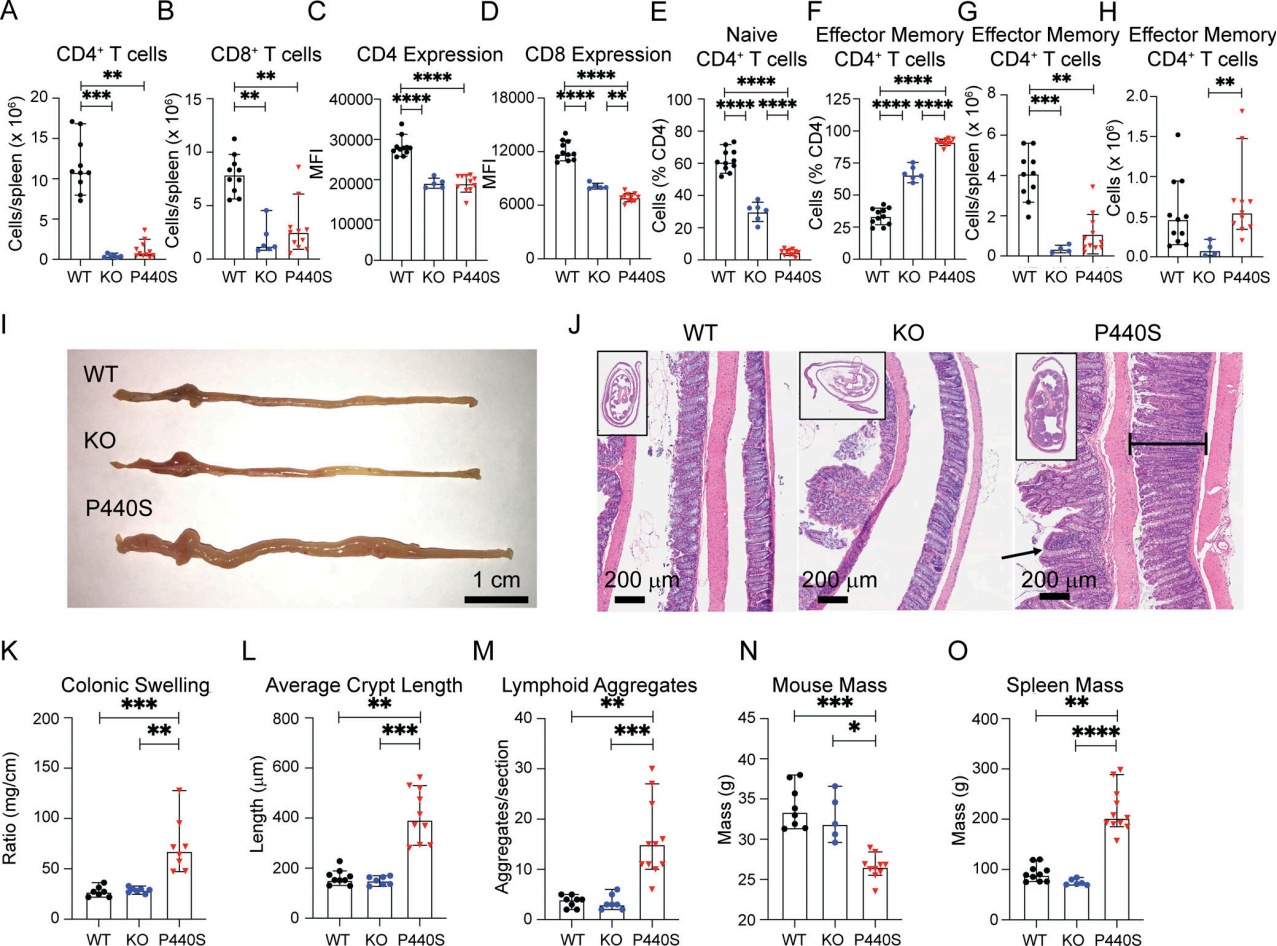
**P440S mice phenocopy patients’ T cell phenotype and intestinal inflammation. (A and B)** Total numbers of splenic CD4^+^ (A) and CD8^+^ (B) T cells from indicated mice. **(C and D)** Surface expression of CD4 (C) and CD8 (D) on splenic T cells. **(E and F)** Frequencies of CD62L^hi^CD44^lo^ naïve (E) and CD62L^lo^CD44^hi^ memory (F) splenic CD4^+^ T cell subsets. **(G and H)** Total numbers of CD62L^lo^CD44^hi^ memory CD4^+^ T cells from spleen (G) and mesLN (H). **(I and J)** Gross (I) and H&E histological (J) images of large intestines from mice of the indicated genotype. **(K)** Ratios of colon length and mass (K). **(L and M)** Average crypt length (L) and number of lymphoid aggregates per histological section (M) from H&E histology. **(N and O)** Masses of mice (N) and whole spleens (O). Length of the scale bar in I is 1 cm. Length of bars in J is 200 μm. All measurements were taken from mice at 20 wk of age. Experiments in A–O are representative of results from three independent experiments with 5–11 mice per group. Error bars represent the median and 95% CI. *P < 0.05, **P < 0.01, ***P < 0.001, ****P < 0.0001. Not significant unless stated by an asterisk in the figure. Kruskal–Wallis accounting for multiple comparisons was used to test for statistical significance for all experiments.

The homozygous *Lck*^*P440S/P440S*^ mice showed a heritable immunological and symptomatic phenotype similar to that of the patients, with no significant differences observed between males and females. These shared characteristics between the P440S mice and the patients include (1) a 20- and 50-fold reduction in Lck protein expression by splenic CD4^+^ and CD8^+^ T cells, respectively (Fig. S3 A); (2) severe (splenic) T cell lymphopenia (Fig. 3, A and B); (3) decreased CD4 and CD8 expression by T cells (Fig. 3, C and D); and (4) skewed CD4^+^ T cell memory phenotype (Fig. 3, E and F). Naïve (CD44^lo^CD62L^hi^) CD4^+^ T cell numbers were reduced in the spleens and mesenteric lymph nodes (mesLN) of KO and P440S mice compared with WT (Fig. S3, B and C). However, compared with WT mice, P440S mice showed decreased numbers of effector memory (Tem, CD44^hi^CD62L^lo^) CD4^+^ T cells in the spleen but similar numbers in mesLN (Fig. 3, G and H). These findings demonstrate the accumulation of the Tem compartment in the P440S mice in the intestinal draining LN only, suggesting a local T cell–driven process. Additionally, both young P440S (aged 6 wk) and KO mice showed similar B cell numbers (Fig. S3 D). These findings demonstrate that the *Lck*^*P440S/P440S*^ mouse model phenocopies the P440S LCK patients. Hence, its comparison with the *Lck*^*−/−*^ mouse model serves to define the difference between the impact of a Lck complete defect (KO) and a partial defect (P440S) on TCR signal transduction, T cell selection, T cell development, and T cell differentiation processes.

**Figure 4.**
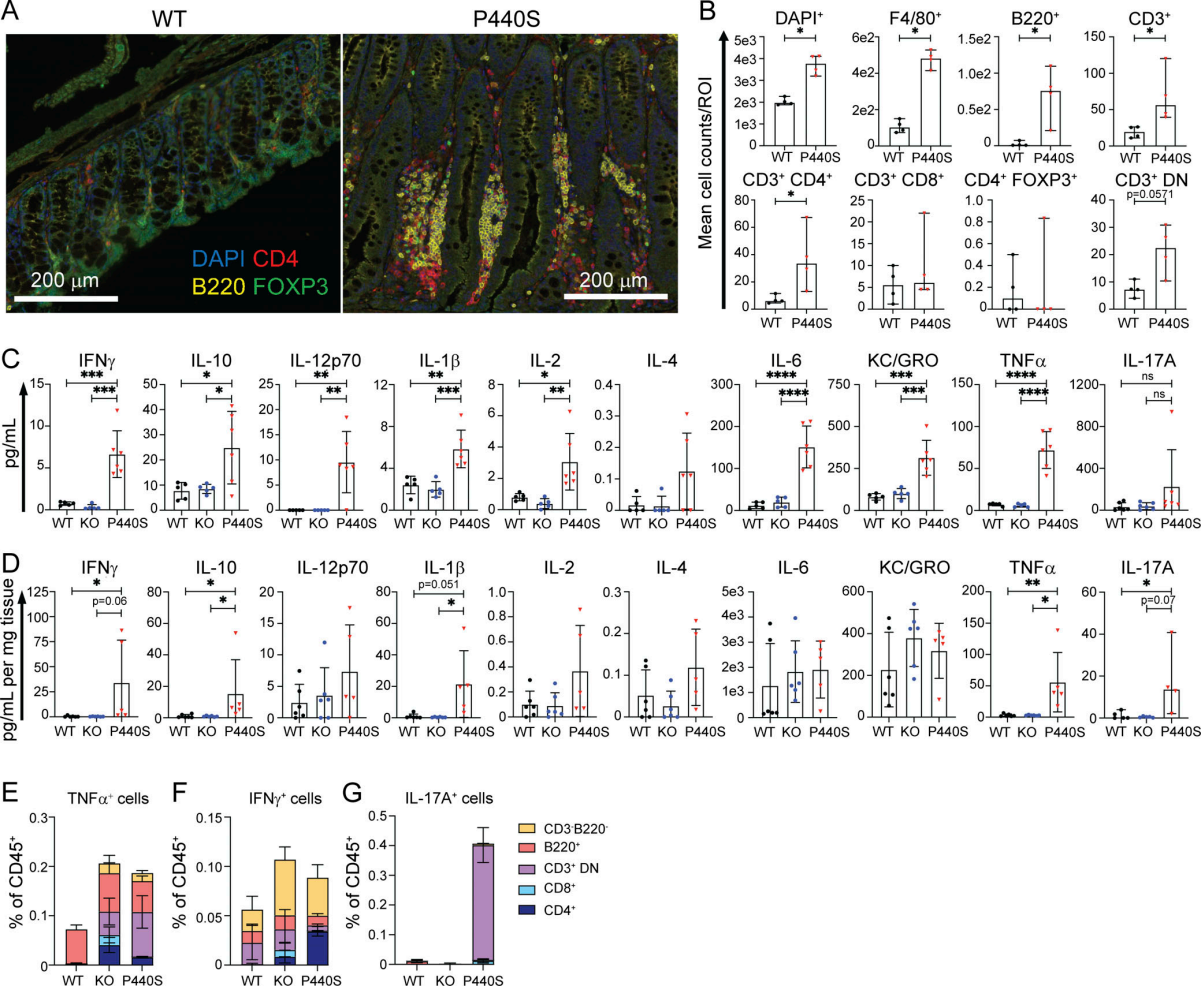
**P440S mice intestinal inflammation demonstrates increased T/B lymphoid aggregates and Th-17 skewing. (A and B)** Multispectral imaging of large intestines (A) and mean counts of indicated cell type per region of interest (ROI) (B) from WT and P440S mice. **(C and D)** Cytokine concentration from serum (C) and colon culture supernatants (D). **(E–G)** Intracellular cytokine staining of dissociated lamina propria cells. All measurements were taken from mice at 20-wk of age. Experiments in A–G are representative of results from two independent experiments with four to six mice per group. The length of bars in A is 200 μm. Error bars represent the median and 95% CI. *P < 0.05, **P < 0.01, ***P < 0.001, ****P < 0.0001. Not significant unless stated by an asterisk in figure. Kruskal–Wallis accounting for multiple comparisons was used to test for statistical significance for all experiments.

**Figure 5.**
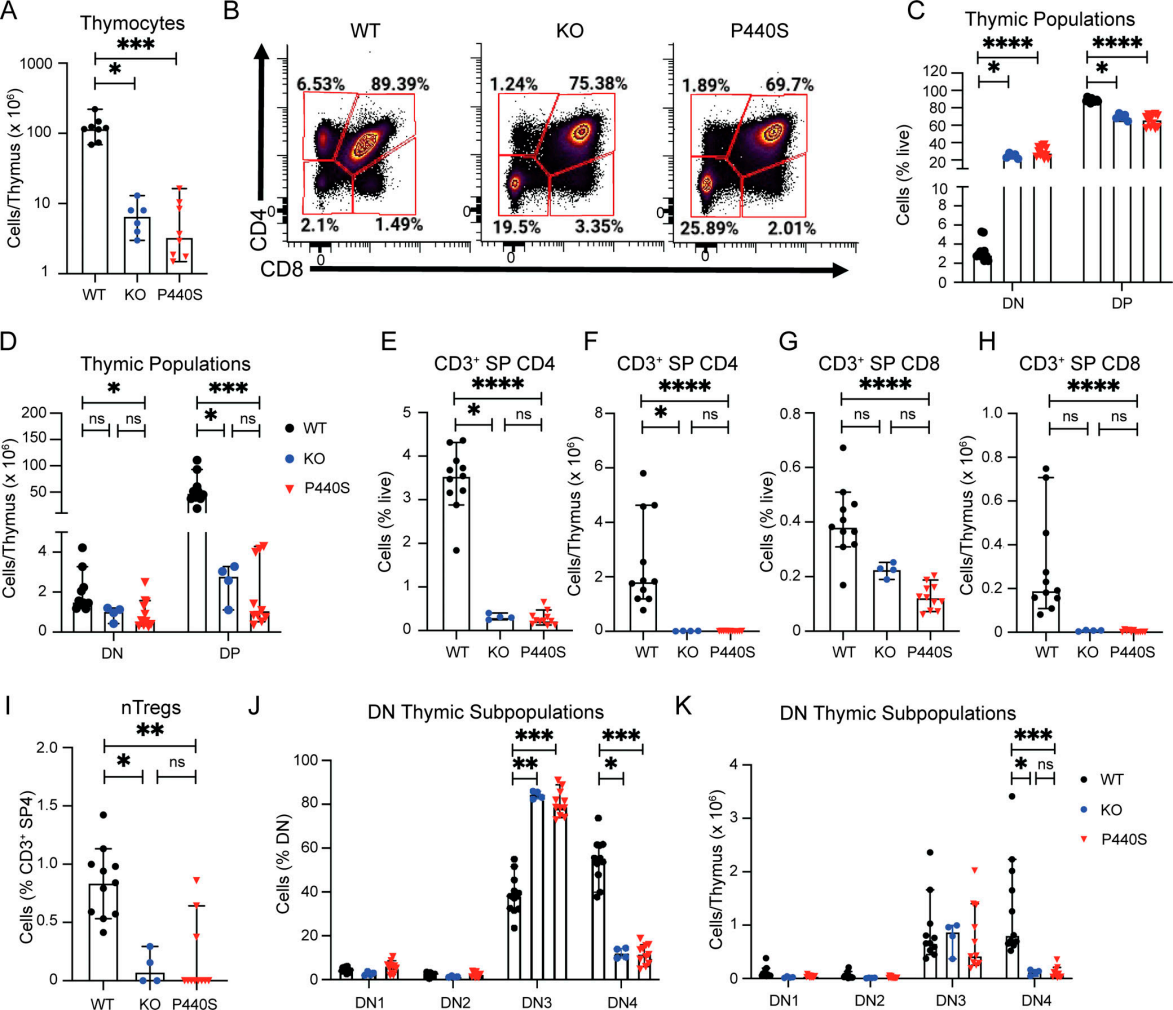
**P440S and KO mice have defective T cell thymic development. (A)** Total thymocyte cell counts from mice of the indicated genotypes. **(B)** Flow staining of thymic cellular subsets, gated on total live thymocytes. **(C–H)** Frequencies and cell counts for subsets of thymic T cell precursors (CD4^−^CD8^−^ [DN], CD4^+^CD8^+^ [DP], CD3^+^CD4^+^CD8^−^ [mature SP4], CD3^+^CD4^−^CD8^+^ [mature SP8]). **(I)** Frequency of thymic-derived Tregs (mature SP4 CD25^+^FOXP3^+^, nTregs). **(J and K)** Frequency and counts of thymic DN subpopulations (CD25^−^CD44^+^ [DN1], CD25^+^CD44^+^ [DN2], CD25^+^CD44^−^ [DN3], CD25^−^CD44^−^ [DN4]). Experiments in A–K are representative of results from three independent experiments with 4–11 mice per group. Error bars represent the median and 95% CI. *P < 0.05, **P < 0.01, ***P < 0.001, ****P < 0.0001. Not significant unless stated by an asterisk in the figure. Kruskal–Wallis accounting for multiple comparisons was used for statistical significance testing for all experiments.

In contrast to KO mice, P440S mice developed intestinal inflammation (Fig. 3, I–M), weight loss (Fig. 3 N), and splenomegaly (Fig. 3 O and Fig. S3, E–G), suggesting that residual P440S LCK protein enables the GI pathology in P440S mice. The splenomegaly in the P440S mouse was composed primarily of B220^+^ B cells and CD3^−^B220^−^ non-T/B cells, similar to the mesLN, which drain the intestines (Fig. S3, G and H). At ∼20 wk of age, only the P440S mice demonstrated gross and histological evidence of intestinal inflammation. These mice displayed increased (1) lymphocytic infiltrate (Fig. 3 J), (2) colonic swelling (Fig. 3 K), (3) crypt length (Fig. 3 L), (4) number of lymphoid aggregates per histological section (Fig. 3 M), and (5) intestinal permeability (Fig. S3 I). Multispectral imaging of intestinal tissue from P440S mice showed (1) increased cellularity, (2) profuse macrophage (F480^+^), CD4^+^ T cell, CD3^+^ double negative (DN) T cell (CD3^+^CD4^−^CD8^−^), and B cell (B220^+^) infiltration, and (3) similar regulatory T cell (Treg) numbers (CD3^+^CD4^+^FOXP3^+^) (Fig. 4, A and B).

Additionally, assessment of serum and supernatant from intestinal tissue cultures revealed elevation of IFNγ, IL-10, IL-12, L-1β, IL-2, IL-6, keratinocyte chemoattractant/human growth-regulated oncogene (KC/GRO), TNFα, and IL-17A in the P440S mice only, supporting the likelihood of systemic and local inflammatory processes (Fig. 4, C and D). Interestingly, IL-6 and KC/GRO were only elevated in the serum, suggesting a more innate proinflammatory picture in the periphery. Intracellular cytokine staining of lamina propria from P440S mice revealed TNFα production in CD3^+^ DN T cells and B220^+^ B cells (Fig. 4 E), while IFNγ was largely produced by the non-T/B compartment (CD3^−^B220^−^) and CD4^+^ T cells (Fig. 4 F). Further supporting the local nature of T cell–driven inflammatory process in the P440S mouse, our results demonstrated increased CD45^+^ lymphocytes that are (1) IFNγ-producing CD4^+^ T cells and (2) IL-17A–producing CD3^+^ DN T cells (Fig. 4, F and G). These data demonstrate that the P440S Lck mutation causes partial LOF and that residual T cell activity underlies the local intestinal inflammation.

### The KO and P440S mice demonstrate similar thymic T cell defects but distinct peripheral T cell functional properties

**Figure S4.**
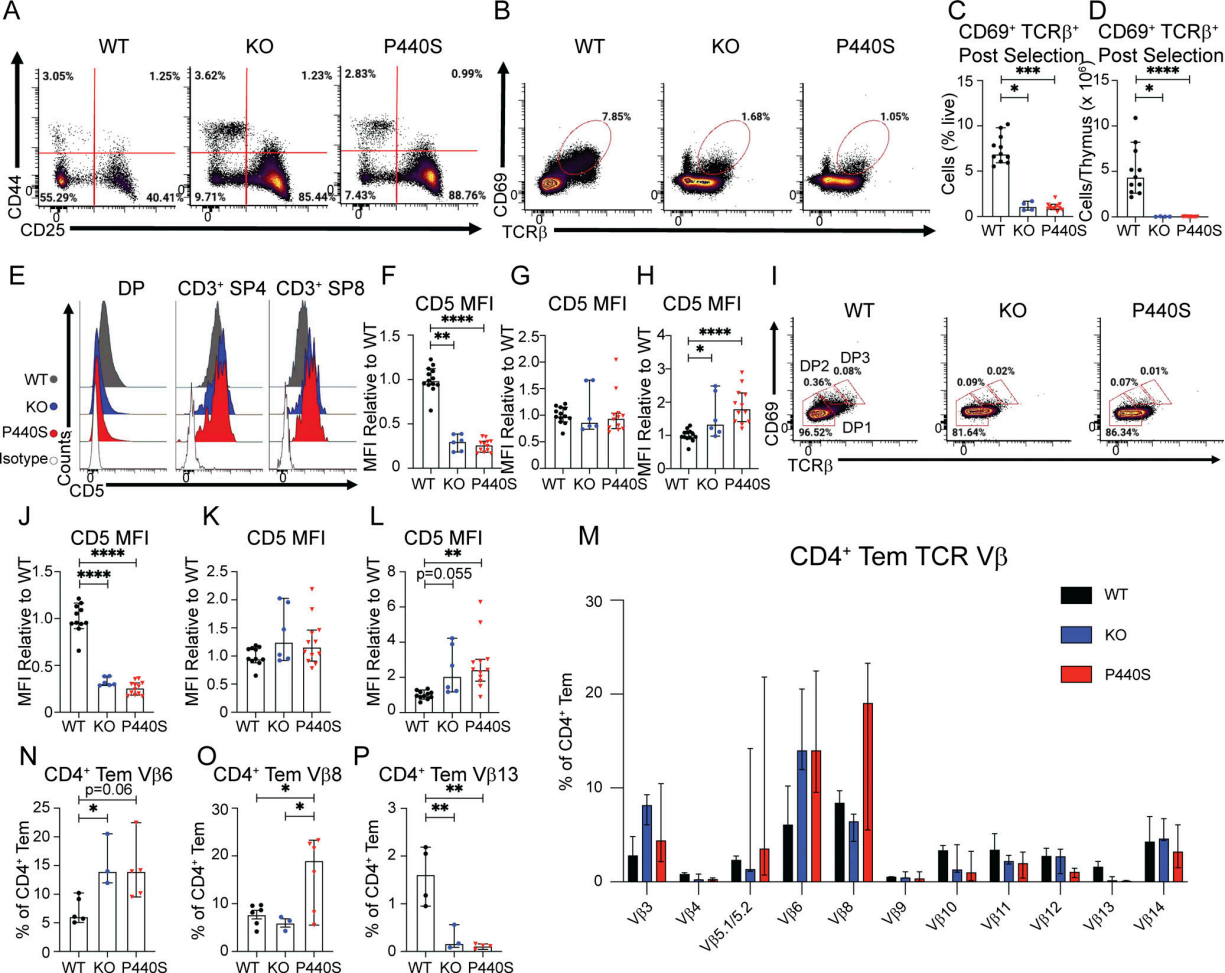
**P440S Lck thymic development supplemental data. (A)** Flow staining of thymic DN subpopulations (CD25^−^CD44^+^ [DN1], CD25^+^CD44^+^ [DN2], CD25^+^CD44^−^ [DN3], CD25^−^CD44^−^ [DN4]). **(B–D)** Flow staining (B), frequency (C), and total counts (D) of postselection thymocytes (CD69^+^TCRβ^+^), gated on live thymocytes. **(E–H)** Surface expression of CD5 on subsets of thymic T cell precursors (CD4^+^CD8^+^ [DP], CD3^+^CD4^+^CD8^−^ [mature SP4], CD3^+^CD4^−^CD8^+^ [mature SP8]). **(I)** Flow staining of DP subpopulations (CD69^lo^TCRβ^lo^ [DP1], CD69^int^TCRβ^int^ [DP2], CD69^hi^TCRβ^hi^ [DP3]), all gated on DP thymocytes. **(J–L)** Surface expression of CD5 on DP1-3 subsets. **(M–P)** TCR Vβ flow assessment of splenic CD62L^lo^CD44^hi^ CD4^+^ Tem. Experiments in A–P are representative of results from three independent experiments with 3–12 mice per group. Error bars represent median and 95% CI. *P < 0.05, **P < 0.01, ***P < 0.001, ****P < 0.0001. Not significant unless stated by asterisk in figure. Ordinary one-way ANOVA with Tukey’s multiple comparisons test was used to test for statistical significance for all experiments.

Compared to WT mice, both P440S and KO mice showed significant thymic hypocellularity (Fig. 5 A) and arrest in T cell development, with significant decreases of double positive (CD4^+^CD8^+^, DP), single positive CD4 (CD3^+^CD4^+^, SP4), single positive CD8 (CD3^+^CD8^+^, SP8), and thymic-derived Tregs (nTregs, Fig. 5, B–I). In particular, there was a halt in the progression from DN stage 3 to DN4 (Fig. 5, J and K; and Fig. S4 A). Additionally, fewer KO and P440S thymocytes underwent positive selection (TCRβ^+^CD69^+^) (Fig. S4, B–D). CD5 is a negative regulator of TCR-mediated signaling and its surface expression by SP thymocytes correlates with positively selecting TCR signal transduction (Tarakhovsky et al., 1995; Azzam et al., 1998). Both KO and P440S DP cells displayed decreased CD5 expression compared with WT (Fig. S4, E–H), specifically by DP1 (CD69^lo^TCRβ^lo^) preselection thymocytes (Fig. S4, I–L). However, while CD5 expression on SP4 thymocytes was similar across genotypes (Fig. S4 G), both KO and P440S T cells had greater CD5 expression by SP8 cells compared with WT (Fig. S4 H). Our findings indicate that Lck deficiency hinders DP and SP thymocyte development considerably and suggest that positive selection signals can still occur in DP thymocytes despite the signaling defect. Assessment of peripheral CD4^+^ Tem TCR Vβ frequency did not show an oligoclonal repertoire (Fig. S4, M–P).

**Figure 6.**
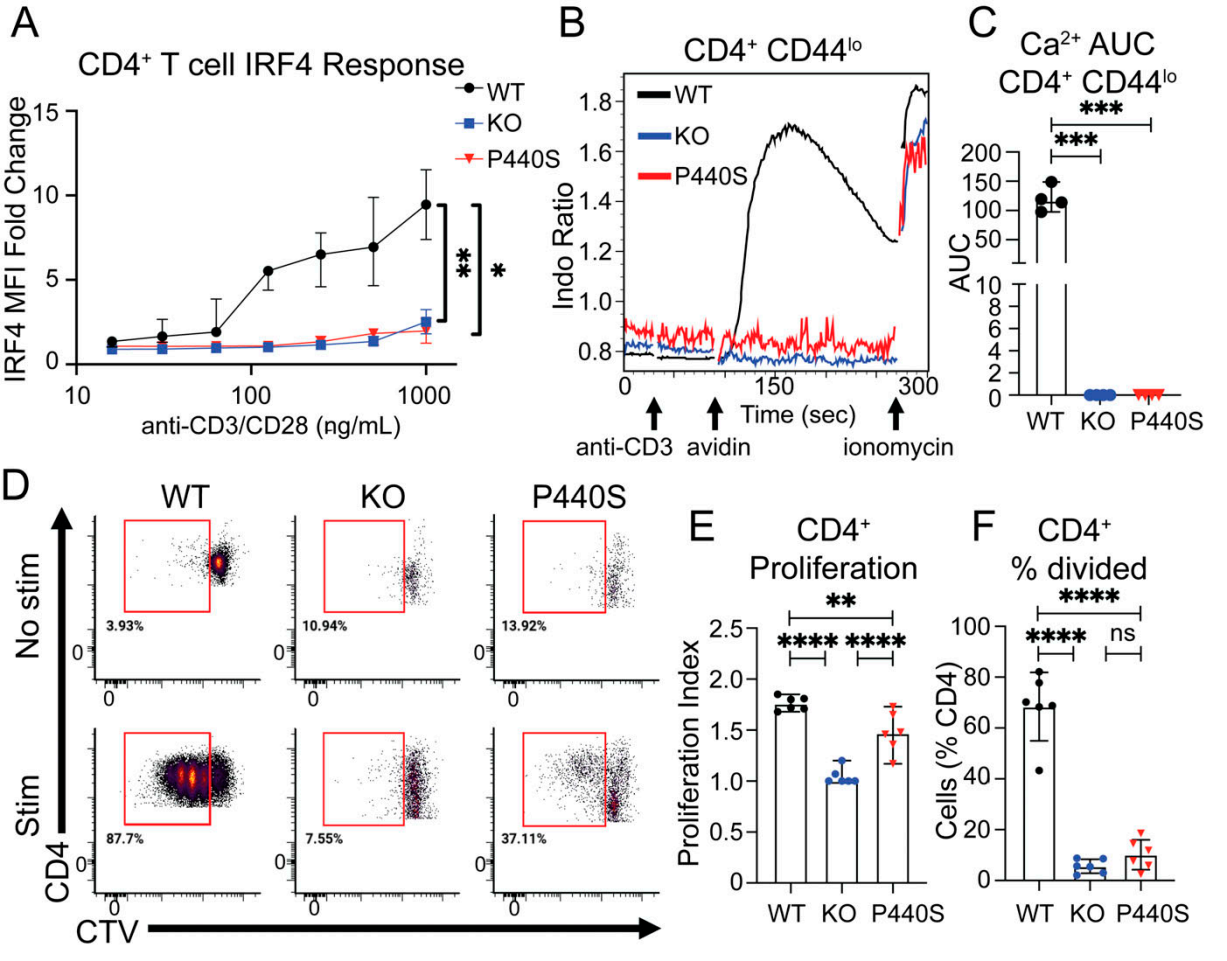
**CD4**^**+**^ **T cells from P440S mice demonstrate increased proliferation compared to KO mice, despite similar TCR signal transduction profiles. (A)** Fold change response of TCR-mediated IRF4 induction was calculated as the mean fluorescence intensity (MFI) ratio of stim/no stim at each concentration of anti-CD3/CD28. **(B)** TCR-mediated calcium mobilization in splenic CD4^+^CD44^lo^ T cells. **(C)** Area under the curve quantitation of the calcium response was calculated for the time between the addition of anti-CD3 and the addition of ionomycin. **(D–F)** Ex vivo proliferation of CTV-loaded splenocytes stimulated with anti-CD3/CD28. Flow plots are gated on live CD4^+^ T cells. All experiments were performed on mice 5–6 wk of age. Experiments in A–F are representative of results from three independent experiments with three to six mice per group. Error bars represent mean and SEM. *P < 0.05, **P < 0.01, ***P < 0.001, ****P < 0.0001. Not significant unless stated by asterisk in figure. Ordinary one-way ANOVA with Tukey’s multiple comparisons test was used to test for statistical significance for all experiments.

We evaluated interferon regulatory factor (IRF) 4 expression in response to anti-CD3/CD28 stimulation of total splenic CD4^+^ T cells and calcium mobilization by splenic CD4^+^ naïve (CD44^lo^) T cells. Compared with the WT, both the KO and P440S CD4^+^ T cells displayed significantly decreased IRF4 expression (Fig. 6 A) and calcium mobilization (Fig. 6, B and C). However, splenic P440S CD4^+^ T cells generated a greater TCR-mediated proliferative response compared with KO CD4^+^ T cells, but both were significantly decreased compared with WT (Fig. 6, D–F). These data are consistent with the patients’ T cell proliferation observed in response to anti-CD3/CD28, where proliferation was significantly decreased but not absent compared with HC (Table 1).

These findings demonstrate that while the KO and P440S mice have similarly defective thymic T cell selection and development, and while their T cells differentiate in the periphery under similar lymphopenic environments, only the partial LOF defect in the P440S mice spares enough T cell function to enable systemic and intestinal tissue inflammation.

### The CD4^+^ T cells in the P440S mice are required to initiate intestinal inflammation

**Figure 7.**
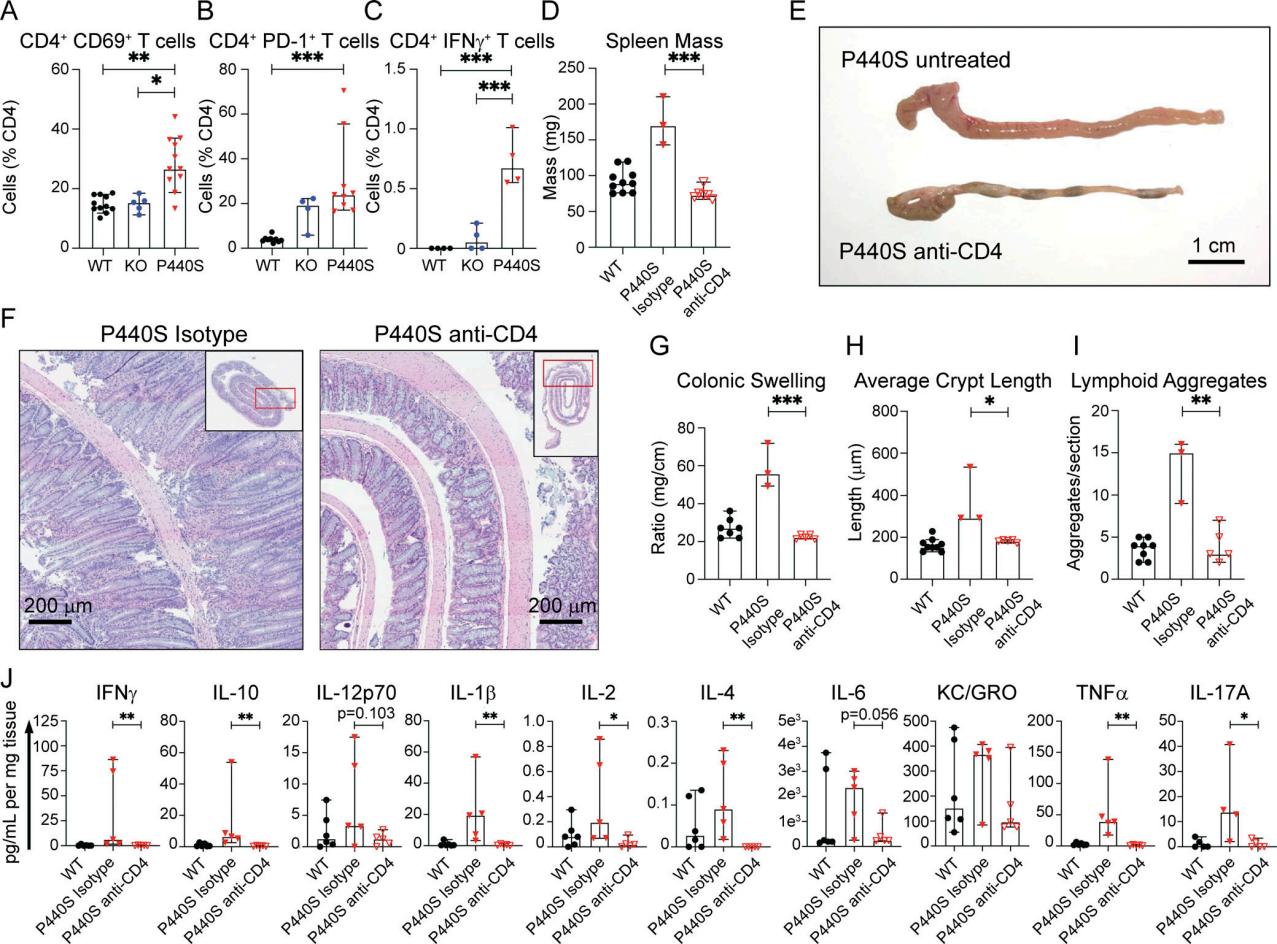
**CD4**^**+**^ **T cells initiate intestinal inflammation in P440S mice. (A and B)** Frequencies of CD4^+^CD69^+^ and CD4^+^PD-1^+^ T cells from mesLN. **(C)** Frequency of IFNγ-producing lamina propria CD4^+^ T cells. **(D)** Whole spleen masses from P440S mice that received CD4-depleting mAb (GK1.5 mAb) or isotype mAb. **(E and F)** Gross (E) and H&E histological (F) images of large intestines from untreated P440S mice, isotype-treated P440S mice, or CD4-depleted P440S mice. **(G)** Ratios of colon length and mass. **(H and I)** Average crypt length (H) and number of lymphoid aggregates per histological section (I) from H&E histology. **(J)** Cytokine concentration from colon culture supernatants. All measurements were taken from mice at 20-wk of age. Length of scale bar in E is 1 cm. Length of bars in F is 200 μm. Experiments in A and B are representative of results from three independent experiments with 4–11 mice per group. Experiments in C–J are representative of results from two independent experiments with 3–10 mice per group. Error bars represent the median and 95% CI. *P < 0.05, **P < 0.01, ***P < 0.001. Not significant unless stated by asterisk in figure. Kruskal–Wallis accounting for multiple comparisons was used for statistical significance testing for experiments in A–C and J. Unpaired *t* test between P440S (isotype) and P440S (anti-CD4) was used to test for statistical significance for experiments in D and G–I.

While the lymphopenic environment exerts pressure on both KO and P440S T cells to undergo proliferation, only the P440S T cells demonstrated the ability to proliferate following TCR stimulation. The P440S proliferative advantage coupled with a thymic selection defect, which likely results in a TCR repertoire skewed toward autoreactivity, may account for the increased T cell lymphocytic infiltrate and cellularity observed in the intestinal tissue of the P440S mice relative to the KO and WT mice (Figs. 3 and 4). Indeed, CD4^+^ T cells from mesLN in P440S mice expressed increased levels of activation markers CD69 and programmed cell death 1 (PD-1) (Fig. 7, A and B). Additionally, P440S CD4^+^ T cells produced IFNγ during active disease (Fig. 7 C), suggesting that the proliferating activated CD4^+^ T cells in P440S mice play a critical role in intestinal inflammation.

**Figure S5.**
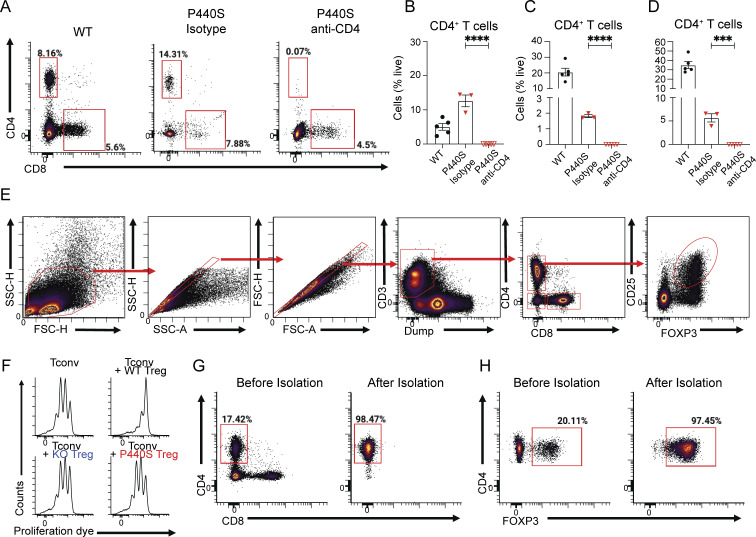
**P440S Lck murine functional supplemental data. (A)** Flow staining of lamina propria cells from CD4 depletion experiments (A), gated on live B220^−^ cells. **(B–D)** Frequencies of CD4^+^ T cells from lamina propria (B), spleen (C), and mesLN (D) from CD4 depletion experiments. **(E)** Flow cytometry gating scheme for splenic CD4^+^CD25^+^FOXP3^+^ Tregs. **(F)** Proliferation dye dilution of WT CD45.1 Tconvs from in vitro Treg suppression assay. **(G and H)** Flow cytometry staining of FACS-sorted donor WT Tregs that were used for Treg transfer experiments. Experiments in B–D are representative of results from two independent experiments with three to five mice per group. Error bars represent median and 95% CI. ***P < 0.001, ****P < 0.0001. Not significant unless stated by asterisk in figure. Unpaired *t* test between P440S (isotype) and P440S (anti-CD4) was used to test for statistical significance in B–D.

To investigate the role of the T cell infiltrate in the intestinal inflammation observed in P440S mice, we evaluated the effect of CD4^+^ T cell depletion on intestinal disease. Weekly injections of anti-CD4 mAb (clone GK1.5) or isotype control mAb began at 4 wk of age, prior to colitis development (typically 10–12 wk), and lasted until 20 wk of age where colitis is typically overt in P440S mice. CD4^+^ T cells were effectively absent from the spleen, mesLN, and lamina propria following this regimen (Fig. S5, A–D). Compared with untreated P440S mice, CD4-depleted P440S mice demonstrated a significant reduction in various measurements of intestinal inflammation (Fig. 7, D–J). In fact, all of these parameters in the CD4-depleted P440S mice were similar to those of WT mice. These findings demonstrate the requirement of CD4^+^ T cells in the initiation of colitis in P440S mice.

### WT Tregs prevent intestinal inflammation development in P440S mice

**Figure 8.**
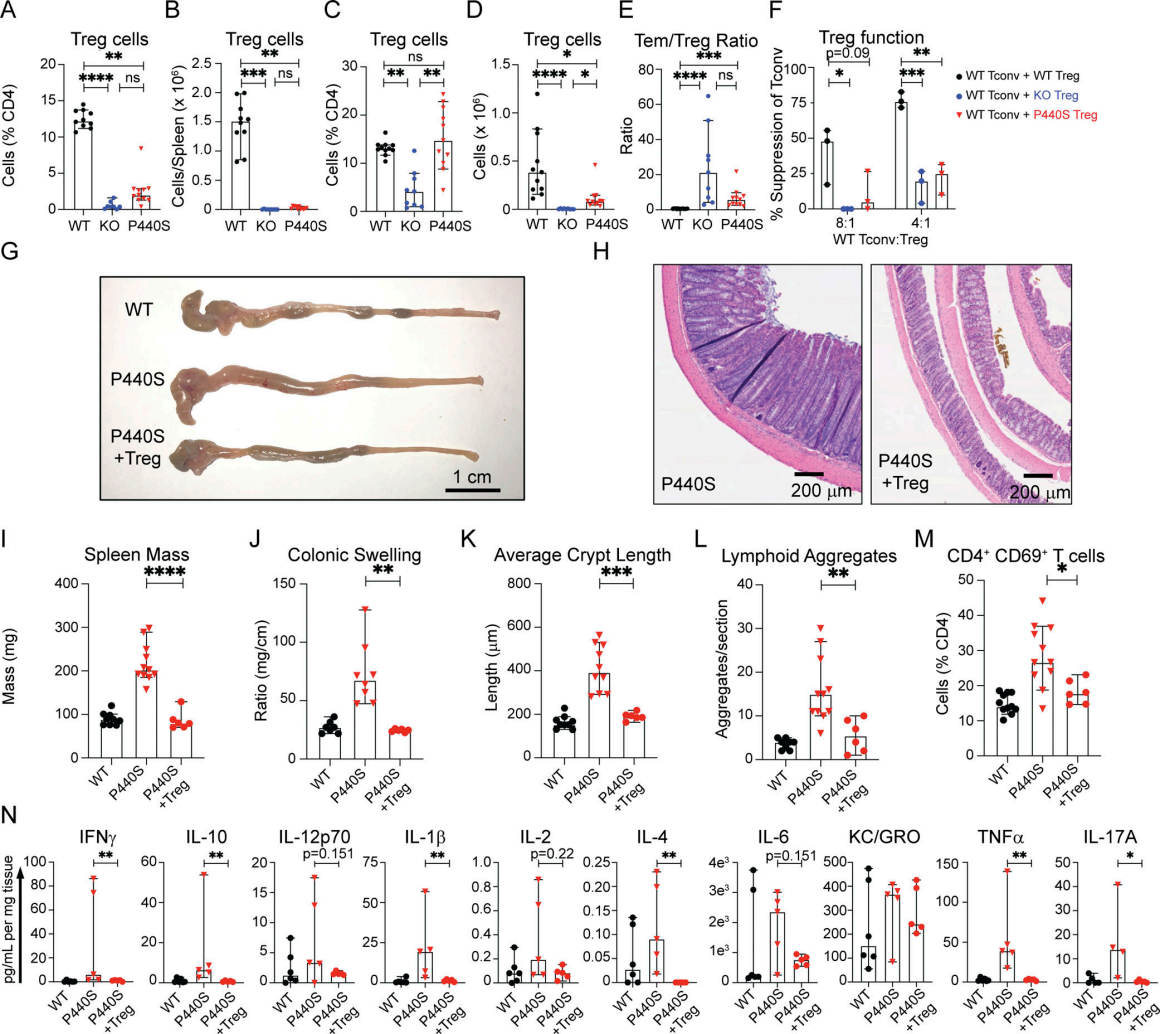
**Regulatory T cell deficiency contributes to P440S intestinal inflammation. (A–D)** Frequencies and counts of Tregs isolated from spleen (A and B) and mesLN (C and D). **(E)** Ratios of the percentage of effector memory (CD62L^lo^CD44^hi^) CD4^+^ T cell to Tregs from mesLN. **(F)** In vitro suppression assay of enriched CD4^+^CD25^+^ Tregs from mice of the indicated genotypes. Tconv are enriched CD4^+^CD25^−^ T cells from WT CD45.1 mice. **(G and H)** Gross (G) and H&E histological (H) images of large intestines from GFP^+^ WT Treg adoptive transfer experiments. **(I)** Whole spleen masses from GFP^+^ WT Treg adoptive transfer experiments. **(J)** Ratios of colon length and mass. **(K and L)** Average crypt length (K) and number of lymphoid aggregates per histological section (L) from H&E histology. **(M)** Frequencies of endogenous (GFP^−^) CD4^+^CD69^+^ T cells in mesLN from Treg adoptive transfer experiments. **(N)** Cytokine concentration from colon culture supernatants. All measurements were taken from mice at 20-wk of age. The length of the scale bar in G is 1 cm. The length of bars in H is 200 μm. Experiments in A–F are representative of results from three independent experiments with 3–11 mice per group. Experiments in G–N are representative of results from two independent experiments with 4–11 mice per group. Error bars represent the median and 95% CI. *P < 0.05, **P < 0.01, ***P < 0.001, ****P < 0.0001. Not significant unless stated by an asterisk in the figure. Kruskal–Wallis accounting for multiple comparisons was used for statistical significance testing in N. Ordinary one-way ANOVA with Tukey’s multiple comparisons test was used to test for statistical significance in A–F. Unpaired *t* test between P440S and P440S (+Treg) was used to test for statistical significance in I–M.

To investigate the possibility that Treg deficiency contributes to P440S intestinal disease, we evaluated the Treg compartment from the spleen and mesLN in WT, KO, and P440S mice. In the spleen, Treg frequency and absolute numbers in P440S and KO mice were reduced compared with WT (but similar to each other) (Fig. 8, A and B; and Fig. S5 E). However, in the mesLN, P440S mice showed increased Treg frequency compared with KO, but both KO and P440S mice have significantly decreased Treg cell numbers than WT (Fig. 8, C and D). Both P440S and KO mice demonstrated an increased ratio of CD4^+^ Tem to Tregs compared with the WT mice (Fig. 8 E). Tregs from KO and P440S mice demonstrated impaired in vitro suppressive activity (Fig. 8 F and Fig. S5 F). However, despite a similarly imbalanced Tem/Treg ratio and defective in vitro Treg function, the KO mice do not develop intestinal inflammation.

Our findings suggest that in the KO mice, neither Treg nor Tem harbor enough functional/proliferative capacity to generate and maintain their regulatory and effector functions. In contrast, the P440S Lck mutant could preserve enough residual Lck activity to allow the function of Tem, but not Treg, in P440S mice. To determine if a functional Treg compartment could alleviate P440S intestinal inflammation, we transferred purified GFP-expressing WT Tregs into 6-wk-old P440S mice and allowed for WT Treg expansion and immunomodulation until 20 wk of age (Fig. S5, G and H). Similar to the results observed with the CD4 depletion, infusion of WT Tregs into P440S mice restore various indicators of intestinal inflammation to those similar in WT mice (Fig. 8, G–N). Thus, a significantly reduced number and function of endogenous Tregs may result in a breakdown of Treg-mediated peripheral tolerance, rendering P440S mice susceptible to intestinal inflammation.

Taken together, our findings indicate that the P440S LCK mutation causes partial LOF. Although attenuated, TCR signal transduction under P440S LCK is sufficient for thymic selection and maturation of some conventional T cells (Tconv) and for partial retention of proliferative capacity. In the periphery, the P440S Tconv become activated under lymphopenic homeostatic pressure and possibly by environmental stimuli from the GI tract. In conjunction with deficient endogenous P440S Treg numbers and function, the unrestrained Tconv consequently drive intestinal inflammation in P440S mice. This contrasts with the KO wherein the complete loss of LCK expression and function causes defective TCR signaling to a degree that is insufficient for the proliferation and function of Tconv and Tregs.

## Discussion

There is growing evidence that mutations that compromise TCR signal strength can differentially affect T cell tolerance and immunity. For example, complete versus partial LOF mutations in ZAP70, LAT, and SLP76 lead to SCID versus CID with immune dysregulation phenotypes, respectively (Elder, 1997; Elder et al., 1994, 1995; Bacchelli et al., 2017; Keller et al., 2016; Lev et al., 2021; Chan et al., 2016; Hsu et al., 2009; Sommers et al., 2002). Immune dysregulation is a negative prognostic factor for survival in patients with CID, even for patients who are eventually treated by HSCT (Fischer et al., 2017). Hence, understanding the degree of T cell function impairment due to such mutations is critical to improving outcomes in CID.

A previously described LCK exon 3 splice mutation caused accelerated LCK mRNA decay with T cell immunodeficiency and severe viral susceptibility without autoimmunity—this mutation could have resulted in a total lack of protein expression, but protein expression and function were not assessed (Li et al., 2016). Another previous report that described an L341P LCK mutation concluded that there was a complete loss of kinase activity, explaining the T cell immunodeficiency (Hauck et al., 2012). However, this patient also displayed skin and joint inflammation. The possibility that residual LCK function remained was not fully excluded. We report a novel human *LCK* variant and unravel the mechanism by which complete versus partial LOF of LCK lead to different disease phenotypes using mouse models harboring the null (KO) or novel human homologous variant (P440S). The P440S LCK variant caused protein structural instability and partial LOF, leading to immunodeficiency due to “hypoactive” T cells (defective TCR-signal transduction) and immune dysregulation due to residual T cell proliferation and cytokine production (CID with autoimmunity). In contrast, the null led to immunodeficiency only.

Residue L341 lies within the αE helix of LCK and the L341P mutation led to decreased protein expression in the patient’s PBMC (Hauck et al., 2012). The P440S variant was expressed at similarly low levels in patients’ PBMC, cell line models, and P440S murine splenic T cells (Figs. 1 and 2; and Fig. S3 A). We explored the basis of decreased P440S LCK protein expression using multiple approaches. Protein structure modeling predicted instability in both (1) the local amino acid region surrounding the P440S variant site and (2) the activation loop that bears Y394 (Fig. 1, D and E). These data suggest disruption of the intramolecular interaction between the two seemingly distant protein regions when P440 is substituted with serine. The P440 and P442 residues within LCK are highly conserved evolutionarily and located in a loop that joins the αF and αG helices (Gonfloni et al., 1997). Due to the unique molecular rigidity of proline, P440 and P442 are likely critical in stabilizing secondary protein structure. Substitution of P440 with serine may confer flexibility to this region that P442 alone cannot maintain, which could result in less stable folding and a shorter protein half-life. We also tested P440S LCK protein directly by measuring protein half-life in cells. This experiment confirmed that the protein has reduced stability.

Consistent with the findings from studies of L341P and P440S LCK cell line data (Fig. 2), both P440S and KO mice demonstrate defective TCR signal transduction (Fig. 6). Yet, P440S mice demonstrate CD4^+^ T cell–dependent intestinal inflammation, suggesting that LOF is only partial (Fig. 7). Consistent with this possibility, P440S CD4^+^ T cells outperformed the KO CD4^+^ T cells in TCR-mediated proliferation studies despite the similar (1) TCR signal transduction defect (Fig. 6), (2) arrest in thymic T cell development (Figs. 5 and S4), (3) and lymphopenic environment in both animal models (Fig. 3). Results of our TCR signaling studies are consistent with those from several other proximal TCR signaling gene defects (Figs. 2, 6, and S2), where weak/residual TCR signaling results in decreased/residual T cell proliferation and immune dysregulation symptomatology (Arnaiz-Villena et al., 1992; Tokgoz et al., 2013; Chan et al., 2016). Similar frequencies of P440S and KO CD4^+^ T cells underwent cell division (Fig. 6 F), suggesting that P440S LCK protein does not impel more CD4^+^ T cells to enter cell division, but it is sufficient to promote a more durable proliferation program.

This capacity for sustained proliferation is likely due to a TCR signal transduction advantage in the P440S compared with the KO, despite similarly defective IRF4 and calcium responses (Fig. 6, A–C). Exclusion of CD45 phosphatase from the contact region of the immunological synapse can shift equilibrium to the phosphorylated state of the TCR complex and ZAP70 by reducing the local phosphatase activity, an effect similar to inhibition using pervanadate, but locally in TCR microclusters rather than throughout the cell (McNeill et al., 2007; Furlan et al., 2014; Varma et al., 2006). As such, TCR crosslinking experiments of P440S LCK cell lines with soluble anti-CD3 likely fail to segregate CD45 from TCR signalosomes and resulted in almost undetectable tyrosine phosphorylation or calcium mobilization (Fig. 2, C and D; and Fig. S2, G–I). When we assessed early TCR signaling events at the immunological synapse using SLBs, we observed TCR-mediated ZAP70 phosphorylation response in the J.CaM 1.6 P440S LCK cell line with significant overlap in the integrated signals from single cells with the empty vector control, but a statistically significant difference (Fig. 2, F–H). This indicates that P440S LCK can promote partial TCR signals in a synaptic context that reach significance at a population level and may provide a mechanistic basis for the in vivo observations.

In addition to its kinase domain, LCK possesses SH2 and SH3 domains that enable it to serve as an adaptor. LCK facilitates ZAP70-mediated phosphorylation of LAT by bridging the two proteins (Lo et al., 2018), and LCK has been proposed to play a similar role in linking CD28 to PKCθ (Kong et al., 2011). In both cases, the ligand for the SH2 domain requires LCK catalytic activity, such that even though the SH2 and SH3 domains of the P440S LCK are intact, the attenuation of the kinase activity would compromise these adapter roles. The lack of detectable P440S LCK recruitment to TCR microclusters is consistent with reduced kinase activity of P440S LCK, also impacting its multivalent interactions with the TCR signalosome. It is important to note that FYN tyrosine kinase shares functions with LCK in T cell development and function, and therefore could compensate to some degree for a proximal TCR-signaling defect (Hauck et al., 2015; Groves et al., 1996). We also observed an absence of P440S LCK Y505 phosphorylation (Fig. S2 C). CSK access to LCK Y505 is controlled by a negative feedback loop, whereby SFK activity results in CSK recruitment to the plasma membrane to regulate SFK function (Brdicka et al., 2000). Defective P440S LCK activity may be insufficient to drive this feedback mechanism, resulting in decreased P440S LCK Y505 phosphorylation, such that P440S LCK may be largely in the open conformation. The open conformation of LCK is targeted for ubiquitination by Casitas B lymphoma-b (Cbl-b), and this may contribute to the shorter half-life of P440S LCK (Rao et al., 2002).

The P440S mouse developed intestinal inflammation spontaneously by 20 wk of age, while the KO mouse did not develop disease (Figs. 3 and 4). *Rag2*^*R229Q*^ hypomorph and *ZAP70*^*skg*^ mouse models exemplify human partial T cell immunodeficiencies wherein CD4^+^ T cells were shown to be necessary for intestinal inflammation and arthritis, respectively (Marrella et al., 2007; Rigoni et al., 2016; Sakaguchi et al., 2003). The efficiency of thymic negative selection is impaired in the absence of Lck (Trobridge et al., 2001). The thymic positive and negative selection thresholds are likely altered in the P440S and KO mice, potentially resulting in the selection of host-reactive T cells. Studies of Treg cells in TCR transgenic mice have revealed exquisite TCR-determined characteristics of the Treg cell population differentiating in the thymus. Experiments by the Hsieh and Lafaille laboratories showed that when the number of precursor cells is dramatically reduced, the expression of a single Treg cell–derived transgenic TCR can drive efficient generation of FOXP3^+^ thymocytes (Bautista et al., 2009; Leung et al., 2009). These results suggest that severe interclonal precursor competition restricts the differentiation of Treg cells expressing TCR of identical specificity and, thus, facilitates generation of a broad Treg TCR repertoire in the thymus (Josefowicz et al., 2012). Therefore, in both the KO and P440S mice, where precursor cells are likely reduced, the TCR-signaling defect likely results in a defective and self-reactivity-biased repertoire along with an altered thymic Treg population. Lymphopenia-induced proliferation likely expands these host-reactive T cells (Moxham et al., 2008; King et al., 2004), but the residual T cell function imparted only by the P440S Lck variant promotes autoinflammation/immunity in the P440S mice.

Consistent with this hypothesis is the observation that effector memory P440S CD4^+^ T cells expanded and/or accumulated in the intestinal draining mesLN, despite the lymphopenia observed in other peripheral lymphoid organs (Fig. 3, G and H). In the mesLN, CD4^+^ Tem absolute counts in the P440S mice are similar to WT mice, but the WT mice do not develop GI inflammation, suggesting that (1) CD4^+^ T cells from P440S mice likely have an autoreactive repertoire and (2) Tregs from P440S mice are likely defective. Indeed, the depletion of CD4^+^ T cells from P440S mice prevented the development of any intestinal inflammation (Fig. 7), which implicates their pathogenicity. We performed adoptive transfers of P440S CD4^+^ T cells into immunodeficient-RAG KO mice to test whether these cells are sufficient to induce colitis (data not shown). Unexpectantly, we did not observe the development of intestinal disease. In this adoptive transfer model of colitis, homeostatic expansion of donor CD4^+^ T cells is requisite for disease induction (Feng et al., 2010). The donor P440S CD4^+^ T cells within RAG KO recipients did not undergo homeostatic expansion, which is likely due to their Lck deficiency (Seddon et al., 2000). The consistent, albeit low, output of P440S CD4^+^ T cells from the thymus may compensate for the failure of these cells to expand in the periphery, which eventually results in disease pathogenesis. Consistent with this hypothesis, P440S mice do not begin to develop intestinal disease when young (4 wk of age) but rather at older age (10–12 wk of age). Treg deficiency may contribute to peripheral tolerance breakdown (Sakaguchi et al., 1995; Takahashi et al., 1998). KO and P440S mice display similarly defective nTreg development, imbalanced peripheral Tem/Treg ratios, and defective in vitro suppressive function (Figs. 5 and 8). Thymic Treg development is preferentially dependent on CD28 (Tang et al., 2003). Lck mediates PKC-θ/CD28 signals (Kong et al., 2011). Thus, defective TCR and CD28 signaling from Lck deficiency profoundly impedes nTreg development. The KO and P440S mice also likely lack the ability to suppress immune responses as a consequence of reduced Treg numbers with compromised TCR signaling (Kim et al., 2009). Without suppression from P440S Tregs, the residual P440S CD4^+^ T cell activity is unrestrained and creates an imbalance between tolerogenic and immunogenic processes, leaving the host susceptible to immune dysregulation when exposed to an “insidious” environmental stimulus, such as gut microbiota.

In the P440S LCK patients, the intestinal inflammation likely arises from a combination of recurrent infection and autoinflammation, processes that are challenging to untangle in the humans. The microbiota in the murine large intestine houses ∼10^10^–10^14^ cells and, along with dietary antigens, provides ample antigenic stimulation of host cells in the colon (Turner, 2018). Indeed, luminal bacteria are a colitogenic driver in mouse models of spontaneous intestinal inflammation (Sellon et al., 1998; Rigoni et al., 2016; Stepankova et al., 2007; Dianda et al., 1997). Therefore, we hypothesize that commensal antigens are the instigators of P440S CD4^+^ T cell residual function, resulting in inflammation of the intestine rather than other tissues. However, further studies involving germ-free environments and/or antimicrobial treatments are needed to understand the contribution of microbiota to the disease pathogenesis. Our mouse model of immune dysregulation due to partial LOF of LCK can be applied to further understand immunopathogenesis seen in the L341P and exon 3 splice variants (Hauck et al., 2012; Li et al., 2016). Inflamed skin lesions on the L341P patient contained infiltrating macrophages and CD3^+^ T cells, which could have arisen as a partial immune response against the skin microbiome and persisted in the absence of regulatory processes. While the L341P was reportedly a functional null mutation as assessed by in vitro cell line assays, an in vivo mouse model may have been necessary to fully evaluate its effect on autoimmune complications.

Intestinal inflammation constitutes the primary clinical symptom in up to 40% of IEI that presents with auto/hyperinflammatory complications (Pazmandi et al., 2019). The intestinal inflammation in these patients is often caused by a combination of infection and autoinflammatory responses (or one process incites the other), and detangling/isolating each process is challenging for the patients. Multiple etiologies contribute to intestinal pathology in monogenic inflammatory bowel disease (IBD), including infection resulting from impaired antimicrobial immunity and disruptions of immune tolerance (Nambu and Muise, 2021). Here, we extended the understanding of the consequences of *LCK* mutations/variants by demonstrating the different phenotypes caused by complete versus partial LOF. Additionally, we provide a mechanistic framework for partial LCK defects as an etiology for monogenic IBD, suggesting TCR signal transduction as a potential target for monogenic IBD therapy.

## Materials and methods

### Patient study enrollment

Patients were referred for HSCT from King Faisal Hospital, Riyadh, Saudi Arabia. Patients (their parents and guardians) were consented and enrolled in a study that was approved by the local review board of Ulm University (Ethikkommission der Universität Ulm), which abides by the principles of the Declaration of Helsinki. HSCT decision was made on a clinical basis. The detection of variants in *LCK* was the result of a next-generation sequencing program (see below), in which patients were included after their cellular therapy. Written informed consent was obtained from the family for the HSCT and for the diagnostic procedures according to the regulations of the review board of Ulm University.

### Whole-exome sequencing

Whole-exome sequencing of probands peripheral blood genomic DNA was performed on an Illumina sequencing platform. Bioinformatic analyses for the detection of rare sequence variants following Mendelian inheritance patterns were performed as described previously (Field et al., 2015). After filtering variants, two candidate genes, iduronate 2-sulfatase (*IDS*) and *LCK*, were considered for evaluation. IDS deficiency was excluded by functional analysis. Confirmation of the segregation of the *LCK* variants considered to be pathogenic was obtained by targeted Sanger sequencing of genomic DNAs of the patients and their parents with the use of Big Dye Terminator (v.1.1) chemistry (Thermo Fisher Scientific) and capillary analyses (ABI 3130XL; Thermo Fisher Scientific). Primers used for sequencing were designed using ENST00000619559.4 as a reference sequence. Variant predictions were performed using the dbNSFP v4.3 database (Liu et al., 2011, 2020; Dong et al., 2015). The filtering strategy for variants was performed as follows: (1) variants detected in both patients, (2) variants that fulfilled OVERALL PASS, (3) variants that were homozygous and hemizygous, (4) variants located in exons ±2 nucleotides, (5) frequency of alleles in ExAC ≤ 0.0001, and (6) CADD score ≥ 10 or N/A.

### Human PBMC immunophenotyping via flow cytometry

PBMC were stained with anti-CD3-BV510 (#300447; BioLegend), anti-CD8-APC (#555369; BD), anti-CD4-PacBlue (#558116; BD), anti-CD19-APC-Cy7 (#561743; BD), and anti-CD56-PE (#318306; BioLegend). Anti-IL-2Rβ-FITC (clone TU27; #11-1228-42; Invitrogen), anti-IL-2Rβ-BV515 (clone Mik-Bta3; #564688; BD), anti-LCK-AF647 (#628304; BioLegend), or mouse IgG1 isotype controls (#554679, #564416; BD, or #MA5-18168; Invitrogen) were applied either before (surface) or after (total) permeabilization. Permeabilization was performed with BD Cytofix/Cytoperm and Perm/Wash buffer (#55471; BD), and intracellular staining was performed after treatment with a transcription factor staining buffer set (#00-5523-00; eBioscience).

### Human PBMC immunoblot

Cryopreserved patient and control PBMC (1 × 10^7^ cells) were stained with anti-CD3-APC (#IM2467; Beckman-Coulter) and anti-CD45-Krome Orange (#B36294; Beckman-Coulter). T cells were sorted with a BD FACSAria II. 3 × 10^5^ T cells (with a purity for CD3^+^ of >99%) from patients and controls were lysed (75 mM Tris/HCl pH 7.5, 150 mM NaCl, 1% NP40, 1% n-dodecyl-β-D-maltoside, 5 mM EDTA) and supplemented with phosphatase inhibitors (PhosSTOP Phosphatase Inhibitor Cocktail, #04 906 837 001; Roche). Defined cell equivalents were loaded on a 10% SDS-PAGE gel, blotted, and probed sequentially with the following antibodies: anti-LCK (#2752; CST), anti-LCK pY505 (#2751; CST), and anti-actin (#ab8227; Abcam). Goat-anti-rabbit IgG-HRP (#170-6515; Bio-Rad) was used as a secondary antibody and SuperSignal West Femto Chemiluminescent substrate (Thermo Fisher Scientific) for detection. Bound antibodies were stripped using Restore Western Blot Stripping Buffer (#21059; Thermo Fisher Scientific).

### Human PBMC immunophenotyping and functional analysis via mass cytometry

Cryopreserved PBMC were thawed and allowed to rest at 37°C for 1 h. Rested PBMC were treated with 1 mM pervanadate (#450243; Sigma-Aldrich) or left untreated. Cells were fixed with 1.6% paraformaldehyde (PFA) (#28908; Thermo Fisher Scientific) before staining. Fixed cells were stored at −80°C and thawed on the day of barcoding and/or staining. The barcoding methodology was adapted from Zunder et al. (2015). Samples were stained with a metal-conjugated antibody panel (Table S4). Data were acquired on a Helios instrument (Fluidigm). To make all samples maximally comparable, data were acquired using internal metal isotope bead standards and normalized as previously described (Finck et al., 2013). Files were debarcoded using the Matlab Debarcoder Tool (Zunder et al., 2015). Data were analyzed with FlowJo and Cytobank.

### Protein structure 3D in silico protein modeling

The WT and P440S mutants of LCK have been simulated by means of molecular dynamics in both forms (open/active and closed/inactive). The starting structures have been taken from the corresponding WT Lck x-ray ones (PDB ID: 3LCK, for the active state; 2PL0, for the inactive state), and the P440S variant has been included by molecular modeling by means of Chimera UCSF Software. The molecular dynamics simulations have been performed using the Gromacs software (v.2016) package in the canonical ensemble. The proteins have been solvated using the SPC water model. After energy minimization steps, 200 ns long molecular dynamics simulations have been performed using a cut-off of 1.2 nm, particle mesh Ewald method to treat the long-range electrostatic interactions, and a time-step of 2 fs. The temperature was kept fixed at 310 K using the velocity rescaling algorithm.

### Molecular dynamics simulations

The WT LCK and P440S LCK simulation systems were built starting from the structure containing residues 231–502 in PDB 1QPC (Zhu et al., 1999); the ATP analog present in the structure was not retained. Approximately 22,000 TIP3P water molecules were added along with NaCl at a concentration of 150 mM (71 Na^+^ and 62 Cl^−^) using CHARMM-GUI (Jo et al., 2008). The resulting system sizes were ∼70,000 atoms and 85–95 Å along each of the x, y, and z dimensions. Simulations were run using NAMD3 on GPUs (Phillips et al., 2020) and the CHARMM36m force field for proteins (Huang et al., 2017). A time step of 2 fs was used. A cutoff of 12 Å and a switching function beginning at 10 Å were employed for short-range non-bonded interactions, while long-range electrostatic interactions were evaluated every second-time step using the particle mesh Ewald method (Darden et al., 1993). Temperature was maintained at 310 K using Langevin dynamics and pressure was maintained at 1 atm using a Langevin piston. Simulations for each of the WT and P440S were run for 1 μs in triplicate (6 μs in total). Visualization and analysis were carried out using VMD (Humphrey et al., 1996).

### Lentiviral transduction of J.CaM 1.6 cell lines

Healthy control and P440S *LCK* patient mRNA were obtained from PBMCs using TRIzol extraction (#15596026; Thermo Fisher Scientific) and then converted to cDNA (#A5000; Promega). The WT *LCK* and P440S *LCK* cDNA sequences were amplified by PCR and then cloned into the pWPI bicistronic IRES-GFP lentiviral vector (plasmid #12254; Addgene). The WT *LCK*-pWPI, P440S *LCK*-pWPI, and empty pWPI vectors were transfected along with helper plasmids pMD2.G (plasmid #12259; Addgene) and psPAX2 (plasmid #12260; Addgene) into LentiX 293 cells (#632180; Takara) using Lipofectamine 3000 (#L3000001; Thermo Fisher Scientific). The lentiviral supernatants were collected 24 h after transfection. Resultant lentiviral supernatants were used to transduce J.CaM 1.6 cells (#CRL-2063; ATCC). FACS sorting was then performed to isolate low-, medium-, and high-GFP-expressing transductants.

### Jk.LckKO cell lines and inducible expression system

Human Jurkat T cells with a genetic deletion of *LCK* introduced using CRISPR/Cas9 (Jk.LckKO cells) were previously described and were kindly provided by Prof. Dr. Art Weiss (University of California, San Francisco, San Francisco, CA, USA) (Courtney et al., 2017). The plix402_hLckWT plasmid containing a tetracycline-controlled transactivator, a tetracycline-responsive promoter, and the coding sequence for WT human LCK was a generous gift from Oreste Acuto (Sir William Dunn School of Pathology, University of Oxford, Oxford, UK). The P440S LCK mutant was generated by mutagenesis PCR. Lentiviral particles were produced by cotransfection of plix402_hLck constructs, pCMVDR8.74, and pMD2G plasmids using PEI (Polysciences) in HEK293T cells. Viruses were concentrated by centrifugation and used to transduce Jk.LckKO cells. In detail, 3 × 10^6^ cells per condition were cultivated in RPMI medium containing 5% Tet System-approved FCS, and lentiviruses were added in the presence of 5 μg/ml of polybrene. Cells were incubated overnight at 37°C. On the next day, the medium was exchanged. LCK expression was induced by incubation with 3 μg/ml doxycycline for 24 h at 37°C. Induced expression of LCK was assayed by intracellular flow cytometry. To this end, cells were fixed and permeabilized using BD Cytofix/Cytoperm kit and were incubated with a rabbit anti-LCK antibody (Cell Signaling) overnight. The next day, cells were washed and incubated with a goat anti-rabbit IgG-Dylight 633 antibody for 30 min before analyzing them in a Cyan ADP Flow cytometer (Beckman Coulter).

### Cell line TCR activation assays

Transduced J.CaM 1.6 cell lines were placed on ice and then treated with 10 μg/ml biotinylated anti-CD3 (#13-0037-82; eBioscience), 10 μg/ml anti-CD28 (#14-0289-82; eBioscience), and 1 μg/ml avidin (#A9275; Sigma-Aldrich). The treated cell lines were then submerged into a 37°C water bath for activation, and at the indicated time points, the stimulations were halted by the addition of NP40 lysis buffer (1% NP-40 in 10 mM Trish pH 7.4, 150 mM NaCl supplemented with 1 mM PMSF, 10 nM NaF, 100 μg/ml aprotinin, 100 μg/ml α-1-antitrypsin, 100 μg/ml leupeptin, 2 mM NaVO_3_, and 10 mM tetrasodium pyrophosphate). Supernatants from the lysates were then treated with Laemmli sample buffer (250 mM Tris pH 6.8, 2% [wt/vol] SDS, 10% [vol/vol] glycerol, 5% [vol/vol] β-mercaptoethanol, 0.2% [wt/vol] bromophenol blue) and then stored at −20°C until protein gel electrophoresis.

For the inducible Jk.LckKO cell line system, LCK expression was induced in Jk.LckKO cells as described above. TCR stimulation was performed at 37°C with 5 μg/ml of the anti-human CD3ε antibody OKT3 for the indicated times or left unstimulated. Cells were then fixed and permeabilized using BD Cytofix/Cytoperm kit, and intracellular staining was performed as described above using the phospho-p44/42 MAPK (ERK1/2) (Thr202/Tyr204) antibody from Cell Signaling followed by incubation with a goat anti-rabbit IgG-Dylight 633 antibody and analysis in a Gallios Flow cytometer (Beckman Coulter).

### Immunoblot of J.CaM 1.6 cell lines and murine T cells

Whole-cell lysates were electrophoresed under denaturing conditions by SDS-PAGE and transferred to polyvinylidene difluoride using semidry blotting conditions. Blocking was performed using 3% BSA in Tris-buffered saline with Tween (10 mM Tris-HCl pH 8.0, 150 mM NaCl, and 0.05% Tween). Antibodies used for blotting include anti-LCK (#2752; CST), anti-actin HRP (#sc47778; SBT), anti-LCK pY505 (#2751; CST), anti-pTyr clone 4G10, anti-CSK (#sc-386; SBT), anti-FYN (#4023T; CST), anti-CD3ζ pY142 (#68235; Abcam), anti-CD3ζ (#644101; BioLegend), anti-ZAP70 pY319 (#2701; CST), anti-ZAP70 (2705; CST), anti-PLCγ1 pY783 (14008; CST), anti-PLCγ1 (#5690; CST), anti-ERK pT202/T204 (#9101; CST), anti-ERK (#4695; CST), anti-mouse IgG-HRP (#7076; CST), and anti-rabbit IgG HRP (#7074S; CST). Bound antibodies were stripped using stripping buffer (62.5 mM Tris-HCl pH 6.8, 2% SDS, 0.8% β-mercaptoethanol). Blots were visualized using Pierce SuperSignal West Pico PLUS (#34580; Thermo Fisher Scientific). Immunoblot band intensities were quantified using Image Studio Lite.

### Generation of P440S Lck mice

*LCK* P440S knock-in mice were generated using CRISPR technology at the National Jewish Mouse Genetics Core Facility. The target sequence for the single-guide RNA (sgRNA) is 5′-CTG​GGT​AAG​GGA​TTC​GAC​CGT​GG-3′. The ssDNA oligo used as the HDR template is 5′-GCT​TGG​CTC​CCC​TTC​CTT​GAA​GAC​TTA​GAG​TTG​CTT​GTC​TTA​AGG​AAA​GCT​CAC​CTG​GGT​AAG​AGA​TTC​GAC​CGT​GTG​TGA​CGA​TCT​CTG​TAA​GCA​GGA​TCC​CGA​AGG​ACC​ACA​CGT​CTG​ACT​TGA​TGG​TGA​AGG​TCC​CAT​AGT​TAA​TGG​CTT​CTG​GTG-3′. Zygotes from C57BL/6 mice were injected with sgRNA, HDR oligo, and CAS9 protein (1081060; IDT) and then transferred to surrogate mothers. One pup with the desired P440S variant was used as the founder mouse to backcross onto C57BL/6 background. Primers used for genotyping include forward primer 5′-CTG​AGC​TGC​AAG​ATT​GCA​GAC-3′, reverse primer 5′-GCA​GAG​ATG​GAA​TGA​AGC​ATC-3′. Digestion using the BamHI restriction enzyme cleaves a unique restriction site introduced by HDR oligo. PCR products following BamHI digestion indicate mouse genotype: WT = 455 bp; heterozygous mutant = 455 + 220 + 235 bp; and homozygous mutant = 220 + 235 bp.

### Mice

All mice were bred and maintained in a specific pathogen–free facility. C57BL/6 (#000664; JAX), Lck KO (#002817; JAX), and FOXP3-GFP (#035864; JAX) were all purchased from Jackson Laboratory and bred in-house. All experiments were approved by the Institutional Animal Care and Use Committee of the University of Colorado Anschutz Medical Campus (#000914; Protocol). All efforts were made to minimize mouse suffering.

### Murine immunophenotyping via flow cytometry

Murine spleen and lymph nodes were mechanically dissociated in complete RPMI (RPMI 1640 media [#15040CV; Corning] supplemented with 10% fetal bovine serum [#PS-FB3; Peak Serum] and 1× penicillin–streptomycin–glutamine [#10378016; Gibco]), filtered, then RBC-lysed (#10128-802; VMR). After washing with PBS, 3 × 10^6^ cells were labeled with fixable viability dye and then treated with Fc Block clone 2.4G2 (in-house hybridoma). Cells were then surface stained with fluorochrome-conjugated antibodies for 30 min in the dark at 4°C, then fixed and permeabilized (#00-5523-00; eBioscience) for intracellular staining with fluorochrome-conjugated antibodies overnight at 4°C. Data acquisition was performed on a Cytek Aurora multispectral flow cytometer and analyzed using CellEngine. Flow cytometry support was provided by the University of Colorado Anschutz Medical Campus ImmunoMicro Flow Cytometry Shared Resource (RRID: SCR_021321). Fixable viability dyes used include Zombie Green (#423111; BioLegend) and Ghost Dye Red 780 (13-0865; Cytek). Antibodies used for staining include anti-CD4-BV510 (#563106; BD), anti-CD8-AF700 (#100729; BioLegend), anti-CD44-PerCPCy5.5 (#560570; BD), anti-CD62L-PE-Cy7 (#25-0621-82; Invitrogen), anti-TCRχδ-BUV395 (#744118; BD), anti-CD5-PE (#553022; BD), anti-CD69-BV605 (#104529; BioLegend), anti-TCRβ-BV421 (#109229; BioLegend), anti-CD3-BUV563 (#741448; BD), anti-CD25-BV711 (#740714; BD), anti-TER-119-APC/Cy7 (#116223; BD), anti-CD11b-APC/Cy7 (#101225; BD), anti-NK1.1-APC/Cy7 (#108723; BD), anti-Ly-6G-APC/Cy7 (#127623; BD), anti-CD19-APC/Cy7 (#561737; BD), anti-FOXP3-AF647 (#560401; BD), and anti-PD-1-PE/CF594 (#562523; BD). TCR Vβ flow assessment was performed using BD Pharmingen anti-mouse TCR Vβ screening panel (#557004; BD), anti-Vβ6-PE (#140003; BioLegend), anti-Vβ8-PE (#553862; BD), anti-Vβ10-PE (#553285; BD), anti-Vβ11-PE (#553198; BD), and anti-Vβ12-PE (#139703; BioLegend).

### Lamina propria isolation

Murine large intestines were resected and then flushed of luminal contents with PBS. The intraepithelial lymphocyte fraction was separated by vortexing the colon in complete RPMI supplemented with 1 mM EDTA and 0.2 mM dithiothreitol for 30 min. The remaining intestinal tissue was minced and digested with 1 mg/ml Collagenase Type 4 (#LS004186; Worthington Biochemical), 10 µg/ml DNAse I (#11284932001; Sigma-Aldrich) in complete RPMI at 37°C for 45 min. The digestion solution was filtered and the cellular layer was isolated by Ficoll-Paque density gradient separation (#17-1440-02; Cytiva).

### FACS sorting of murine splenic T cells

Single-cell suspensions of murine splenocytes were stained with anti-CD4 Alexa Fluor 488 (#100425; BioLegend) and anti-CD8 BV510 (#100751; BioLegend) and were FACS-sorted on a BD FACSAria Fusion. The sorted CD4^+^ and CD8^+^ T cells were lysed with 1% NP-40 in 10 mM Trish pH 7.4, 150 mM NaCl supplemented with 1 mM PMSF, 10 nM NaF, 100 μg/ml aprotinin, 100 μg/ml α-1-antitrypsin, 100 μg/ml leupeptin, 2 mM NaVO3, and 10 mM tetrasodium pyrophosphate. Supernatants from the lysates were then treated with Laemmli sample buffer (250 mM Tris pH 6.8, 2% [wt/vol] SDS, 10% [vol/vol] glycerol, 5% [vol/vol] β-mercaptoethanol, 0.2% [wt/vol] bromophenol blue) and then stored at −20°C until protein gel electrophoresis.

### Intracellular cytokine staining

Brefeldin A (0.5 mg/ml, #AGCN20018M025; AdipoGen) was injected into mice (500 μl/mouse i.v.). Following 4 h, the mice were euthanized and single-cell suspensions of the lamina propria were isolated as described above. Cells were labeled with a fixable viability dye and then treated with Fc Block clone 2.4G2 (in-house hybridoma). Cells were then treated with fluorochrome-conjugated antibodies for 30 min in the dark at 4°C, then fixed and permeabilized (#554722; BD) for intracellular staining with fluorochrome-conjugated antibodies overnight at 4°C (#557885; BD). The fixable viability dye used was Ghost Dye Red 780 (13-0865; Cytek). Antibodies used for staining include anti-CD45-BV510 (#103137; BioLegend), anti-CD4-PE (#100511; BioLegend), anti-CD8-BUV737 (#612759; BD), anti-CD3-APC (#100235; BioLegend), anti-TNFα-eFluor450 (#48-7321-82; Invitrogen), anti-IFNγ-PerCPCy5.5 (#560660; BD), and anti-IL-17A-BV711 (#506941; BioLegend).

### Serum and gut supernatant cytokine measurements

Blood samples were collected by cardiac puncture and the blood was allowed to coagulate at room temperature. Following centrifugation, the serum supernatants were collected and stored at −20°C for future experiments. Colonic supernatants were collected by resecting a small segment (10–15 mg) of the colon, incubating the segment in complete RPMI media overnight, and then storing the supernatant at −20°C for future experiments. Quantitation of IFNγ, IL-10, IL12-p70, IL-1β, IL-2, IL-4, IL-6, KC-GRO, and TNFα was performed using Meso Scale Diagnostics V-PLEX Proinflammatory Panel 1 Mouse Kit (#K15048D; MSD). Measurement of IL-17 was performed using BioLegend ELISA MAX Standard Set Mouse IL-17A (#432501; BioLegend) according to the manufacturer’s protocol.

### Measurement of intracellular calcium concentration

Transduced J.CaM 1.6 cell lines were stained with a fixable viability dye and 5 μM indo-1 acetoxymethyl ester (Indo-1 AM, #I1223; Thermo Fisher Scientific). Indo-1 was excited using a 355 nm UV laser and calcium-bound Indo-1 was detected with a 379/28 bandpass filter while calcium-unbound Indo-1 was detected with a 450/50 bandpass filter. Intracellular calcium concentration was calculated as the ratio of calcium-bound/unbound over time. Labeled cells were warmed to 37°C prior to stimulation. Samples were first recorded without treatment for 30 s to establish a baseline, then recorded for 1 min following labeling with 1 μg/ml biotinylated anti-CD3 (#13-0037-82; eBioscience), then recorded for 3 min following crosslinking with 100 μg/ml avidin (#A9275; Sigma-Aldrich), and then recorded for 30 s following treatment with 1 μg/ml ionomycin (#407950; Calbiochem). Data acquisition was performed on an LSRFortessa X-20 and analyzed using FlowJo.

For the inducible Jk.LckKO cell line system, LCK expression was induced in Jk.LckKO cells as described above. 10^6^ cells in the medium containing 1% Tet System-approved FCS were incubated for 45 min at 37°C in the dark with Fura red (Thermo Fisher Scientific) and Fluo-3 (Invitrogen). Cells were then washed and mixed with prewarmed medium just before recording in a Cyan ADP Flow cytometer (Beckman Coulter). After 1 min recording, cells were stimulated with 3 μg/ml of OKT3 antibody and recording continued for another 5 min.

Murine splenocytes were lysed of RBC and depleted of B220^+^ cells using biotinylated anti-B220 (#103204; BioLegend) and magnetic streptavidin nanobeads (#480016; BioLegend). The resulting cells were stained with fixable viability dye, 5 μM indo-1 acetoxymethyl ester (Indo-1 AM, #I1223; Thermo Fisher Scientific), anti-CD4-PE (#50-0042-U100; Cytek), anti-CD8-APC (#100711; BioLegend), and anti-CD44-PerCP Cy5.5 (#560570; BD). Labeled cells were warmed to 37°C prior to stimulation. Samples were first recorded without treatment for 30 s to establish a baseline, then recorded for 1 min following labeling with 0.13 mg/ml biotinylated anti-CD3 (#30-0031-U500; Tonbo), then recorded for 3 min following crosslinking with 100 μg/ml avidin (#A9275; Sigma-Aldrich), and then recorded for 30 s following treatment with 1 μg/ml ionomycin (#407950; Calbiochem). Data acquisition was performed on an LSRFortessa X-20 and analyzed using FlowJo.

### Protein stability assay

Transduced J.CaM 1.6 cell lines were incubated in complete RPMI media supplemented with 32 μg/ml cycloheximide (#01810; Sigma-Aldrich) at 37°C. At the indicated time points, the treated cells were lysed with NP40 Lysis buffer (1% NP-40 in 10 mM Trish pH 7.4, 150 mM NaCl supplemented with 1 mM PMSF, 10 nM NaF, 100 μg/ml aprotinin, 100 μg/ml α-1-antitrypsin, 100 μg/ml leupeptin, 2 mM NaVO3, and 10 mM tetrasodium pyrophosphate). Supernatants from the lysates were then treated with Laemmli sample buffer (250 mM Tris pH 6.8, 2% [wt/vol] SDS, 10% [vol/vol] glycerol, 5% [vol/vol] β-mercaptoethanol, and 0.2% [wt/vol] bromophenol blue) and then stored at −20°C until protein gel electrophoresis.

### Preparation of SLB and immunocytochemistry

To prepare SLBs, glass coverslips (Nexterion) were plasma cleaned and mounted onto six-channel chambers (Ibidi). Small unilamellar liposomes were prepared using 1,2-dioleoyl-sn-glycero-3-phosphocholine (Avanti Polar Lipids, Inc.) supplemented with 12.5% 1,2-dioleoyl-sn-glycero-3-[(N-(5-amino-1-carboxypentyl) iminodiacetic acid) succinyl]-Ni (Avanti Polar Lipids, Inc.). Channels in Ibidi chamber were covered with liposome mixture, blocked, and washed. SLB were then incubated with the indicated mix of His-tagged proteins to achieve the desired density of molecules on the SLB: anti-CD3 (UCHT1, 30 molecules/μm^2^) and ICAM1 (200 molecules/μm^2^), CD80 (200 molecules/μm^2^) and CD58 (200 molecules/μm^2^). His-tagged proteins were produced in-house and, when indicated, were conjugated with the AlexaFluor dyes of interest (488). To generate the synapses, transduced J.CaM 1.6 cells were exposed to the bilayers at 37°C for 10 min, fixed with 2% PFA, and washed. For immunocytochemistry, samples were permeabilized, blocked, and incubated with the primary antibody of interest (anti-LCK; CST Technologies; anti-pZAP70-PE; BD Biosciences) overnight at 4°C. Samples were further incubated with a secondary antibody (anti-Rabbit Ig-AF647) for 1 h at room temperature.

### TIRF microscopy and image analysis

TIRF imaging was performed on an Olympus IX83 inverted microscope with a TIRF module. The instrument was equipped with an Olympus UApON 150× TIRF N.A 1.45 objective, 405, 488, 568, and 640 nm laser lines, and Photometrics Evolve delta EMCCD camera. Image analysis was performed using Fiji (ImageJ). Briefly, synaptic contacts were segmented based on the interference reflection image (IRM) and the mean gray values for LCK and pZAP70 were measured.

### Murine T cell activation assays

For measurement of IRF4 induction, 24-well cell culture plates were coated overnight with anti-CD3 (#70-0031-U500; Tonbo) and anti-CD28 (#70-0281-U100; Tonbo) at the indicated concentrations. Murine splenocytes were RBC-lysed (#10128-802; VMR), plated onto stimulation plates at 3 × 10^6^ cells/ml, and then incubated at 37°C for 16 h. Cells were then processed for flow cytometric analysis as described above.

For measurement of cell proliferation, 12-well cell culture plates were coated overnight with anti-CD3 (#70-0031-U500; Tonbo) and anti-CD28 (#70-0281-U100; Tonbo) at 1 μg/ml each. Murine splenocytes were RBC-lysed (#10128-802; VMR) and stained with CellTrace Violet (CTV) Proliferation Dye (#C34557; Invitrogen). CTV-loaded splenocytes were plated onto stimulation plates at 3 × 10^6^ cells/ml and then incubated at 37°C for 72 h. Splenocytes were then processed for flow cytometric analysis as described above. The proliferation index was calculated using FloJo.

### Intestinal permeability assay

Mice were fasted for 4.5 h and then given oral gavage of 150 μl 100 mg/ml FITC-dextran (#46944; Sigma-Aldrich). At 2- and 4-h following gavage, blood was collected via cheek bleed and diluted with equal parts 5 mM EDTA. FITC fluorescence intensity within plasma samples was measured (excitation max: 490 nm, emission max: 525 nm) on a Tecan Infinite 200 PRO plate reader.

### In vivo depletion of CD4^+^ T cells

Mice were administered weekly injections of anti-CD4 mAb clone GK1.5 (#BE0003-1, 200 μg/mouse, i.p.; BioXCell) or isotype control (#BE0090, 200 μg/mouse, i.p.; BioXCell) beginning at 4 wk of age. These weekly injections were performed until recipient mice reached 20 wk of age, at which point the mice were euthanized and analyzed. Mice were bled via cheek bleed 1 mo following the first injection to verify depletion of CD4^+^ T cells.

### In vitro Treg suppression assay

Treg cells (CD4^+^CD25^+^) were enriched from pooled lymphoid organs of WT, KO, or P440S mice using mouse regulatory T cell isolation kit (#130-091-041; Miltenyi). 500,000 purified CTV-labeled CD45.1 WT Tconv cells (CD4^+^CD25^−^) were added to a 96-well plate coated with anti-CD3 (#70-0031-U500; Tonbo) and anti-CD28 (#70-0281-U100; Tonbo) at 1 µg/ml each. Tconv were cocultured with or without Tregs for 72 h. The proliferation of Tconv was analyzed by flow cytometry. Percent suppression was calculated using the equation: 100 − (x/y)100, where x = division index of Tconv with Treg and y = division index of Tconv without Treg.

### Adoptive transfer of Tregs

Splenocytes from FOXP3-GFP mice were harvested, RBC-lysed, and magnetically enriched for CD4^+^ T cells (#480033; BioLegend). The enriched CD4^+^ T cells were further FACS-sorted for GFP expression to obtain a purified fraction of CD4^+^ GFP^+^ Tregs (purity >95%). Purified GFP^+^ Tregs were resuspended and injected into P440S mice that were 6 wk of age (2.5 × 10^5^ cells/mouse, i.v.). Recipient P440S mice were aged to 20 wk, at which point they were euthanized and analyzed.

### Histology

Histological preparation was performed by the University of Colorado Anschutz Medical Campus Research Histology Section (RRID: SCR_021994). Murine colons were fixed in 10% neutral-buffered formalin (REF 5725; Thermo Fisher Scientific) and embedded with paraffin. Slides were then stained with H&E. Whole slide scans of tissue stained with H&E were collected on a Vectra Polaris using 20× objective and analyzed using Phenochart (Akoya Biosciences). Individual slide images of indicated genotypes were stitched together to create the final composite image. Average crypt length was calculated by randomly sampling crypts throughout the entire colon, measuring the length (μm) of each crypt from base to apex, and then averaging the lengths for each colon. Lymphoid aggregates per slide were manually counted.

Multispectral imaging was performed through our collaboration with the Human Immune Monitoring Shared Resource (HIMSR) at the University of Colorado School of Medicine using the Akoya Biosciences Vectra 3.0 instrument. To quantify immune cells, slides of murine colons were stained consecutively with antibodies specific for the following proteins: anti-F4/80 (#30325S; CST), anti-B220 (#550286; BD), anti-CD3 (#99940; CST), anti-CD8 (#98941S; CST), anti-CD4 (14-9766-82; Invitrogen), anti-FOXP3 (14-5773-82; Invitrogen), and DAPI counterstain. The slides were stained on the Leica Bond RX autostainer according to standard protocols provided by Leica and Akoya Biosciences and performed routinely by the HIMSR. Briefly, the slides were deparaffinized, heat-treated in antigen retrieval buffer, blocked, and incubated with primary antibody, followed by HRP-conjugated secondary antibody polymer, and HRP-reactive OPAL fluorescent reagents that use TSA chemistry to deposit dyes on the tissue immediately surrounding each HRP molecule. To prevent further deposition of fluorescent dyes in subsequent staining steps, the slides were stripped in between each stain with heat treatment in an antigen retrieval buffer. Whole-slide scans were collected using the 10× objective and multispectral images of each tissue were collected using the 20× objective with a 0.5-μm resolution. The seven-color images were analyzed with inForm software to unmix adjacent fluorochromes; subtract autofluorescence; segment the tissue; compare the frequency and location of cells in colons stromal areas; segment cellular membrane, cytoplasm, and nuclear regions; score each cellular compartment for expression of each marker, and phenotype infiltrating immune cells according to morphology and cell marker expression.

### Statistical analysis

Statistical tests used are indicated in the figure legends. All analyses were calculated using GraphPad Prism and all plots show the median ± 95% CI. When data did not meet assumptions for the parametric statistical test (normality, equal variance, independence, and no extreme outliers), the non-parametric alternative was used.

### Online supplemental material

Fig. S1 shows patient computerized tomography (CT) scan and TCR repertoire. Fig. S2 shows P440S LCK cell line supplemental data. Fig. S3 shows P440S Lck mouse phenotype supplemental data. Fig. S4 shows P440S Lck thymic development supplemental data. Fig. S5 shows P440S Lck murine functional supplemental data. Table S1 shows details of HSCT. Table S2 shows chimerism and immunological reconstitution after transplantation. Table S3 shows whole exome sequencing variants. Table S4 shows the features of the LCK P440S variant. Table S5 shows the mass cytometry antibody staining panel.

## Supplementary Material

Table S1 shows details of HSCT.

Table S2 shows chimerism and immunological reconstitution after transplantation.

Table S3 shows whole-exome sequencing variants.

Table S4 shows the features of the LCK P440S variant.

Table S5 shows the mass cytometry antibody staining panel.

SourceData F1 contains original blots for Fig. 1.

SourceData F2 contains original blots for Fig. 2.

SourceData FS2 contains original blots for Fig. S2.

SourceData FS3 contains original blots for Fig. S3.

## Data Availability

All data in the figures are available in the published article and in online supplemental material. Unprocessed images of immunoblots and FCS files from mass cytometry and flow cytometry experiments are openly available in Mendeley Data at doi:10.17632/n8658z8fg5.2 (Lui and Hsieh, 2023).

## References

[bib1] Abraham, N., and A. Veillette. 1990. Activation of p56lck through mutation of a regulatory carboxy-terminal tyrosine residue requires intact sites of autophosphorylation and myristylation. Mol. Cell. Biol. 10:5197–5206. 10.1128/MCB.10.10.5197 1697929 PMC361199

[bib2] Abraham, N., M.C. Miceli, J.R. Parnes, and A. Veillette. 1991. Enhancement of T-cell responsiveness by the lymphocyte-specific tyrosine protein kinase p56lck. Nature. 350:62–66. 10.1038/350062a0 1706070

[bib3] Amrein, K.E., and B.M. Sefton. 1988. Mutation of a site of tyrosine phosphorylation in the lymphocyte-specific tyrosine protein kinase, p56lck, reveals its oncogenic potential in fibroblasts. Proc. Natl. Acad. Sci. USA. 85:4247–4251. 10.1073/pnas.85.12.4247 3380789 PMC280404

[bib4] Arnaiz-Villena, A., M. Timon, A. Corell, P. Perez-Aciego, J.M. Martin-Villa, and J.R. Regueiro. 1992. Brief report: Primary immunodeficiency caused by mutations in the gene encoding the CD3-gamma subunit of the T-lymphocyte receptor. N. Engl. J. Med. 327:529–533. 10.1056/NEJM199208203270805 1635567

[bib5] Arpaia, E., M. Shahar, H. Dadi, A. Cohen, and C.M. Roifman. 1994. Defective T cell receptor signaling and CD8^+^ thymic selection in humans lacking zap-70 kinase. Cell. 76:947–958. 10.1016/0092-8674(94)90368-9 8124727

[bib6] Ashouri, J.F., W.L. Lo, T.T.T. Nguyen, L. Shen, and A. Weiss. 2022. ZAP70, too little, too much can lead to autoimmunity. Immunol. Rev. 307:145–160. 10.1111/imr.13058 34923645 PMC8986586

[bib7] Azzam, H.S., A. Grinberg, K. Lui, H. Shen, E.W. Shores, and P.E. Love. 1998. CD5 expression is developmentally regulated by T cell receptor (TCR) signals and TCR avidity. J. Exp. Med. 188:2301–2311. 10.1084/jem.188.12.2301 9858516 PMC2212429

[bib8] Bacchelli, C., F.A. Moretti, M. Carmo, S. Adams, H.C. Stanescu, K. Pearce, M. Madkaikar, K.C. Gilmour, A.K. Nicholas, C.G. Woods, . 2017. Mutations in linker for activation of T cells (LAT) lead to a novel form of severe combined immunodeficiency. J. Allergy Clin. Immunol. 139:634–642.e5. 10.1016/j.jaci.2016.05.036 27522155

[bib9] Bautista, J.L., C.W.J. Lio, S.K. Lathrop, K. Forbush, Y. Liang, J. Luo, A.Y. Rudensky, and C.S. Hsieh. 2009. Intraclonal competition limits the fate determination of regulatory T cells in the thymus. Nat. Immunol. 10:610–617. 10.1038/ni.1739 19430476 PMC2756247

[bib10] Bergman, M., T. Mustelin, C. Oetken, J. Partanen, N.A. Flint, K.E. Amrein, M. Autero, P. Burn, and K. Alitalo. 1992. The human p50csk tyrosine kinase phosphorylates p56lck at Tyr-505 and down regulates its catalytic activity. EMBO J. 11:2919–2924. 10.1002/j.1460-2075.1992.tb05361.x 1639064 PMC556773

[bib11] Brdicka, T., D. Pavlistová, A. Leo, E. Bruyns, V. Korínek, P. Angelisová, J. Scherer, A. Shevchenko, I. Hilgert, J. Cerný, . 2000. Phosphoprotein associated with glycosphingolipid-enriched microdomains (PAG), a novel ubiquitously expressed transmembrane adaptor protein, binds the protein tyrosine kinase csk and is involved in regulation of T cell activation. J. Exp. Med. 191:1591–1604. 10.1084/jem.191.9.1591 10790433 PMC2213442

[bib12] Chan, A.C., T.A. Kadlecek, M.E. Elder, A.H. Filipovich, W.L. Kuo, M. Iwashima, T.G. Parslow, and A. Weiss. 1994. ZAP-70 deficiency in an autosomal recessive form of severe combined immunodeficiency. Science. 264:1599–1601. 10.1126/science.8202713 8202713

[bib13] Chan, A.Y., D. Punwani, T.A. Kadlecek, M.J. Cowan, J.L. Olson, E.F. Mathes, U. Sunderam, S.M. Fu, R. Srinivasan, J. Kuriyan, . 2016. A novel human autoimmune syndrome caused by combined hypomorphic and activating mutations in ZAP-70. J. Exp. Med. 213:155–165. 10.1084/jem.20150888 26783323 PMC4749924

[bib14] Chiang, Y.J., and R.J. Hodes. 2016. T-cell development is regulated by the coordinated function of proximal and distal Lck promoters active at different developmental stages. Eur. J. Immunol. 46:2401–2408. 10.1002/eji.201646440 27469439 PMC5183457

[bib15] Chow, L.M., M. Fournel, D. Davidson, and A. Veillette. 1993. Negative regulation of T-cell receptor signalling by tyrosine protein kinase p50csk. Nature. 365:156–160. 10.1038/365156a0 8371758

[bib16] Courtney, A.H., J.F. Amacher, T.A. Kadlecek, M.N. Mollenauer, B.B. Au-Yeung, J. Kuriyan, and A. Weiss. 2017. A phosphosite within the SH2 domain of Lck regulates its activation by CD45. Mol. Cell. 67:498–511.e6. 10.1016/j.molcel.2017.06.024 28735895 PMC5558854

[bib17] Crequer, A., A. Troeger, E. Patin, C.S. Ma, C. Picard, V. Pedergnana, C. Fieschi, A. Lim, A. Abhyankar, L. Gineau, . 2012. Human RHOH deficiency causes T cell defects and susceptibility to EV-HPV infections. J. Clin. Invest. 122:3239–3247. 10.1172/JCI62949 22850876 PMC3428089

[bib18] Dadi, H.K., A.J. Simon, and C.M. Roifman. 2003. Effect of CD3delta deficiency on maturation of alpha/beta and gamma/delta T-cell lineages in severe combined immunodeficiency. N. Engl. J. Med. 349:1821–1828. 10.1056/NEJMoa031178 14602880

[bib19] Darden, T., D. York, and L. Pedersen. 1993. Particle mesh Ewald: An N log(N) method for Ewald sums in large systems. J. Chem. Phys. 98:10089–10092. 10.1063/1.464397

[bib20] Davis, S.J., and P.A. van der Merwe. 2006. The kinetic-segregation model: TCR triggering and beyond. Nat. Immunol. 7:803–809. 10.1038/ni1369 16855606

[bib21] de la Calle-Martin, O., M. Hernandez, J. Ordi, N. Casamitjana, J.I. Arostegui, I. Caragol, M. Ferrando, M. Labrador, J.L. Rodriguez-Sanchez, and T. Espanol. 2001. Familial CD8 deficiency due to a mutation in the CD8 alpha gene. J. Clin. Invest. 108:117–123. 10.1172/JCI10993 11435463 PMC209336

[bib22] de Saint Basile, G., F. Geissmann, E. Flori, B. Uring-Lambert, C. Soudais, M. Cavazzana-Calvo, A. Durandy, N. Jabado, A. Fischer, and F. Le Deist. 2004. Severe combined immunodeficiency caused by deficiency in either the δ or the ε subunit of CD3. J. Clin. Invest. 114:1512–1517. 10.1172/JCI200422588 15546002 PMC525745

[bib23] Dianda, L., A.M. Hanby, N.A. Wright, A. Sebesteny, A.C. Hayday, and M.J. Owen. 1997. T cell receptor-alpha beta-deficient mice fail to develop colitis in the absence of a microbial environment. Am. J. Pathol. 150:91–97. 9006326 PMC1858528

[bib24] Dong, C., P. Wei, X. Jian, R. Gibbs, E. Boerwinkle, K. Wang, and X. Liu. 2015. Comparison and integration of deleteriousness prediction methods for nonsynonymous SNVs in whole exome sequencing studies. Hum. Mol. Genet. 24:2125–2137. 10.1093/hmg/ddu733 25552646 PMC4375422

[bib25] Douglass, A.D., and R.D. Vale. 2005. Single-molecule microscopy reveals plasma membrane microdomains created by protein-protein networks that exclude or trap signaling molecules in T cells. Cell. 121:937–950. 10.1016/j.cell.2005.04.009 15960980 PMC2851620

[bib26] Eck, M.J., S.K. Atwell, S.E. Shoelson, and S.C. Harrison. 1994. Structure of the regulatory domains of the Src-family tyrosine kinase Lck. Nature. 368:764–769. 10.1038/368764a0 7512222

[bib27] Elder, M.E. 1997. SCID due to ZAP-70 deficiency. J. Pediatr. Hematol. Oncol. 19:546–550. 10.1097/00043426-199711000-00014 9407944

[bib28] Elder, M.E., D. Lin, J. Clever, A.C. Chan, T.J. Hope, A. Weiss, and T.G. Parslow. 1994. Human severe combined immunodeficiency due to a defect in ZAP-70, a T cell tyrosine kinase. Science. 264:1596–1599. 10.1126/science.8202712 8202712

[bib29] Elder, M.E., T.J. Hope, T.G. Parslow, D.T. Umetsu, D.W. Wara, and M.J. Cowan. 1995. Severe combined immunodeficiency with absence of peripheral blood CD8^+^ T cells due to ZAP-70 deficiency. Cell. Immunol. 165:110–117. 10.1006/cimm.1995.1193 7671314

[bib30] Feng, T., L. Wang, T.R. Schoeb, C.O. Elson, and Y. Cong. 2010. Microbiota innate stimulation is a prerequisite for T cell spontaneous proliferation and induction of experimental colitis. J. Exp. Med. 207:1321–1332. 10.1084/jem.20092253 20498021 PMC2882839

[bib31] Field, M.A., V. Cho, M.C. Cook, A. Enders, C.G. Vinuesa, B. Whittle, T.D. Andrews, and C.C. Goodnow. 2015. Reducing the search space for causal genetic variants with VASP. Bioinformatics. 31:2377–2379. 10.1093/bioinformatics/btv135 25755272 PMC4495293

[bib32] Filipp, D., B.L. Leung, J. Zhang, A. Veillette, and M. Julius. 2004. Enrichment of lck in lipid rafts regulates colocalized fyn activation and the initiation of proximal signals through TCR alpha beta. J. Immunol. 172:4266–4274. 10.4049/jimmunol.172.7.4266 15034040

[bib33] Finck, R., E.F. Simonds, A. Jager, S. Krishnaswamy, K. Sachs, W. Fantl, D. Pe’er, G.P. Nolan, and S.C. Bendall. 2013. Normalization of mass cytometry data with bead standards. Cytometry A. 83:483–494. 10.1002/cyto.a.22271 23512433 PMC3688049

[bib34] Fischer, A., J. Provot, J.P. Jais, A. Alcais, N. Mahlaoui, and members of the CEREDIH French PID study group. 2017. Autoimmune and inflammatory manifestations occur frequently in patients with primary immunodeficiencies. J. Allergy Clin. Immunol. 140:1388–1393.e8. 10.1016/j.jaci.2016.12.978 28192146

[bib35] Furlan, G., T. Minowa, N. Hanagata, C. Kataoka-Hamai, and Y. Kaizuka. 2014. Phosphatase CD45 both positively and negatively regulates T cell receptor phosphorylation in reconstituted membrane protein clusters. J. Biol. Chem. 289:28514–28525. 10.1074/jbc.M114.574319 25128530 PMC4192502

[bib36] Gil, J., E.M. Busto, B. Garcillán, C. Chean, M.C. García-Rodríguez, A. Díaz-Alderete, J. Navarro, J. Reiné, A. Mencía, D. Gurbindo, . 2011. A leaky mutation in CD3D differentially affects αβ and γδ T cells and leads to a Tαβ-Tγδ^+^B^+^NK^+^ human SCID. J. Clin. Invest. 121:3872–3876. 10.1172/JCI44254 21926461 PMC3195454

[bib37] Gonfloni, S., J.C. Williams, K. Hattula, A. Weijland, R.K. Wierenga, and G. Superti-Furga. 1997. The role of the linker between the SH2 domain and catalytic domain in the regulation and function of Src. EMBO J. 16:7261–7271. 10.1093/emboj/16.24.7261 9405355 PMC1170326

[bib38] Groves, T., P. Smiley, M.P. Cooke, K. Forbush, R.M. Perlmutter, and C.J. Guidos. 1996. Fyn can partially substitute for Lck in T lymphocyte development. Immunity. 5:417–428. 10.1016/S1074-7613(00)80498-7 8934569

[bib39] Hauck, F., C. Randriamampita, E. Martin, S. Gerart, N. Lambert, A. Lim, J. Soulier, Z. Maciorowski, F. Touzot, D. Moshous, . 2012. Primary T-cell immunodeficiency with immunodysregulation caused by autosomal recessive LCK deficiency. J. Allergy Clin. Immunol. 130:1144–1152.e11. 10.1016/j.jaci.2012.07.029 22985903

[bib40] Hauck, F., B. Blumenthal, S. Fuchs, C. Lenoir, E. Martin, C. Speckmann, T. Vraetz, W. Mannhardt-Laakmann, N. Lambert, M. Gil, . 2015. SYK expression endows human ZAP70-deficient CD8 T cells with residual TCR signaling. Clin. Immunol. 161:103–109. 10.1016/j.clim.2015.07.002 26187144

[bib41] Horkova, V., A. Drobek, D. Paprckova, V. Niederlova, A. Prasai, V. Uleri, D. Glatzova, M. Kraller, M. Cesnekova, S. Janusova, . 2023. Unique roles of co-receptor-bound LCK in helper and cytotoxic T cells. Nat. Immunol. 24:174–185. 10.1038/s41590-022-01366-0 36564464 PMC9810533

[bib42] Hsu, L.Y., Y.X. Tan, Z. Xiao, M. Malissen, and A. Weiss. 2009. A hypomorphic allele of ZAP-70 reveals a distinct thymic threshold for autoimmune disease versus autoimmune reactivity. J. Exp. Med. 206:2527–2541. 10.1084/jem.20082902 19841086 PMC2768860

[bib43] Huang, J., S. Rauscher, G. Nawrocki, T. Ran, M. Feig, B.L. de Groot, H. Grubmüller, and A.D. MacKerell Jr. 2017. CHARMM36m: An improved force field for folded and intrinsically disordered proteins. Nat. Methods. 14:71–73. 10.1038/nmeth.4067 27819658 PMC5199616

[bib44] Huck, K., O. Feyen, T. Niehues, F. Rüschendorf, N. Hübner, H.J. Laws, T. Telieps, S. Knapp, H.H. Wacker, A. Meindl, . 2009. Girls homozygous for an IL-2-inducible T cell kinase mutation that leads to protein deficiency develop fatal EBV-associated lymphoproliferation. J. Clin. Invest. 119:1350–1358. 10.1172/JCI37901 19425169 PMC2673872

[bib45] Humphrey, W., A. Dalke, and K. Schulten. 1996. VMD: Visual molecular dynamics. J. Mol. Graph. 14:33–38. 10.1016/0263-7855(96)00018-5 8744570

[bib46] Jo, S., T. Kim, V.G. Iyer, and W. Im. 2008. CHARMM-GUI: A web-based graphical user interface for CHARMM. J. Comput. Chem. 29:1859–1865. 10.1002/jcc.20945 18351591

[bib47] Josefowicz, S.Z., L.F. Lu, and A.Y. Rudensky. 2012. Regulatory T cells: Mechanisms of differentiation and function. Annu. Rev. Immunol. 30:531–564. 10.1146/annurev.immunol.25.022106.141623 22224781 PMC6066374

[bib48] Keller, B., I. Zaidman, O.S. Yousefi, D. Hershkovitz, J. Stein, S. Unger, K. Schachtrup, M. Sigvardsson, A.A. Kuperman, A. Shaag, . 2016. Early onset combined immunodeficiency and autoimmunity in patients with loss-of-function mutation in LAT. J. Exp. Med. 213:1185–1199. 10.1084/jem.20151110 27242165 PMC4925012

[bib49] Kim, P.W., Z.Y.J. Sun, S.C. Blacklow, G. Wagner, and M.J. Eck. 2003. A zinc clasp structure tethers Lck to T cell coreceptors CD4 and CD8. Science. 301:1725–1728. 10.1126/science.1085643 14500983

[bib50] Kim, J.K., M. Klinger, J. Benjamin, Y. Xiao, D.J. Erle, D.R. Littman, and N. Killeen. 2009. Impact of the TCR signal on regulatory T cell homeostasis, function, and trafficking. PLoS One. 4:e6580. 10.1371/journal.pone.0006580 19668367 PMC2719063

[bib51] King, C., A. Ilic, K. Koelsch, and N. Sarvetnick. 2004. Homeostatic expansion of T cells during immune insufficiency generates autoimmunity. Cell. 117:265–277. 10.1016/S0092-8674(04)00335-6 15084263

[bib52] Kong, K.F., T. Yokosuka, A.J. Canonigo-Balancio, N. Isakov, T. Saito, and A. Altman. 2011. A motif in the V3 domain of the kinase PKC-θ determines its localization in the immunological synapse and functions in T cells via association with CD28. Nat. Immunol. 12:1105–1112. 10.1038/ni.2120 21964608 PMC3197934

[bib53] Kupfer, A., and S.J. Singer. 1989. The specific interaction of helper T cells and antigen-presenting B cells. IV. Membrane and cytoskeletal reorganizations in the bound T cell as a function of antigen dose. J. Exp. Med. 170:1697–1713. 10.1084/jem.170.5.1697 2530300 PMC2189515

[bib54] Kupfer, A., S.L. Swain, C.A. Janeway Jr., and S.J. Singer. 1986. The specific direct interaction of helper T cells and antigen-presenting B cells. Proc. Natl. Acad. Sci. USA. 83:6080–6083. 10.1073/pnas.83.16.6080 3526350 PMC386442

[bib55] Leung, M.W.L., S. Shen, and J.J. Lafaille. 2009. TCR-dependent differentiation of thymic Foxp3^+^ cells is limited to small clonal sizes. J. Exp. Med. 206:2121–2130. 10.1084/jem.20091033 19737865 PMC2757883

[bib56] Leupin, O., R. Zaru, T. Laroche, S. Müller, and S. Valitutti. 2000. Exclusion of CD45 from the T-cell receptor signaling area in antigen-stimulated T lymphocytes. Curr. Biol. 10:277–280. 10.1016/S0960-9822(00)00362-6 10712909

[bib57] Lev, A., Y.N. Lee, G. Sun, E. Hallumi, A.J. Simon, K.S. Zrihen, S. Levy, T. Beit Halevi, M. Papazian, N. Shwartz, . 2021. Inherited SLP76 deficiency in humans causes severe combined immunodeficiency, neutrophil and platelet defects. J. Exp. Med. 218:e20201062. 10.1084/jem.20201062 33231617 PMC7690938

[bib58] Li, S.L., L.N. Duo, H.J. Wang, W. Dai, E.Y.H. Zhou, Y.N. Xu, T. Zhao, Y.Y. Xiao, L. Xia, Z.H. Yang, . 2016. Identification of LCK mutation in a family with atypical epidermodysplasia verruciformis with T-cell defects and virus-induced squamous cell carcinoma. Br. J. Dermatol. 175:1204–1209. 10.1111/bjd.14679 27087313

[bib59] Liu, X., X. Jian, and E. Boerwinkle. 2011. dbNSFP: A lightweight database of human nonsynonymous SNPs and their functional predictions. Hum. Mutat. 32:894–899. 10.1002/humu.21517 21520341 PMC3145015

[bib60] Liu, X., C. Li, C. Mou, Y. Dong, and Y. Tu. 2020. dbNSFP v4: A comprehensive database of transcript-specific functional predictions and annotations for human nonsynonymous and splice-site SNVs. Genome Med. 12:103. 10.1186/s13073-020-00803-9 33261662 PMC7709417

[bib61] Lo, W.L., N.H. Shah, N. Ahsan, V. Horkova, O. Stepanek, A.R. Salomon, J. Kuriyan, and A. Weiss. 2018. Lck promotes Zap70-dependent LAT phosphorylation by bridging Zap70 to LAT. Nat. Immunol. 19:733–741. 10.1038/s41590-018-0131-1 29915297 PMC6202249

[bib62] Lovatt, M., A. Filby, V. Parravicini, G. Werlen, E. Palmer, and R. Zamoyska. 2006. Lck regulates the threshold of activation in primary T cells, while both Lck and Fyn contribute to the magnitude of the extracellular signal-related kinase response. Mol. Cell. Biol. 26:8655–8665. 10.1128/MCB.00168-06 16966372 PMC1636771

[bib63] Lui, V., and E. Hsieh. 2023. A partial human LCK defect causes a T cell immunodeficiency with intestinal inflammation. Mendeley Data, V2. 10.17632/n8658z8fg5.2 PMC10644909 37962568

[bib64] Marrella, V., P.L. Poliani, A. Casati, F. Rucci, L. Frascoli, M.L. Gougeon, B. Lemercier, M. Bosticardo, M. Ravanini, M. Battaglia, . 2007. A hypomorphic R229Q Rag2 mouse mutant recapitulates human Omenn syndrome. J. Clin. Invest. 117:1260–1269. 10.1172/JCI30928 17476358 PMC1857243

[bib65] McNeill, L., R.J. Salmond, J.C. Cooper, C.K. Carret, R.L. Cassady-Cain, M. Roche-Molina, P. Tandon, N. Holmes, and D.R. Alexander. 2007. The differential regulation of Lck kinase phosphorylation sites by CD45 is critical for T cell receptor signaling responses. Immunity. 27:425–437. 10.1016/j.immuni.2007.07.015 17719247

[bib66] Molina, T.J., K. Kishihara, D.P. Siderovski, W. van Ewijk, A. Narendran, E. Timms, A. Wakeham, C.J. Paige, K.U. Hartmann, A. Veillette, . 1992. Profound block in thymocyte development in mice lacking p56lck. Nature. 357:161–164. 10.1038/357161a0 1579166

[bib67] Morgan, N.V., S. Goddard, T.S. Cardno, D. McDonald, F. Rahman, D. Barge, A. Ciupek, A. Straatman-Iwanowska, S. Pasha, M. Guckian, . 2011. Mutation in the TCRα subunit constant gene (TRAC) leads to a human immunodeficiency disorder characterized by a lack of TCRαβ^+^ T cells. J. Clin. Invest. 121:695–702. 10.1172/JCI41931 21206088 PMC3026716

[bib68] Moxham, V.F., J. Karegli, R.E. Phillips, K.L. Brown, T.T. Tapmeier, R. Hangartner, S.H. Sacks, and W. Wong. 2008. Homeostatic proliferation of lymphocytes results in augmented memory-like function and accelerated allograft rejection. J. Immunol. 180:3910–3918. 10.4049/jimmunol.180.6.3910 18322199

[bib69] Mustelin, T., K.M. Coggeshall, and A. Altman. 1989. Rapid activation of the T-cell tyrosine protein kinase pp56lck by the CD45 phosphotyrosine phosphatase. Proc. Natl. Acad. Sci. USA. 86:6302–6306. 10.1073/pnas.86.16.6302 2548204 PMC297826

[bib70] Nambu, R., and A.M. Muise. 2021. Advanced understanding of monogenic inflammatory bowel disease. Front Pediatr. 8:618918. 10.3389/fped.2020.618918 33553075 PMC7862769

[bib71] Ostergaard, H.L., D.A. Shackelford, T.R. Hurley, P. Johnson, R. Hyman, B.M. Sefton, and I.S. Trowbridge. 1989. Expression of CD45 alters phosphorylation of the lck-encoded tyrosine protein kinase in murine lymphoma T-cell lines. Proc. Natl. Acad. Sci. USA. 86:8959–8963. 10.1073/pnas.86.22.8959 2530588 PMC298410

[bib72] Pazmandi, J., A. Kalinichenko, R.C. Ardy, and K. Boztug. 2019. Early-onset inflammatory bowel disease as a model disease to identify key regulators of immune homeostasis mechanisms. Immunol. Rev. 287:162–185. 10.1111/imr.12726 30565237 PMC7379380

[bib73] Pelchen-Matthews, A., I. Boulet, D.R. Littman, R. Fagard, and M. Marsh. 1992. The protein tyrosine kinase p56lck inhibits CD4 endocytosis by preventing entry of CD4 into coated pits. J. Cell Biol. 117:279–290. 10.1083/jcb.117.2.279 1373141 PMC2289416

[bib74] Phillips, J.C., D.J. Hardy, J.D.C. Maia, J.E. Stone, J.V. Ribeiro, R.C. Bernardi, R. Buch, G. Fiorin, J. Hénin, W. Jiang, . 2020. Scalable molecular dynamics on CPU and GPU architectures with NAMD. J. Chem. Phys. 153:044130. 10.1063/5.0014475 32752662 PMC7395834

[bib75] Piątosa, B., B. Wolska-Kuśnierz, M. Pac, K. Siewiera, E. Gałkowska, and E. Bernatowska. 2010. B cell subsets in healthy children: Reference values for evaluation of B cell maturation process in peripheral blood. Cytometry B Clin. Cytom. 78:372–381. 10.1002/cyto.b.20536 20533385

[bib76] Rao, N., S. Miyake, A.L. Reddi, P. Douillard, A.K. Ghosh, I.L. Dodge, P. Zhou, N.D. Fernandes, and H. Band. 2002. Negative regulation of Lck by Cbl ubiquitin ligase. Proc. Natl. Acad. Sci. USA. 99:3794–3799. 10.1073/pnas.062055999 11904433 PMC122603

[bib77] Rigoni, R., E. Fontana, S. Guglielmetti, B. Fosso, A.M. D’Erchia, V. Maina, V. Taverniti, M.C. Castiello, S. Mantero, G. Pacchiana, . 2016. Intestinal microbiota sustains inflammation and autoimmunity induced by hypomorphic RAG defects. J. Exp. Med. 213:355–375. 10.1084/jem.20151116 26926994 PMC4813669

[bib78] Roifman, C.M., H. Dadi, R. Somech, A. Nahum, and N. Sharfe. 2010. Characterization of ζ-associated protein, 70 kd (ZAP70)-deficient human lymphocytes. J. Allergy Clin. Immunol. 126:1226–1233.e1. 10.1016/j.jaci.2010.07.029 20864151

[bib79] Sakaguchi, S., N. Sakaguchi, M. Asano, M. Itoh, and M. Toda. 1995. Immunologic self-tolerance maintained by activated T cells expressing IL-2 receptor alpha-chains (CD25). Breakdown of a single mechanism of self-tolerance causes various autoimmune diseases. J. Immunol. 155:1151–1164. 10.4049/jimmunol.155.3.1151 7636184

[bib80] Sakaguchi, N., T. Takahashi, H. Hata, T. Nomura, T. Tagami, S. Yamazaki, T. Sakihama, T. Matsutani, I. Negishi, S. Nakatsuru, and S. Sakaguchi. 2003. Altered thymic T-cell selection due to a mutation of the ZAP-70 gene causes autoimmune arthritis in mice. Nature. 426:454–460. 10.1038/nature02119 14647385

[bib81] Sakaguchi, S., H. Benham, A.P. Cope, and R. Thomas. 2012. T-cell receptor signaling and the pathogenesis of autoimmune arthritis: Insights from mouse and man. Immunol. Cell Biol. 90:277–287. 10.1038/icb.2012.4 22418389

[bib82] Schim van der Loeff, I., L.Y. Hsu, M. Saini, A. Weiss, and B. Seddon. 2014. Zap70 is essential for long-term survival of naive CD8 T cells. J. Immunol. 193:2873–2880. 10.4049/jimmunol.1400858 25092893 PMC4167606

[bib83] Seddon, B., G. Legname, P. Tomlinson, and R. Zamoyska. 2000. Long-term survival but impaired homeostatic proliferation of Naïve T cells in the absence of p56lck. Science. 290:127–131. 10.1126/science.290.5489.127 11021796

[bib84] Sellon, R.K., S. Tonkonogy, M. Schultz, L.A. Dieleman, W. Grenther, E. Balish, D.M. Rennick, and R.B. Sartor. 1998. Resident enteric bacteria are necessary for development of spontaneous colitis and immune system activation in interleukin-10-deficient mice. Infect. Immun. 66:5224–5231. 10.1128/IAI.66.11.5224-5231.1998 9784526 PMC108652

[bib85] Serwas, N.K., D. Cagdas, S.A. Ban, K. Bienemann, E. Salzer, I. Tezcan, A. Borkhardt, O. Sanal, and K. Boztug. 2014. Identification of ITK deficiency as a novel genetic cause of idiopathic CD4^+^ T-cell lymphopenia. Blood. 124:655–657. 10.1182/blood-2014-03-564930 25061172 PMC4110665

[bib86] Shearer, W.T., H.M. Rosenblatt, R.S. Gelman, R. Oyomopito, S. Plaeger, E.R. Stiehm, D.W. Wara, S.D. Douglas, K. Luzuriaga, E.J. McFarland, . 2003. Lymphocyte subsets in healthy children from birth through 18 years of age: The pediatric AIDS clinical trials group P1009 study. J. Allergy Clin. Immunol. 112:973–980. 10.1016/j.jaci.2003.07.003 14610491

[bib87] Siggs, O.M., L.A. Miosge, A.L. Yates, E.M. Kucharska, D. Sheahan, T. Brdicka, A. Weiss, A. Liston, and C.C. Goodnow. 2007. Opposing functions of the T cell receptor kinase ZAP-70 in immunity and tolerance differentially titrate in response to nucleotide substitutions. Immunity. 27:912–926. 10.1016/j.immuni.2007.11.013 18093540 PMC3163119

[bib88] Sommers, C.L., C.S. Park, J. Lee, C. Feng, C.L. Fuller, A. Grinberg, J.A. Hildebrand, E. Lacaná, R.K. Menon, E.W. Shores, . 2002. A LAT mutation that inhibits T cell development yet induces lymphoproliferation. Science. 296:2040–2043. 10.1126/science.1069066 12065840

[bib89] Stepankova, R., F. Powrie, O. Kofronova, H. Kozakova, T. Hudcovic, T. Hrncir, H. Uhlig, S. Read, Z. Rehakova, O. Benada, . 2007. Segmented filamentous bacteria in a defined bacterial cocktail induce intestinal inflammation in SCID mice reconstituted with CD45RBhigh CD4^+^ T cells. Inflamm. Bowel Dis. 13:1202–1211. 10.1002/ibd.20221 17607724

[bib90] Takahashi, T., Y. Kuniyasu, M. Toda, N. Sakaguchi, M. Itoh, M. Iwata, J. Shimizu, and S. Sakaguchi. 1998. Immunologic self-tolerance maintained by CD25^+^CD4^+^ naturally anergic and suppressive T cells: Induction of autoimmune disease by breaking their anergic/suppressive state. Int. Immunol. 10:1969–1980. 10.1093/intimm/10.12.1969 9885918

[bib91] Tanaka, S., S. Maeda, M. Hashimoto, C. Fujimori, Y. Ito, S. Teradaira, K. Hirota, H. Yoshitomi, T. Katakai, A. Shimizu, . 2010. Graded attenuation of TCR signaling elicits distinct autoimmune diseases by altering thymic T cell selection and regulatory T cell function. J. Immunol. 185:2295–2305. 10.4049/jimmunol.1000848 20644168

[bib92] Tang, Q., K.J. Henriksen, E.K. Boden, A.J. Tooley, J. Ye, S.K. Subudhi, X.X. Zheng, T.B. Strom, and J.A. Bluestone. 2003. Cutting edge: CD28 controls peripheral homeostasis of CD4^+^CD25^+^ regulatory T cells. J. Immunol. 171:3348–3352. 10.4049/jimmunol.171.7.3348 14500627

[bib93] Tarakhovsky, A., S.B. Kanner, J. Hombach, J.A. Ledbetter, W. Müller, N. Killeen, and K. Rajewsky. 1995. A role for CD5 in TCR-mediated signal transduction and thymocyte selection. Science. 269:535–537. 10.1126/science.7542801 7542801

[bib94] Tokgoz, H., U. Caliskan, S. Keles, I. Reisli, I.S. Guiu, and N.V. Morgan. 2013. Variable presentation of primary immune deficiency: Two cases with CD3 gamma deficiency presenting with only autoimmunity. Pediatr. Allergy Immunol. 24:257–262. 10.1111/pai.12063 23590417

[bib95] Trobridge, P.A., K.A. Forbush, and S.D. Levin. 2001. Positive and negative selection of thymocytes depends on Lck interaction with the CD4 and CD8 coreceptors. J. Immunol. 166:809–818. 10.4049/jimmunol.166.2.809 11145654

[bib96] Turner, P.V. 2018. The role of the gut microbiota on animal model reproducibility. Anim. Model. Exp. Med. 1:109–115. 10.1002/ame2.12022 PMC6388061 30891555

[bib97] van Oers, N.S., N. Killeen, and A. Weiss. 1996a. Lck regulates the tyrosine phosphorylation of the T cell receptor subunits and ZAP-70 in murine thymocytes. J. Exp. Med. 183:1053–1062. 10.1084/jem.183.3.1053 8642247 PMC2192313

[bib98] van Oers, N.S., B. Lowin-Kropf, D. Finlay, K. Connolly, and A. Weiss. 1996b. alpha beta T cell development is abolished in mice lacking both Lck and Fyn protein tyrosine kinases. Immunity. 5:429–436. 10.1016/S1074-7613(00)80499-9 8934570

[bib99] Varma, R., G. Campi, T. Yokosuka, T. Saito, and M.L. Dustin. 2006. T cell receptor-proximal signals are sustained in peripheral microclusters and terminated in the central supramolecular activation cluster. Immunity. 25:117–127. 10.1016/j.immuni.2006.04.010 16860761 PMC1626533

[bib100] Veillette, A., M.A. Bookman, E.M. Horak, and J.B. Bolen. 1988. The CD4 and CD8 T cell surface antigens are associated with the internal membrane tyrosine-protein kinase p56lck. Cell. 55:301–308. 10.1016/0092-8674(88)90053-0 3262426

[bib101] Yamaguchi, H., and W.A. Hendrickson. 1996. Structural basis for activation of human lymphocyte kinase Lck upon tyrosine phosphorylation. Nature. 384:484–489. 10.1038/384484a0 8945479

[bib102] Zhu, X., J.L. Kim, J.R. Newcomb, P.E. Rose, D.R. Stover, L.M. Toledo, H. Zhao, and K.A. Morgenstern. 1999. Structural analysis of the lymphocyte-specific kinase Lck in complex with non-selective and Src family selective kinase inhibitors. Structure. 7:651–661. 10.1016/S0969-2126(99)80086-0 10404594

[bib103] Zunder, E.R., R. Finck, G.K. Behbehani, E.A.D. Amir, S. Krishnaswamy, V.D. Gonzalez, C.G. Lorang, Z. Bjornson, M.H. Spitzer, B. Bodenmiller, . 2015. Palladium-based mass tag cell barcoding with a doublet-filtering scheme and single-cell deconvolution algorithm. Nat. Protoc. 10:316–333. 10.1038/nprot.2015.020 25612231 PMC4347881

