# Supplementary material for: A partial human LCK defect causes a T cell immunodeficiency with intestinal inflammation
Source: J Exp Med. 2023 Nov 14;221(1):e20230927. doi: 10.1084/jem.20230927 (PMC10644909; doi:10.1084/jem.20230927)
Supplement: Table S1 — shows details of HSCT. [file JEM_20230927_TableS1.docx]

**Table S1: Details of HSCT**

| **Patient** | **P1** | **P2** |
| --- | --- | --- |
| Age at HSCT (yrs) | 6.7 | 3.0 |
| Donor | MMFD (Mother) | MMFD (Mother) |
| HLA match | 5/10 | 5/10 |
| Stem cell source,  graft manipulation | BM, unmanipulated | BM, unmanipulated |
| Chemotherapy | Treo 42 (d-6 to d-4)  Flu 160 (d-7 to d-4)  Cy 29 (d-3 to d-2) | Treo 36 (d-7 to d-5)  Flu 160 (d-8 to d-5)  Cy 29 (d-4 to d-3) |
| Serotherapy | Alemtuzumab 0.7mg/kg (d-12 to d-9)  Rituximab 375mg/m^2^ (d-16) | Alemtuzumab 0.7mg/kg (d-12 to d-9)  Rituximab 375mg/m^2^ (d-14) |
| GvHD prophylaxis | MMF, Tacrolimus, Cyclophosphamide (d+3/+4) | MMF, Tacrolimus, Cyclophosphamide (d+3/+4) |
| Engraftment | N+21/ E-/ P- | N+34/ E+20/ P+23 |
| Secondary procedures, reason, donor, graft, days post HSCT | Boost, poor graft function, mother, CD34-positive, d+56 | Boost, poor graft function, mother, CD34-positive, d+154 |
| Chimerism  [% donor] | Mixed (CD3+ 100%, CD3- 87%, CD15+ 63% - day+94) | Mixed (CD3+ 100%, CD3- 24%, CD15+ 29% - day+251) |
| aGvHD | no | skin 2 (overall grade 1) |
| cGvHD | no | no |
| Post Tx follow-up | 0.3 yrs | 0.7 yrs |
| Outcome | Deceased, pneumonia with subsequent multiorgan failure (niai) | Alive and well  On tuberculostatic therapy since d+135 until 6 months after cessation of immunosuppressive prophylaxis  (triple therapy with rifampicin, isoniazid, ethambutol – continued without ethambulole after regression of lymphadenitis on d+176) |

**Abbreviations:** d: day; yrs: years; niai: no infectious agent identified; MMFD: mismatched family donor; BM: bone marrow; Cy: cyclophosphamide dosage given in mg/kg; Flu: fludarabine dosage given in mg/m^2^; Treo: treosulfan dosage given in g/m^2^; MMF: mycophenolate mofetil; N: neutrophil engraftment, first of three consecutive days with >500/µl; E: red cell engraftment, first of three consecutive days with reticulocyte counts of >20%o; P: platelet engraftment, first of three consecutive days with >20,000/µl.
