# Supplementary material for: A partial human LCK defect causes a T cell immunodeficiency with intestinal inflammation
Source: J Exp Med. 2023 Nov 14;221(1):e20230927. doi: 10.1084/jem.20230927 (PMC10644909; doi:10.1084/jem.20230927)
Supplement: Table S2 — shows chimerism and immunological reconstitution after transplantation. [file JEM_20230927_TableS2.docx]

**Table S2: Chimerism and immunological reconstitution after HSCT**

| **Follow up details** | | **P1** | | **P2** | |
| --- | --- | --- | --- | --- | --- |
| Days after HSCT | | +94 | | +251 | |
|  | **Chimerism** | | | | |
| T cells | % donor | 100 | | 100 | |
| Non-T-cells (CD3- MNCs) | % donor | 87 | | 24 | |
| Neutrophils (CD15+) | % donor | 63 | | 29 | |
|  | **Immunological reconstitution** | | | | |
|  |  |  | *Ref.values^1^* |  | *Ref.values^2^* |
| Lymphocytes | cells/µl | 630 | *1500-4200* | 630 | *1700-5700* |
| T cells | cells/µl | 220 | *1000-3300* | 536 | *1200-3900* |
| B cells | cells/µl | 0 | *210- 800* | 0 | *310-1600* |
| NK cells | cells/µl | 397 | *80- 600* | 94 | *110- 600* |
| T cells naïve  (% of CD45RA+CCR7+ of CD4+) | % | 0 | *49- 78* | 8 | *51- 81* |
| IVIG |  | yes |  | yes |  |
| Specific antibodies |  | n.d. |  | n.d. |  |

*Age-matched reference values (p5-p95) determined in healthy children ^1^6-12 yrs n=64; ^2^2-6 yrs n=40 (Ulm, dept. of pediatrics, immunology lab, unpublished data)*

**Abbreviations:** yrs: years; n.d.: not determined; IVIG: intravenous substitution of IgG
