# Supplementary material for: A partial human LCK defect causes a T cell immunodeficiency with intestinal inflammation
Source: J Exp Med. 2023 Nov 14;221(1):e20230927. doi: 10.1084/jem.20230927 (PMC10644909; doi:10.1084/jem.20230927)
Supplement: Table S3 — shows whole-exome sequencing variants. [file JEM_20230927_TableS3.docx]

**Table S3. Whole exome sequencing variants**

| **Gene** | **Refseq** | **Chr** | **Nucleotide change** | | **AA change** | **Effect** | **Disease inheritance** | **Polyphen** |  | **SIFT** |
| --- | --- | --- | --- | --- | --- | --- | --- | --- | --- | --- |
| LCK | NM_005356.3 | 1 | C>T | P440>S | | Missense | auto-recessive | 0.973 |  | 0 |
| IDS | NM_000202.5 | X | A>C | L10>R | | Missense | X-recessive | 0.612 |  | 0.21 |
