# Supplementary material for: A partial human LCK defect causes a T cell immunodeficiency with intestinal inflammation
Source: J Exp Med. 2023 Nov 14;221(1):e20230927. doi: 10.1084/jem.20230927 (PMC10644909; doi:10.1084/jem.20230927)
Supplement: Table S4 — shows the features of the LCK P440S variant. [file JEM_20230927_TableS4.docx]

**Table S4: Features of the LCK P440S variant**

| Variant annotation | |
| --- | --- |
| Chromosome | 1 |
| Genomic position (GRCh37/hg19) | 32745802 |
| cDNA position (NM_001042771.3) | 1318 |
| Nucleotide reference | C |
| Nucleotide variant | T |
| Protein variant (NP_001036236.1) | Pro440Ser; P440S |
| Zygosity in patients | Homozygous |
| dbSNP155 | rs1318678254 |
| gnomAD (v3.1.2) | 1 Heterozygous; No Homozygous; Allele Frequency 6.57e-6 |
| COSMIC | Somatic Report; 2 Entries (Mutation ID COSM3488147) |
| ClinVar | No Entry |
| *In silico* pathogenicity prediction models | |
| CADD | 26.1 |
| SIFT | Deleterious (0) |
| Polyphen2 | Damaging (1) |
| LRT | Deleterious (0) |
| Mutation Taster | Disease Causing (0.81) |
| PROVEAN | Deleterious (-7.1) |
| MetaSVM | Deleterious (0.98) |
| M-CAP | Possibly Pathogenic (0.37) |
| fathmm-MKL | Deleterious (1) |
