# Supplementary material for: A partial human LCK defect causes a T cell immunodeficiency with intestinal inflammation
Source: J Exp Med. 2023 Nov 14;221(1):e20230927. doi: 10.1084/jem.20230927 (PMC10644909; doi:10.1084/jem.20230927)
Supplement: Table S5 — shows the mass cytometry antibody staining panel. [file JEM_20230927_TableS5.docx]

**Table S5. Mass cytometry antibody staining panel**

| **Antibody** | **Clone** | **Metal** | **Channel** | **Titer (Vol/rxn)** | **Manufacturer** |
| --- | --- | --- | --- | --- | --- |
| CD45 | HI30 | Y | 89 | 0.3 | Fluidigm 3089003B |
| CD57 | HCD57 | In | 113 | 1 | Biolegend 322302 |
| CD11c | B-ly6 | In | 115 | 1.5 | BD 555390 |
| CD21 | Bu32 | Pr | 141 | 1 | Biolegend 354902 |
| CD8 | SK1 | Nd | 142 | 0.25 | Biolegend 344702 |
| CD3 | HIT3a | Nd | 144 | 0.3 | BD 555337 |
| CD45RA | HI100 | Nd | 145 | 0.3 | Biolegend 304102 |
| IgD | IA6-2 | Nd | 146 | 1.2 | Fluidigm 3146005B |
| CD38 | HIT2 | Nd | 148 | 1 | Biolegend 303502 |
| CXCR5 | RF8B2 | Nd | 150 | 0.25 | BD 552032 |
| IgM | MHM-88 | Eu | 151 | 1.2 | Biolegend 314527 |
| CD141 | M80 | Sm | 152 | 1 | Biolegend 344102 |
| CD16 | B73.1 | Eu | 153 | 1 | eBioscience 16-0167-85 |
| CD14 | M5E2 | Sm | 154 | 1 | Biolegend 301802 |
| CD4 | SK3 | Gd | 155 | 1 | Biolegend 344602 |
| CD7 | M-T701 | Gd | 156 | 2 | BD 555359 |
| PD1 | EH12.2H7 | In | 157 | 0.75 | Biolegend 329902 |
| CD1c | L161 | Dy | 161 | 1 | Biolegend 331502 |
| CD27 | L128 | Yb | 162 | 1 | Fluidigm 3162009B |
| CD19 | SJ25C1 | Dy | 163 | 1 | Biolegend 363002 |
| CD123 | 9F5 | Dy | 164 | 1.5 | BD 555642 |
| ICOS | C398.4A | Er | 166 | 0.75 | Biolegend 313502 |
| CD25 | M-A251 | Er | 167 | 0.5 | Biolegend 356102 |
| CD56 | REA196 | ER | 168 | 0.3 | Miltenyi Biotech  130-108-016 |
| HLADR | L243 | Er | 179 | 1 | Fluidigm 3170013B |
| CTLA4 | BNI3 | Nd | 143 | 2 | BD 555851 |
| pp90RSK (pSer380) | D5D8 | Sm | 147 | 1 | Cell Signaling 9341 |
| p4E-BP1 (pThr37/  pThr46) | 236B4 | Sm | 149 | 1 | Fluidigm  3149005A |
| pPLC𝛄2 (pTyr759) | K86-689.37 | Gd | 158 | 1 | BD (custom) |
| pBTK (pTyr223)/  pITK (pTyr180) | N35-86 | Tb | 159 | 0.15 | BD (custom) |
| IKBa | L35A5 | Gd | 160 | 2 | Cell Signaling 4814BF |
| pCREB (pSer133) | 87G3 | Ho | 165 | 0.1 | Cell Signaling 9198 |
| pERK1/2 (pThr202/  pThr204) | MILAN8R | Nd | 169 | 1.5 | eBioscience  14-9109-0 |
| pZAP70 (pTyr319)/  pSYK (pTyr352) | 17A/P-ZAP70 | Yb | 171 | 3 | BD 612575 |
| pSLP76 (pTyr128) | J141-668.36.58 | Yb | 172 | 0.5 | BD 558367 |
| pCD3𝜁 (pTyr142) | K25-407.69 | Nd | 173 | 1.5 | BD 558402 |
| pAKT (pSer473) | M89-61 | Yb | 174 | 3 | BD 560397 |
| pS6 (pSer235/  pSer236) | N7-548 | Lu | 175 | 1 | Fluidigm  3175009A |
| pLCK (pTyr394) | 755103 | Yb | 176 | 2 | R&D MAB7500 |
