# Supplementary figures and images for: A partial human LCK defect causes a T cell immunodeficiency with intestinal inflammation
Source: J Exp Med. 2023 Nov 14;221(1):e20230927. doi: 10.1084/jem.20230927 (PMC10644909; doi:10.1084/jem.20230927)

Figure 1F

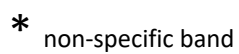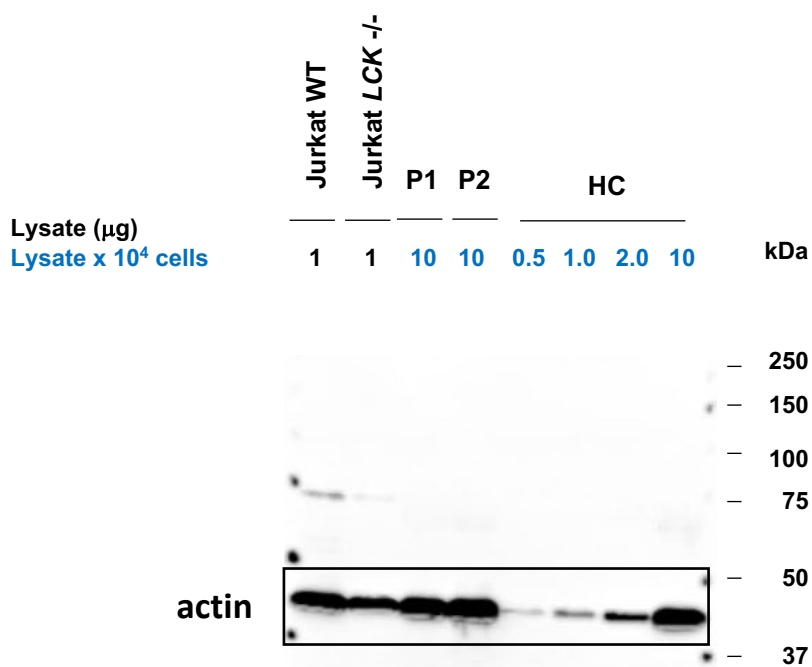

Supplement: SourceData F1 — contains original blots for Fig. 1. [file JEM_20230927_SourceDataF1.pdf]
