# Supplementary material for: A partial human LCK defect causes a T cell immunodeficiency with intestinal inflammation
Source: J Exp Med. 2023 Nov 14;221(1):e20230927. doi: 10.1084/jem.20230927 (PMC10644909; doi:10.1084/jem.20230927)
Supplement: SourceData F2 — contains original blots for Fig. 2. [file JEM_20230927_SourceDataF2.pdf]

Figure 2C

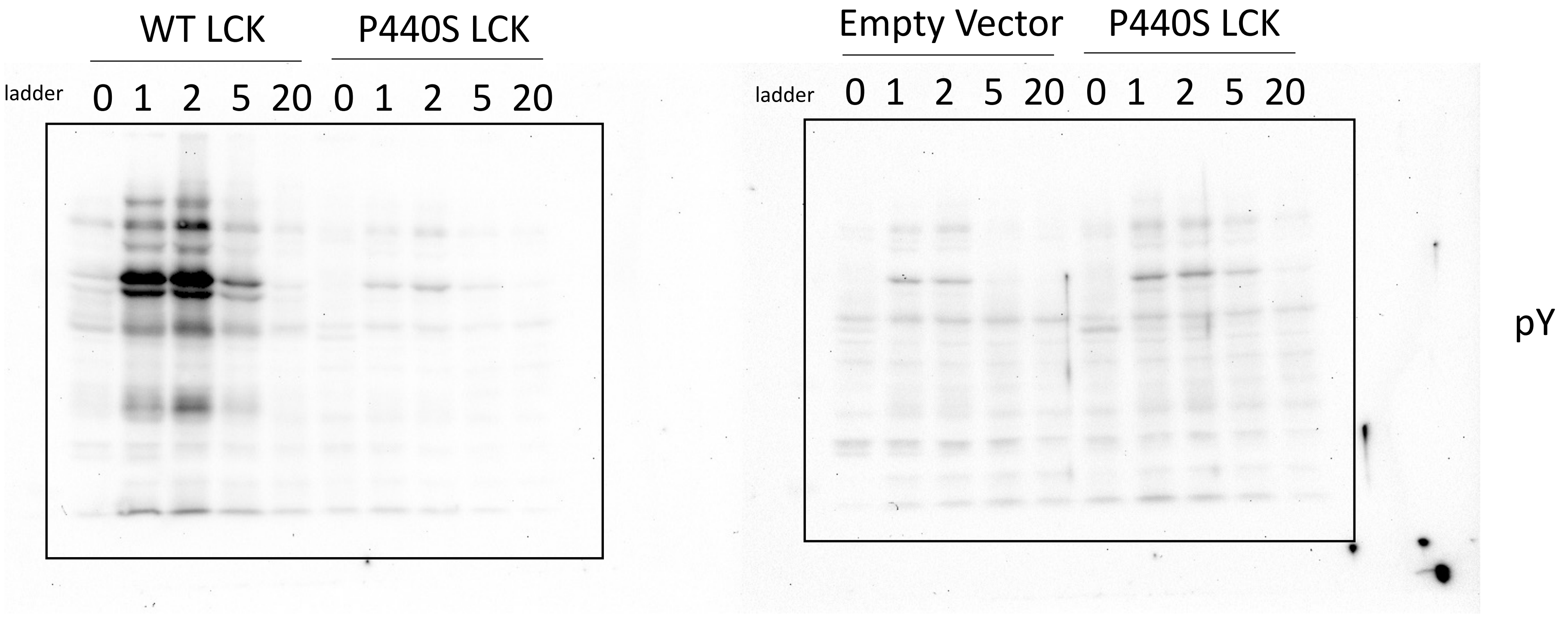
WT LCK
P440S LCK
ladder
0 1 2 5 20 0 1 2 5 20
Empty Vector
P440S LCK
ladder
0 1 2 5 20 0 1 2 5 20
pY

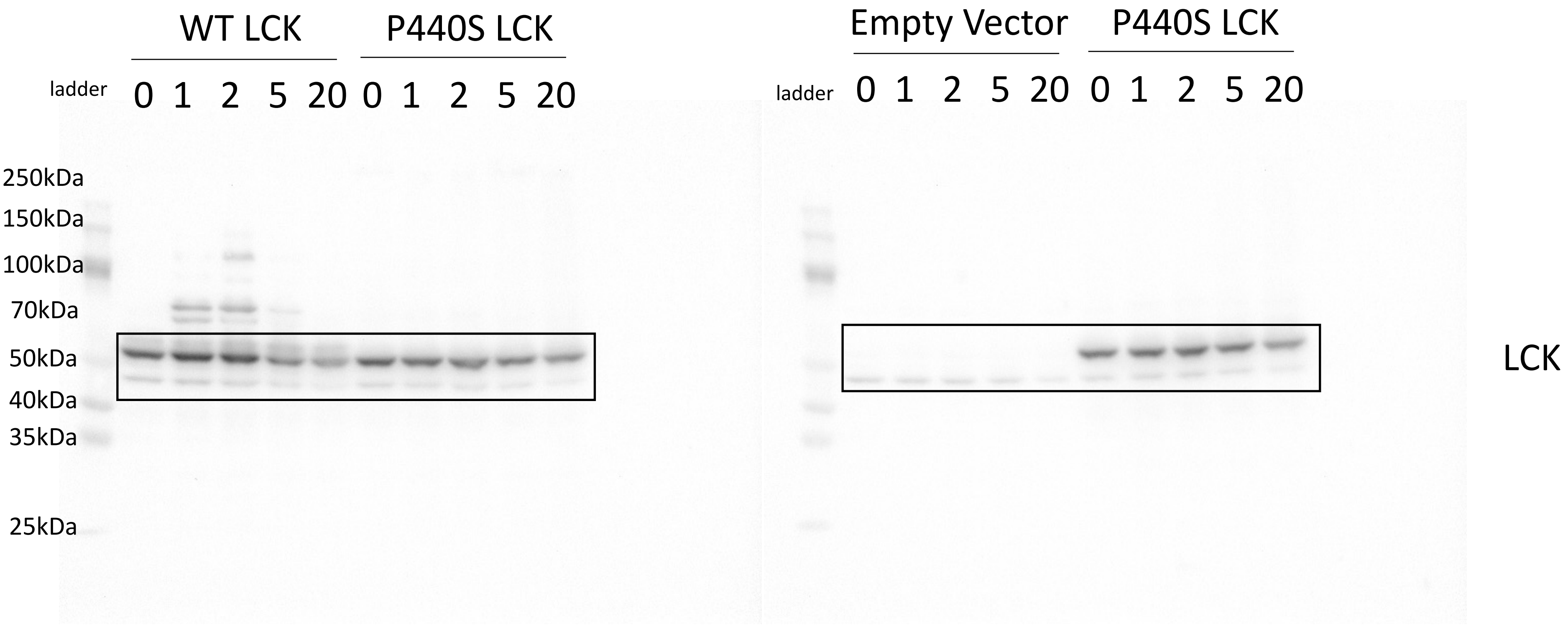
WT LCK
P440S LCK
ladder
0 1 2 5 20 0 1 2 5 20
250kDa
150kDa
100kDa
70kDa
50kDa
40kDa
35kDa
25kDa
Empty Vector
P440S LCK
ladder
0 1 2 5 20 0 1 2 5 20
LCK

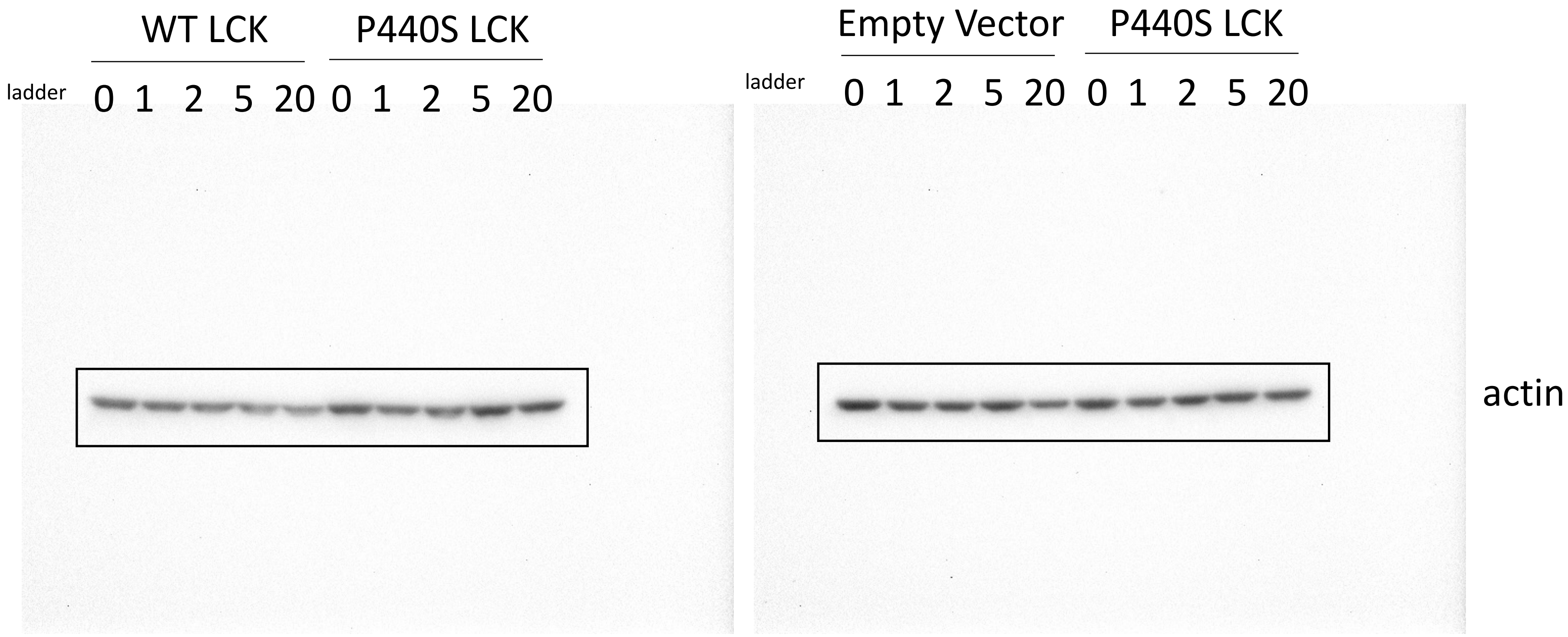
WT LCK
P440S LCK
ladder
0 1 2 5 20 0 1 2 5 20
Empty Vector
P440S LCK
ladder
0 1 2 5 20 0 1 2 5 20
actin
