# Supplementary material for: A partial human LCK defect causes a T cell immunodeficiency with intestinal inflammation
Source: J Exp Med. 2023 Nov 14;221(1):e20230927. doi: 10.1084/jem.20230927 (PMC10644909; doi:10.1084/jem.20230927)

Figure S2A

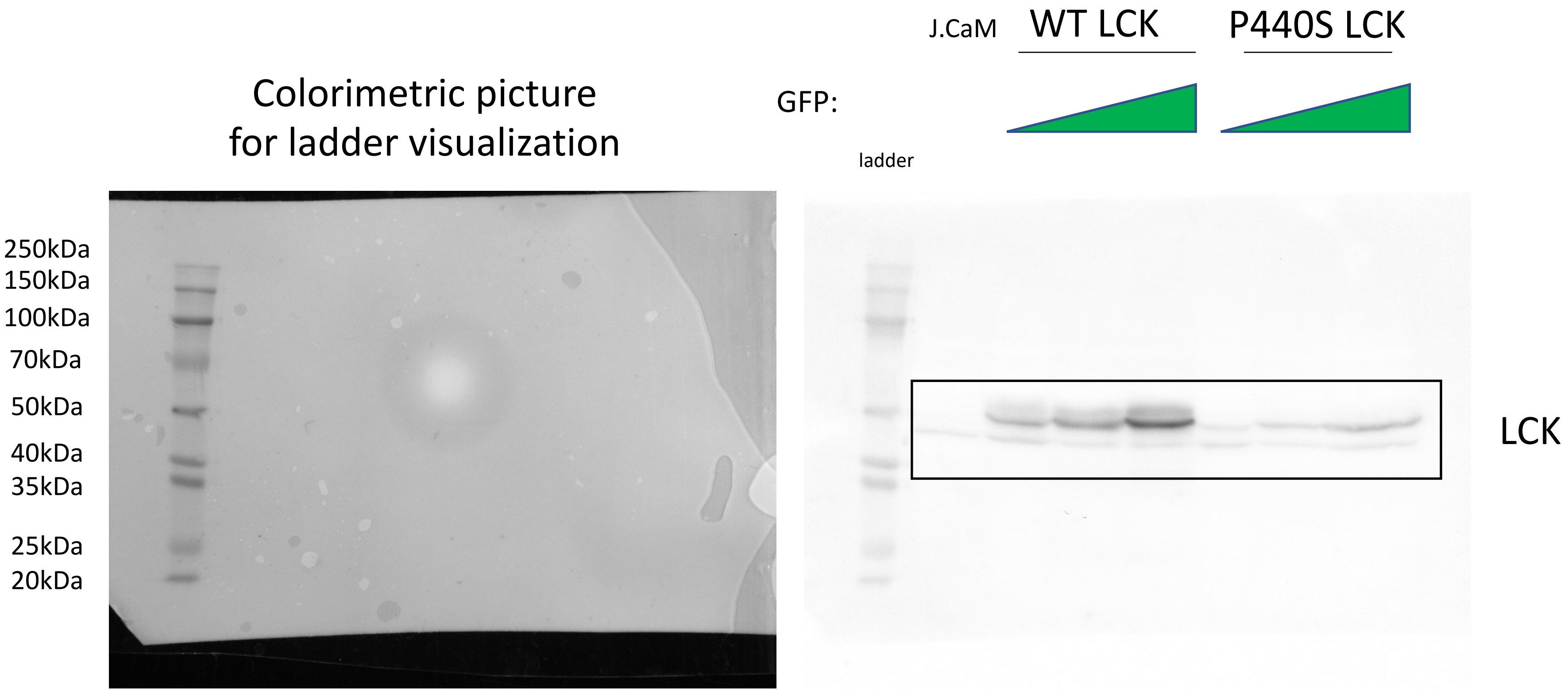
Colorimetric picture
for ladder visualization
J.CaM
WT LCK
P440S LCK
GFP:
ladder
250kDa
150kDa
100kDa
70kDa
50kDa
40kDa
35kDa
25kDa
20kDa
LCK

LCK MW=56kD

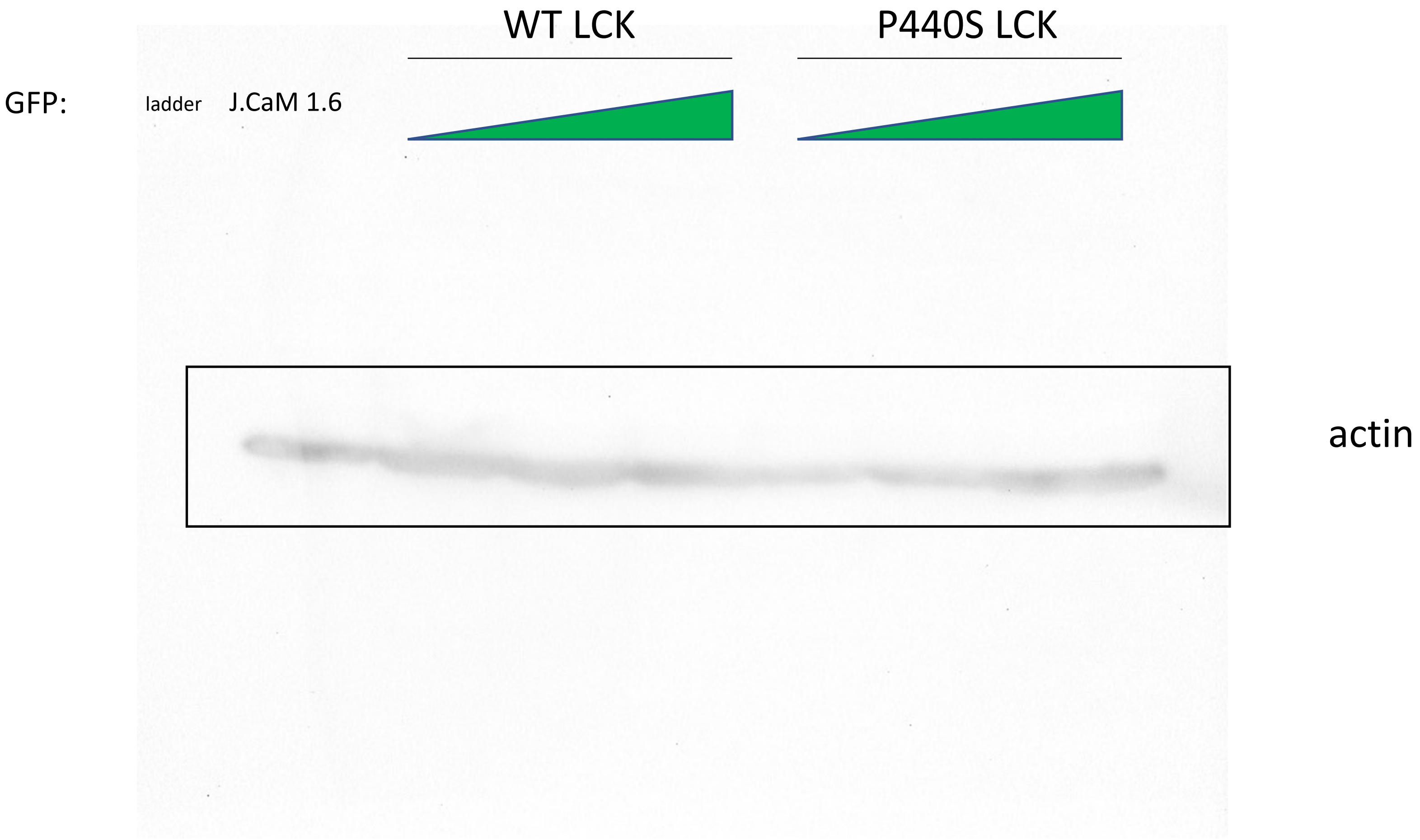
WT LCK
P440S LCK
GFP:
ladder
J.CaM 1.6
actin

Figure S2B

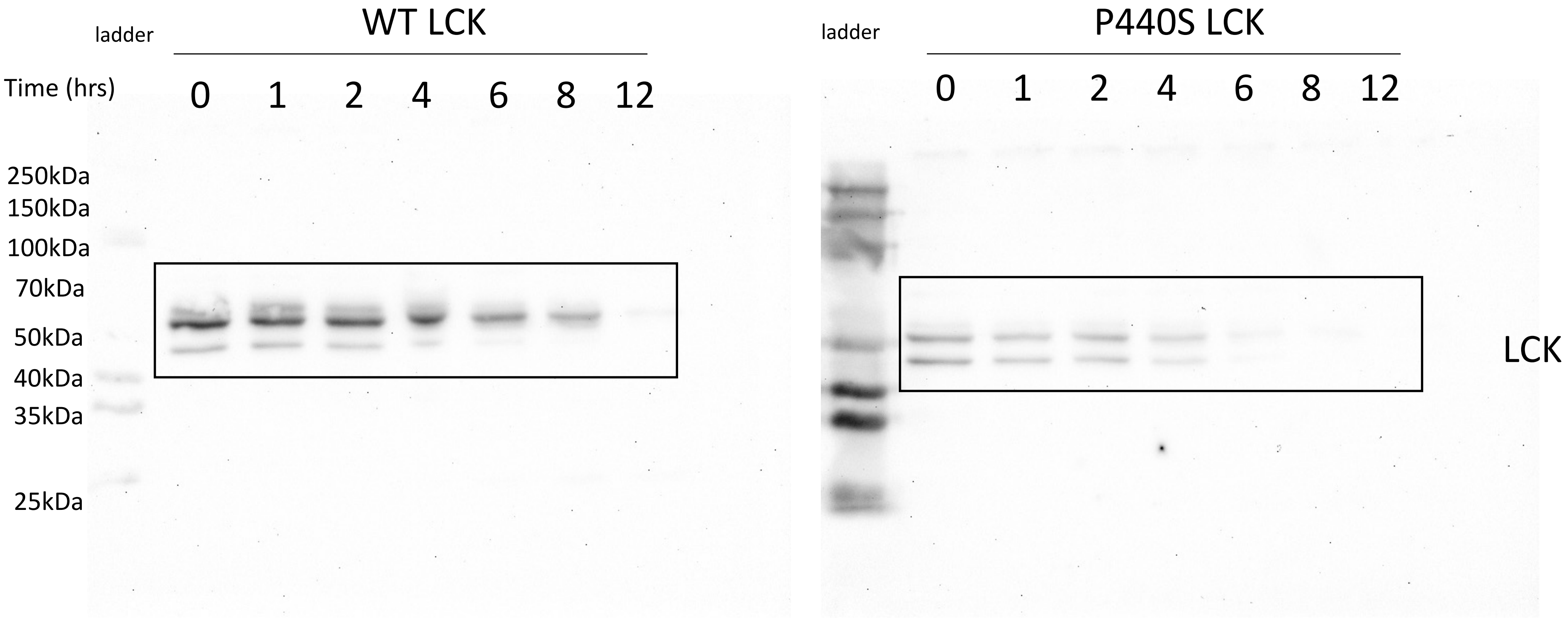

ladder
WT LCK
Time (hrs)
0
1
2
4
6
8
12
250kDa
150kDa
100kDa
70kDa
50kDa
40kDa
35kDa
25kDa
ladder
P440S LCK
0
1
2
4
6
8
12
LCK

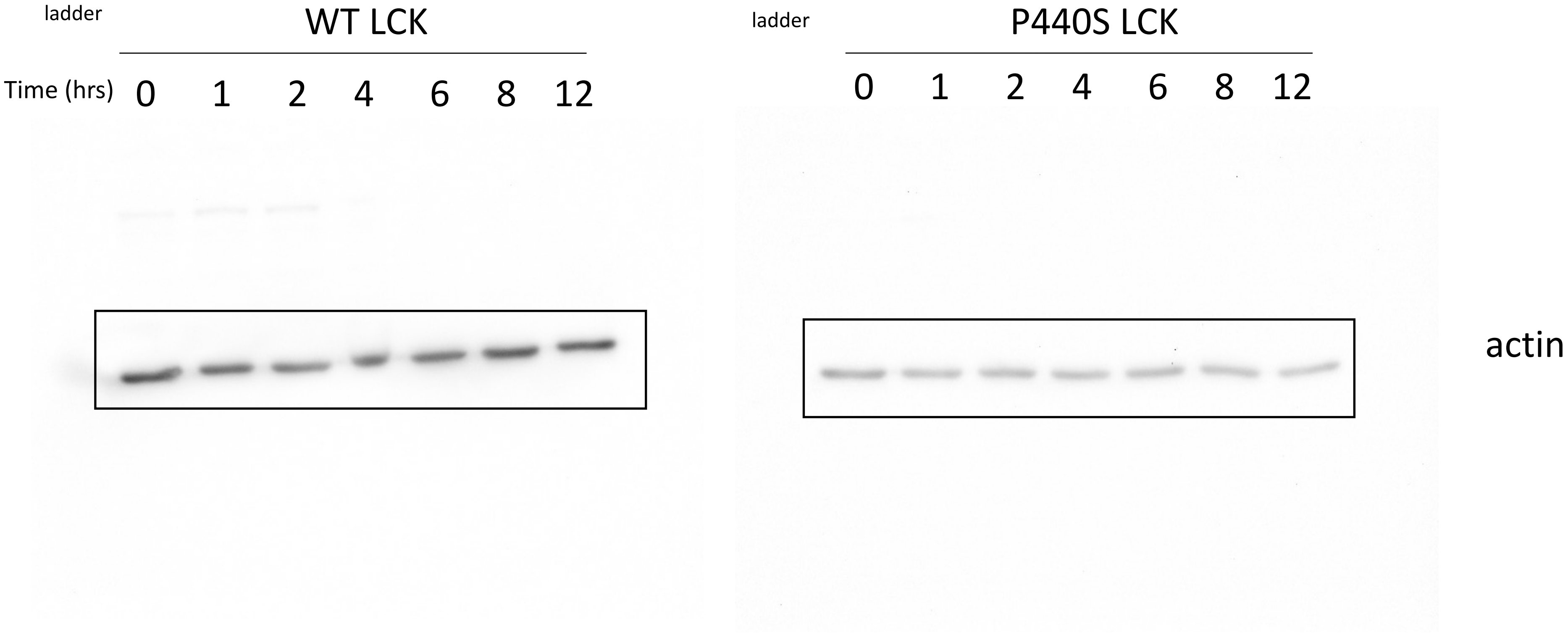

ladder
WT LCK
Time (hrs) 0 1 2 4 6 8 12
ladder
P440S LCK
0 1 2 4 6 8 12
actin

Figure S2C

Colorimetric picture for ladder visualization

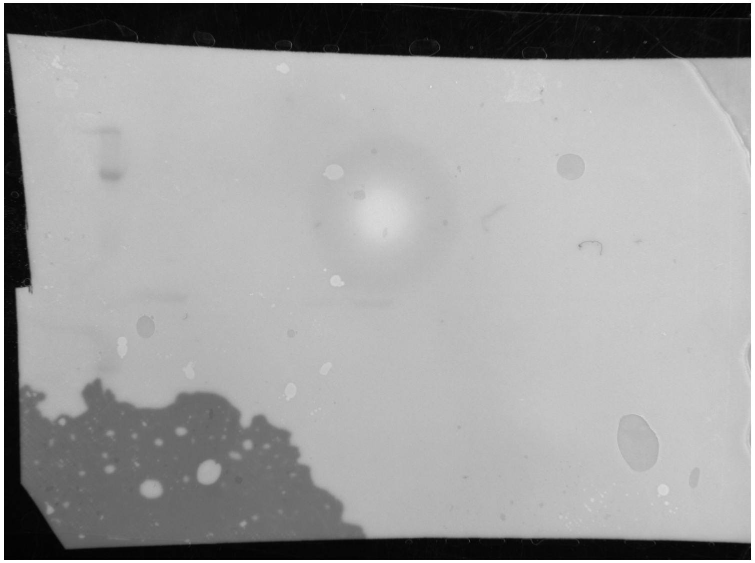

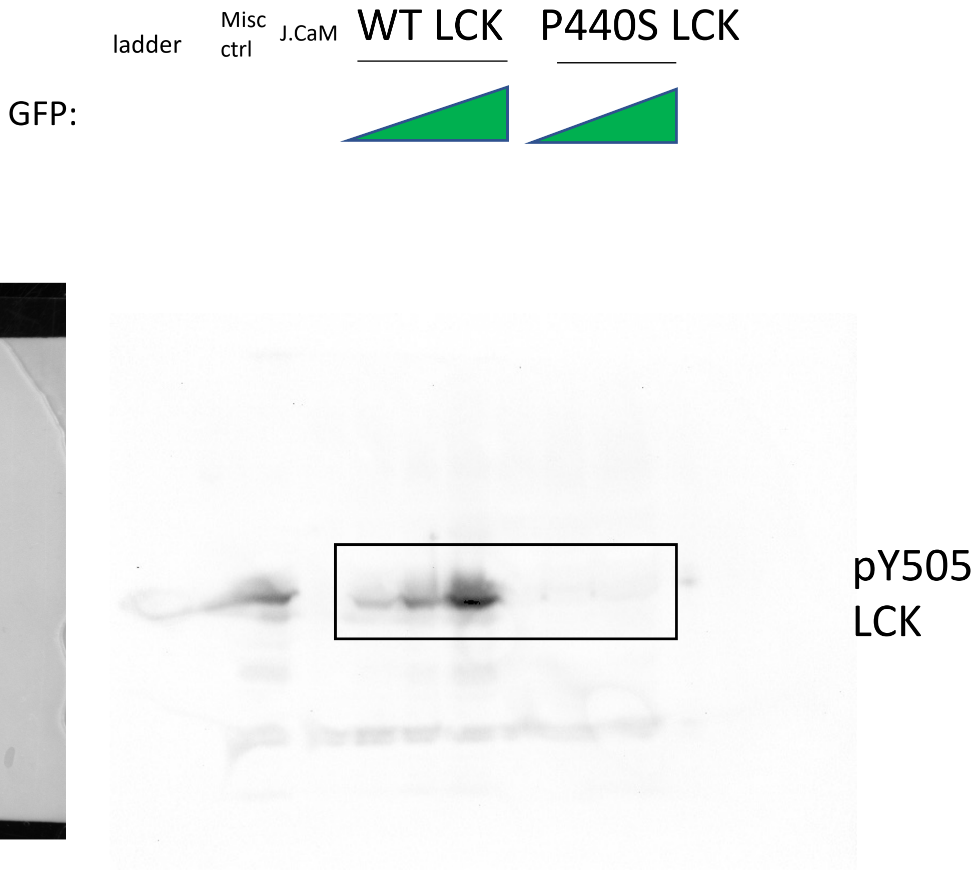

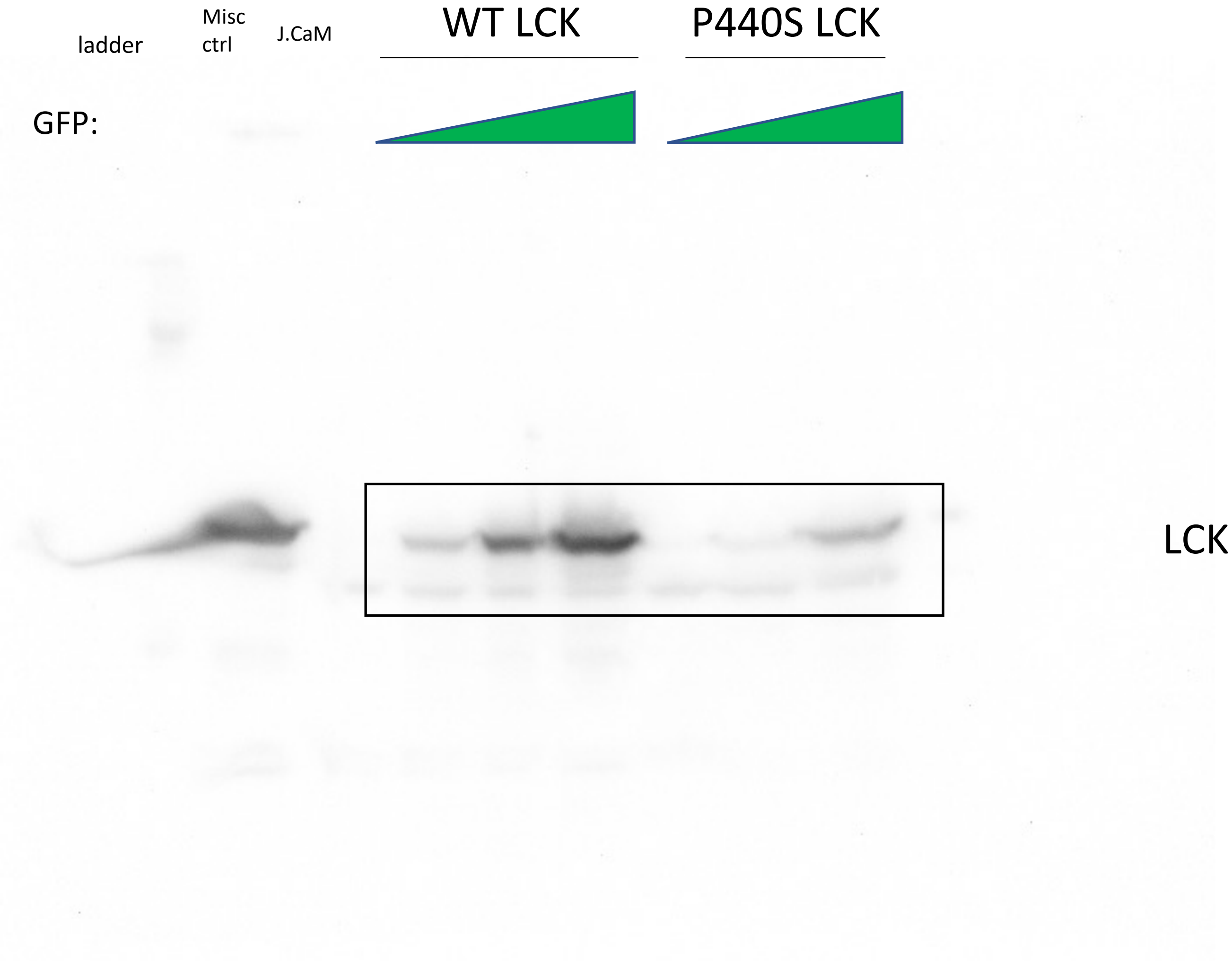
ladder
Misc ctrl
J.CaM
WT LCK
P440S LCK
GFP:
LCK

Figure S2D

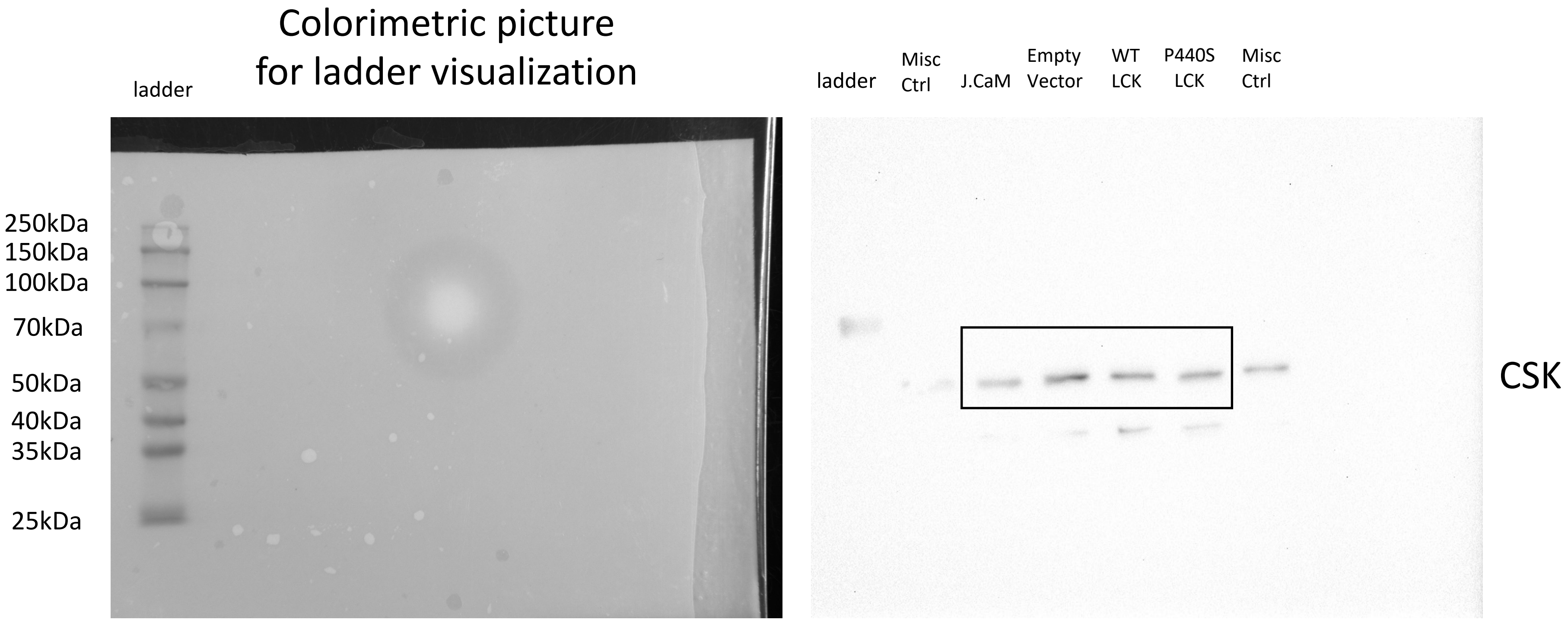

Colorimetric picture
for ladder visualization
ladder
250kDa
150kDa
100kDa
70kDa
50kDa
40kDa
35kDa
25kDa
ladder
Misc Ctrl
J.CaM
Empty Vector
WT LCK
P440S LCK
Misc Ctrl
CSK

CSK MW=50kD

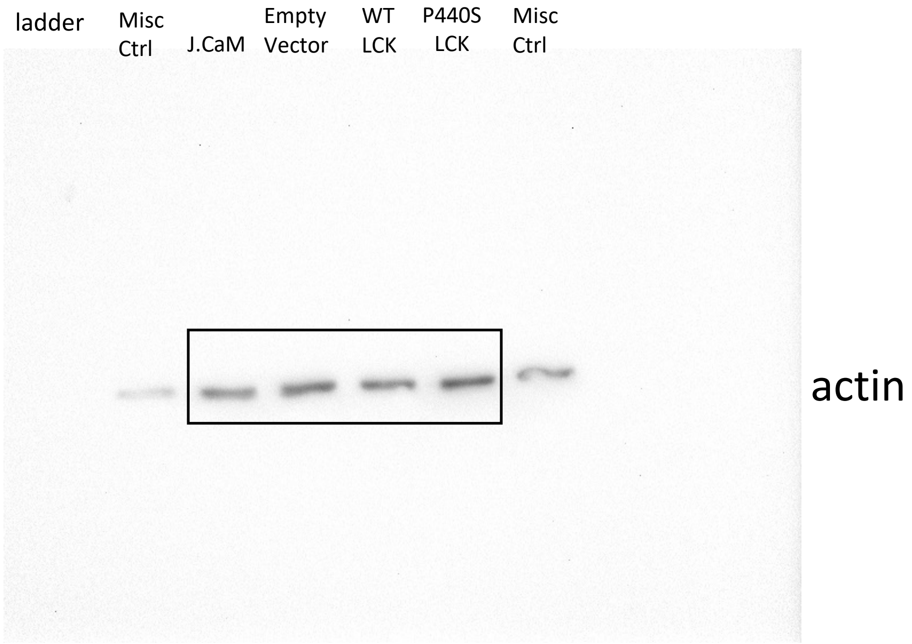
ladder
Misc Ctrl
J.CaM
Empty Vector
WT LCK
P440S LCK
Misc Ctrl
actin

Figure S2E

Colorimetric picture
for ladder visualization

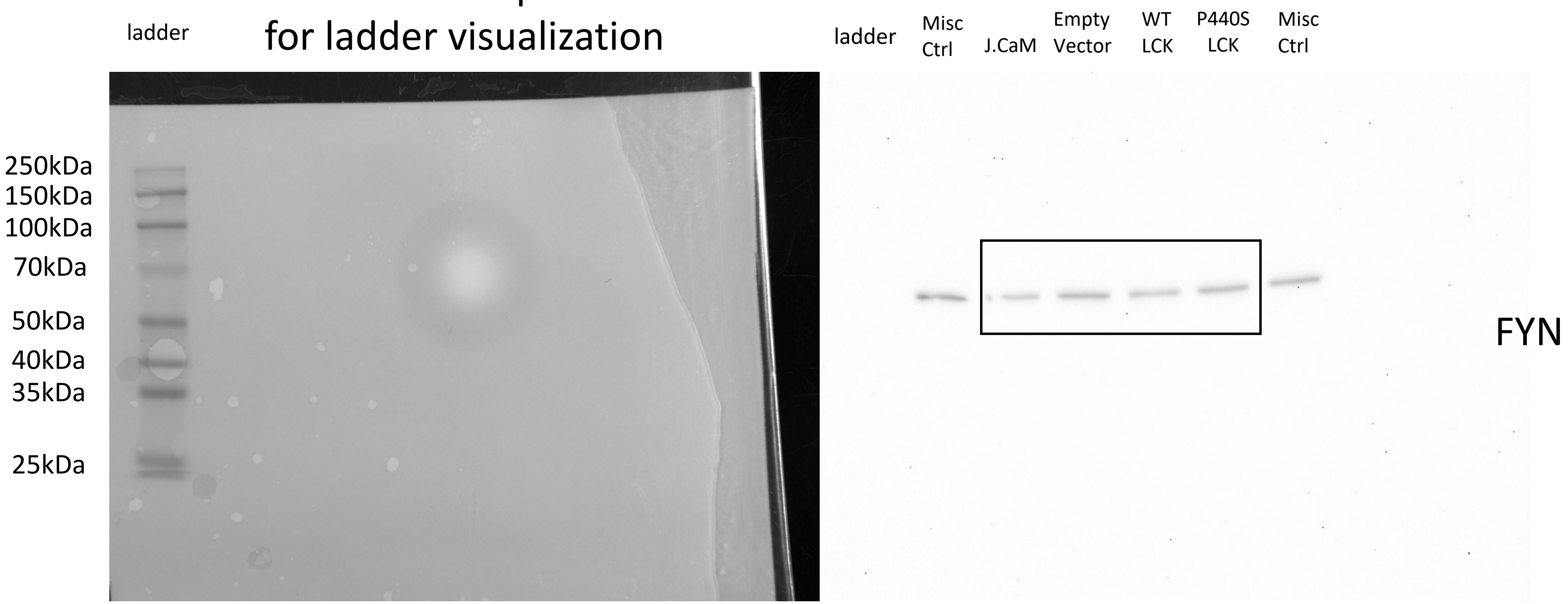

FYN MW=59kD

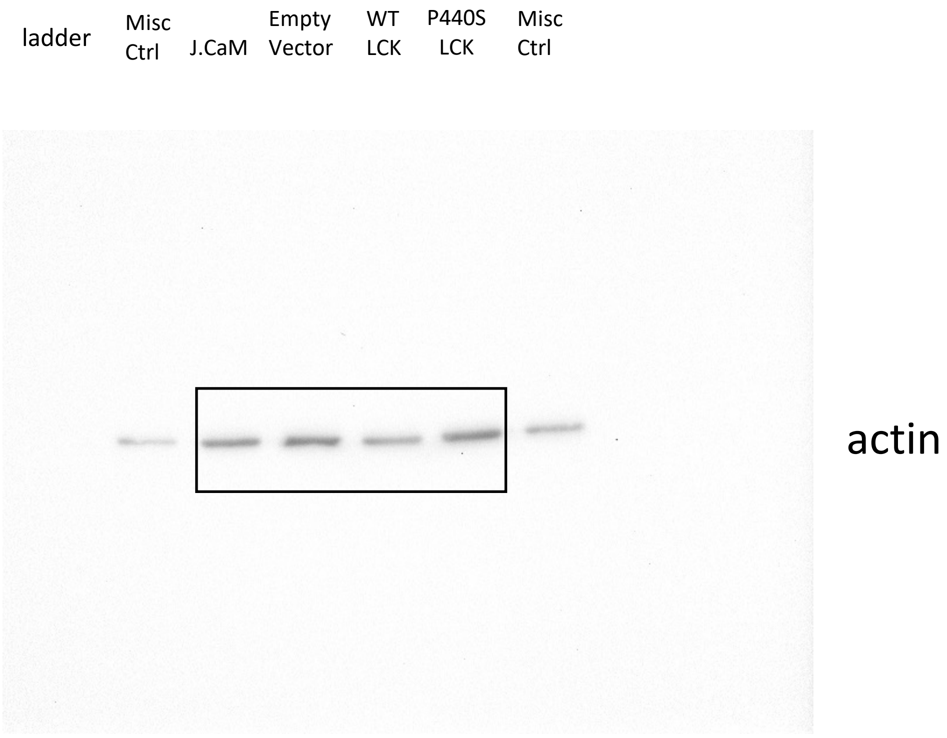
ladder
Misc Ctrl
J.CaM
Empty Vector
WT LCK
P440S LCK
Misc Ctrl
actin

Figure S2I

Colorimetric picture for ladder visualization

anti-pCD3ζ (Y142)
MW=17kD

70 kD
50 kD
40 kD
35 kD
25 kD
20 kD
15 kD

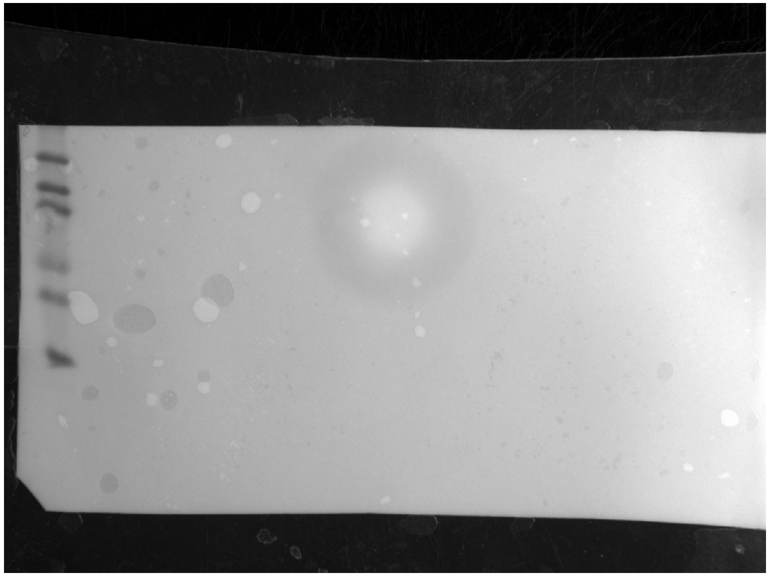

WT LCK

Stim (min): 0 0.5 1 2 5 20

Various Controls

70 kD
50 kD
40 kD
35 kD
25 kD
20 kD
15 kD

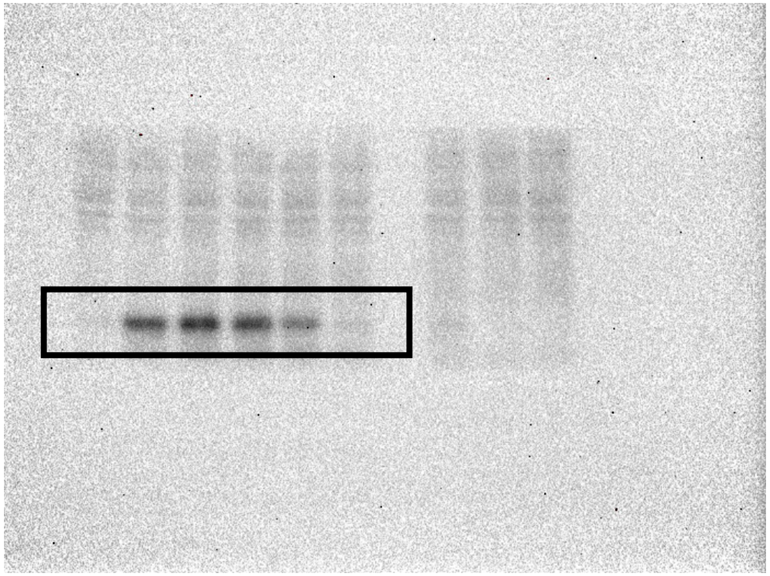

Empty Vector | P440S LCK | Control

Stim (min): 0 0.5 1 2 5 20 0 0.5 1 2 5 20

70 kD
50 kD
40 kD
35 kD
25 kD
20 kD
15 kD

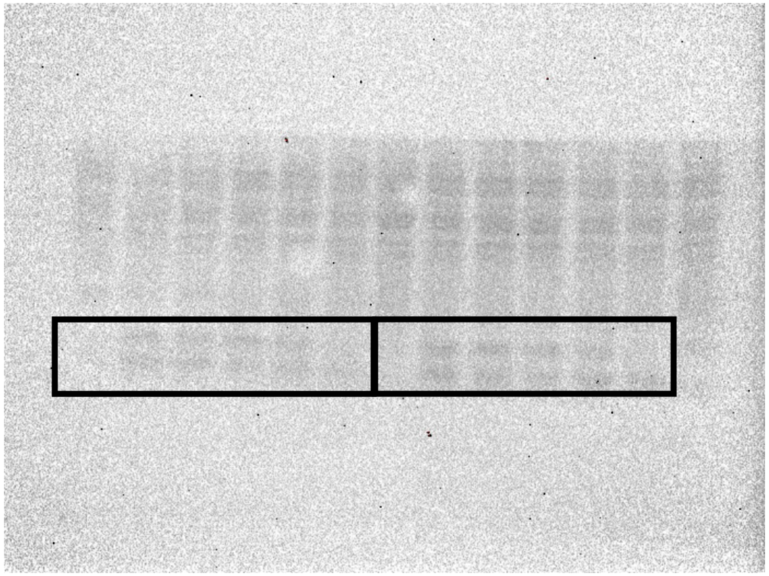

anti-pCD3ζ
MW=17kD

Colorimetric picture for ladder visualization

70 kD
50 kD
40 kD
35 kD
25 kD
20 kD
15 kD

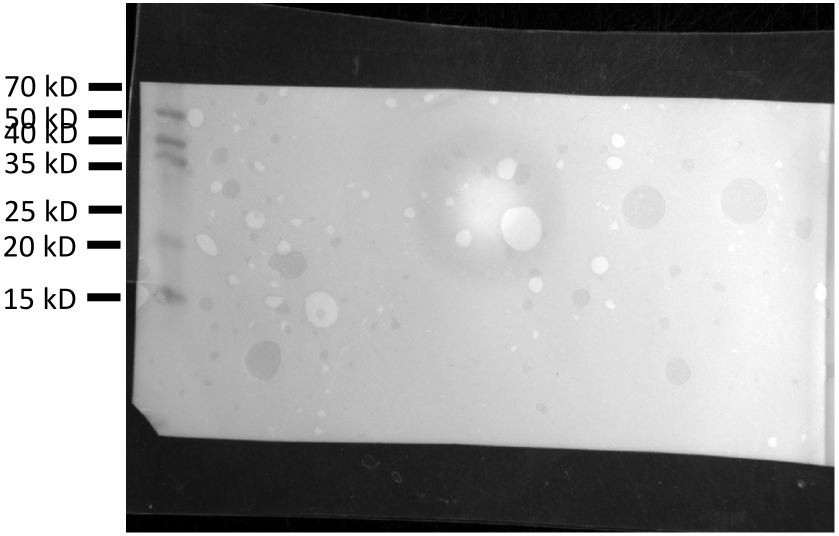

70 kD
50 kD
40 kD
35 kD
25 kD
20 kD
15 kD

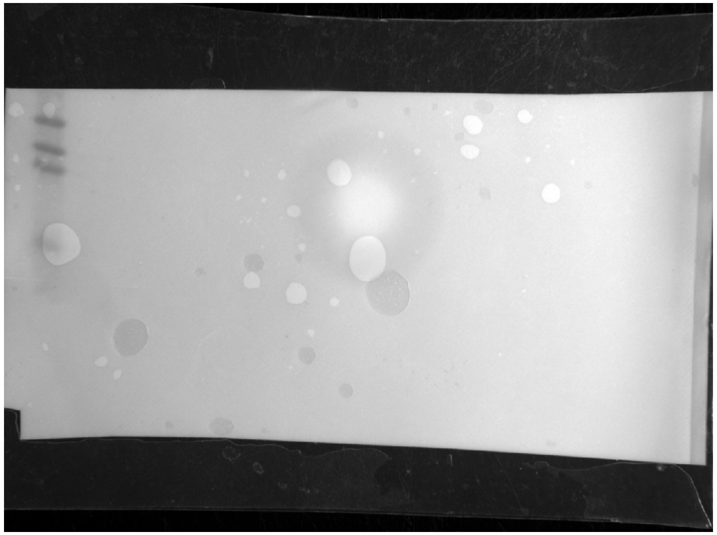

WT LCK

Stim (min): 0 0.5 1 2 5 20

Various Controls

70 kD
50 kD
40 kD
35 kD
25 kD
20 kD
15 kD

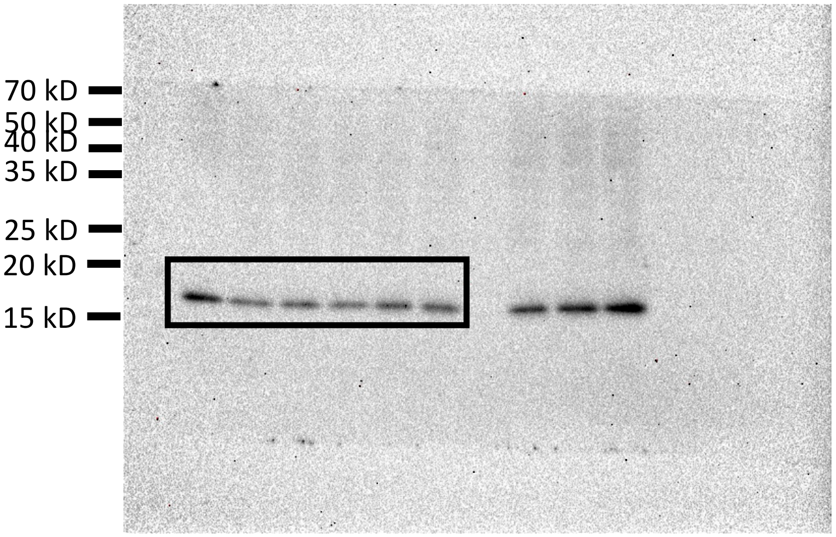

Empty Vector | P440S LCK | Control

Stim (min): 0 0.5 1 2 5 20 | 0 0.5 1 2 5 20

70 kD
50 kD
40 kD
35 kD
25 kD
20 kD
15 kD

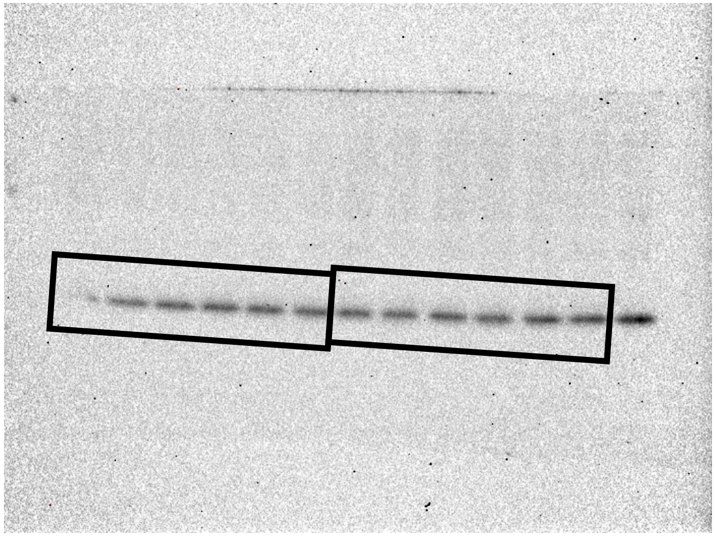

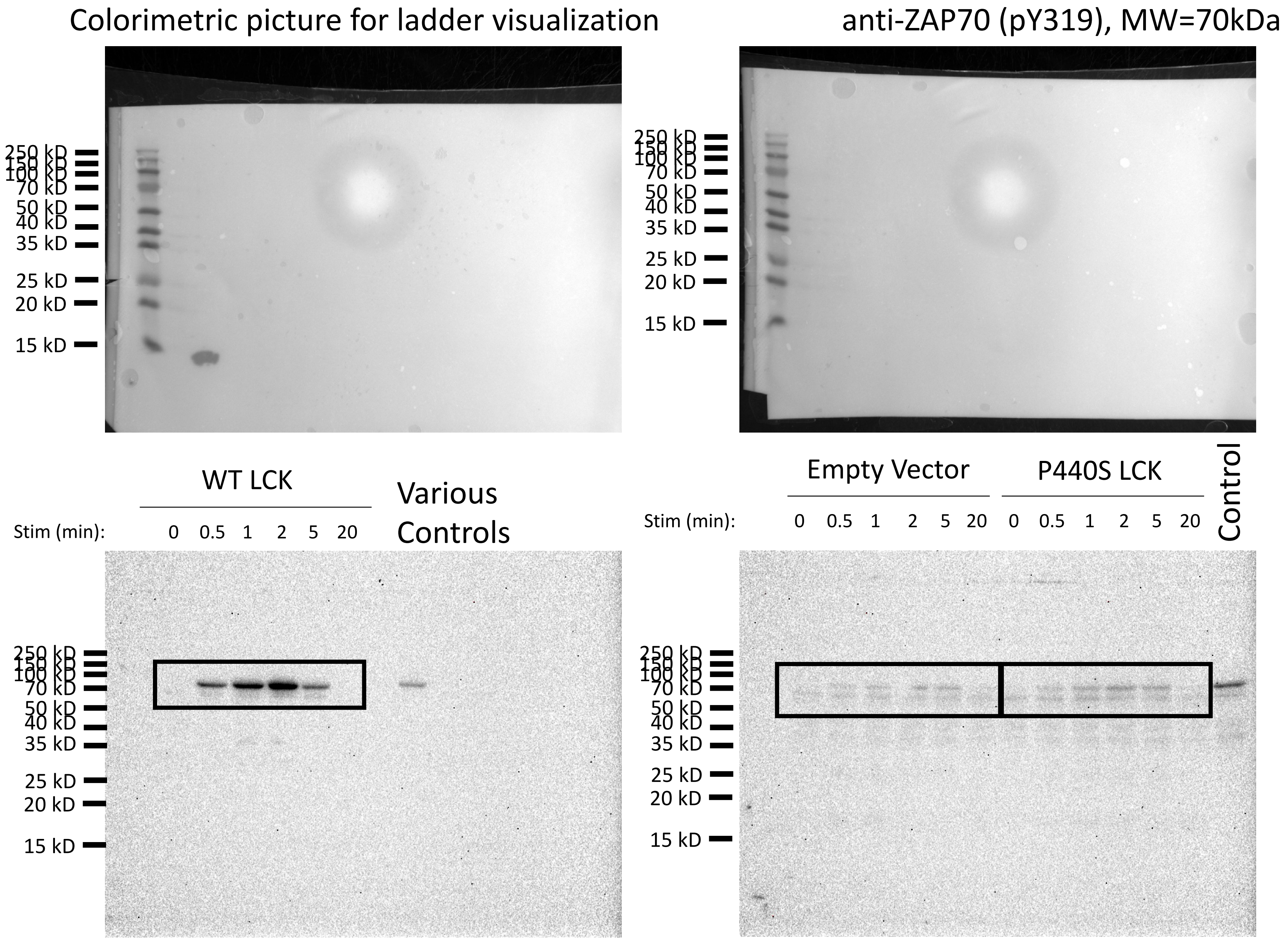

Colorimetric picture for ladder visualization
anti-ZAP70 (pY319), MW=70kDa
250 kD
150 kD
100 kD
70 kD
50 kD
40 kD
35 kD
25 kD
20 kD
15 kD
WT LCK
Various Controls
Stim (min):
0
0.5
1
2
5
20
Empty Vector
P440S LCK
Control

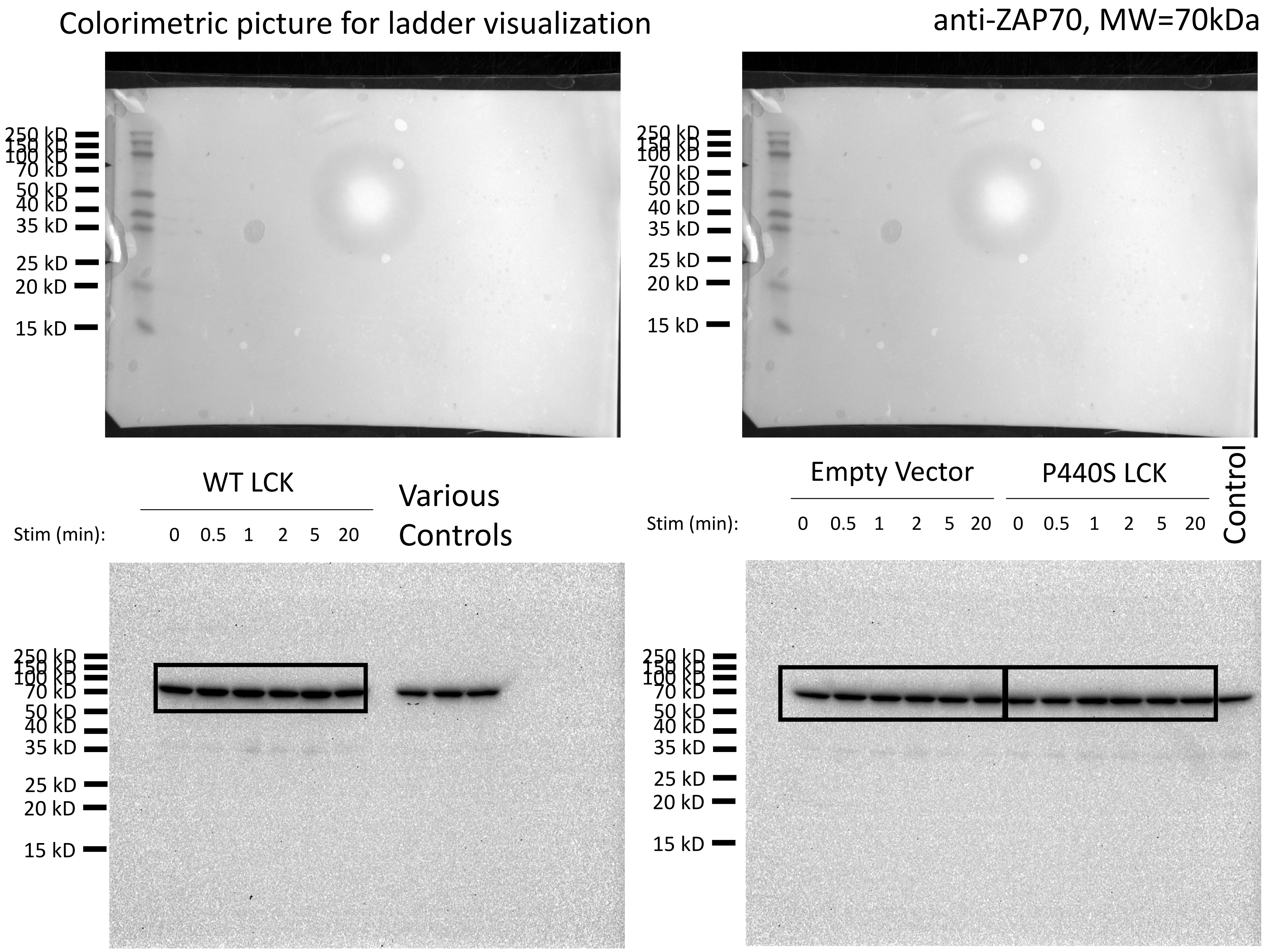
Colorimetric picture for ladder visualization
anti-ZAP70, MW=70kDa
250 kD
150 kD
100 kD
70 kD
50 kD
40 kD
35 kD
25 kD
20 kD
15 kD
WT LCK
Various Controls
Stim (min):
0 0.5 1 2 5 20
Empty Vector
P440S LCK
Control
Stim (min):
0 0.5 1 2 5 20 0 0.5 1 2 5 20

anti-PLCγ1 (pY783)
MW=155kDa

Colorimetric picture for ladder visualization

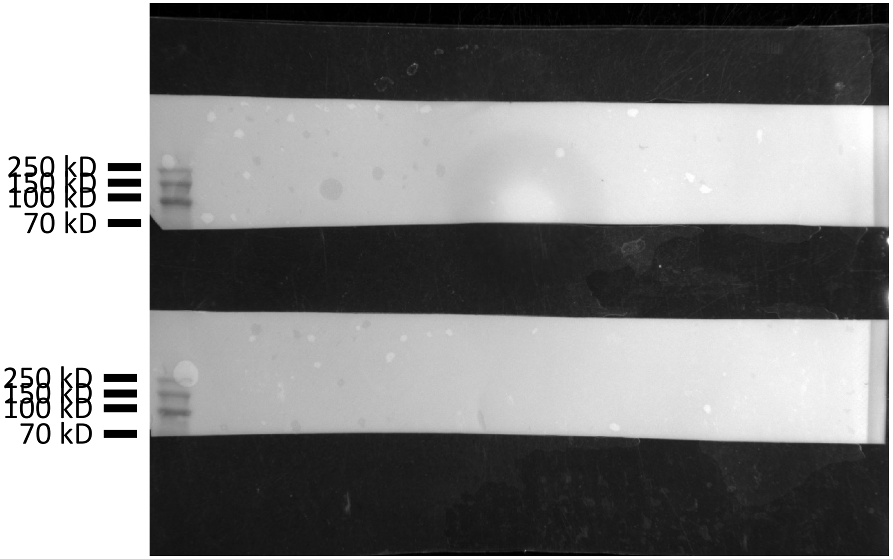

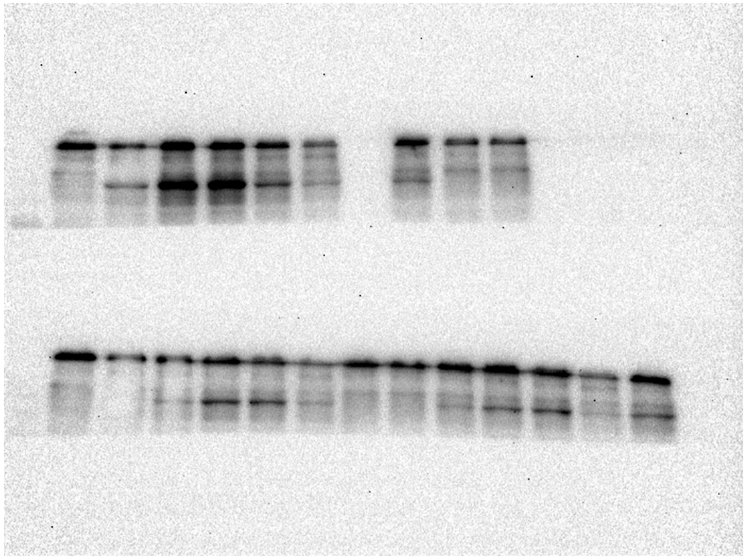

Without annotation

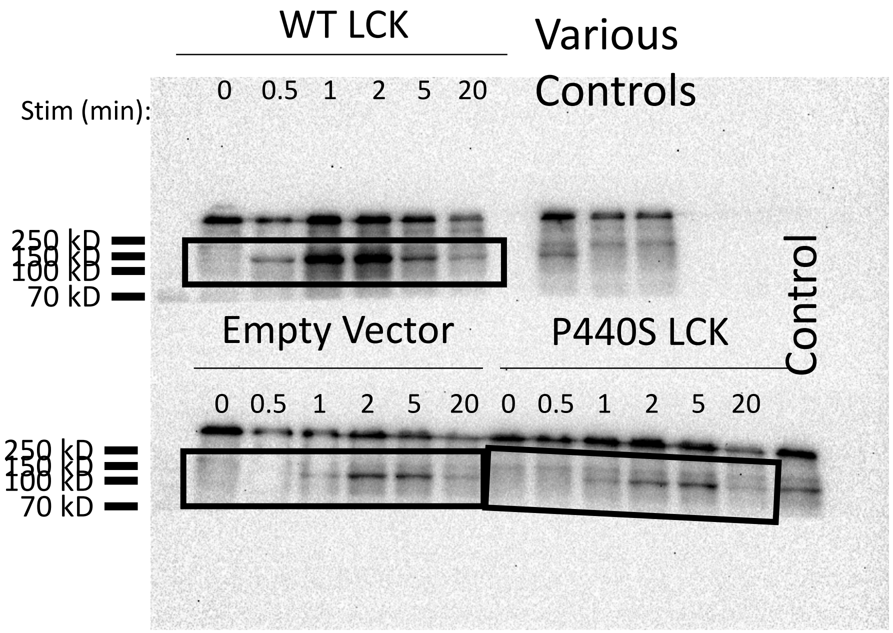

Annotated

Colorimetric picture for ladder visualization

anti-PLCγ1
MW=155kDa

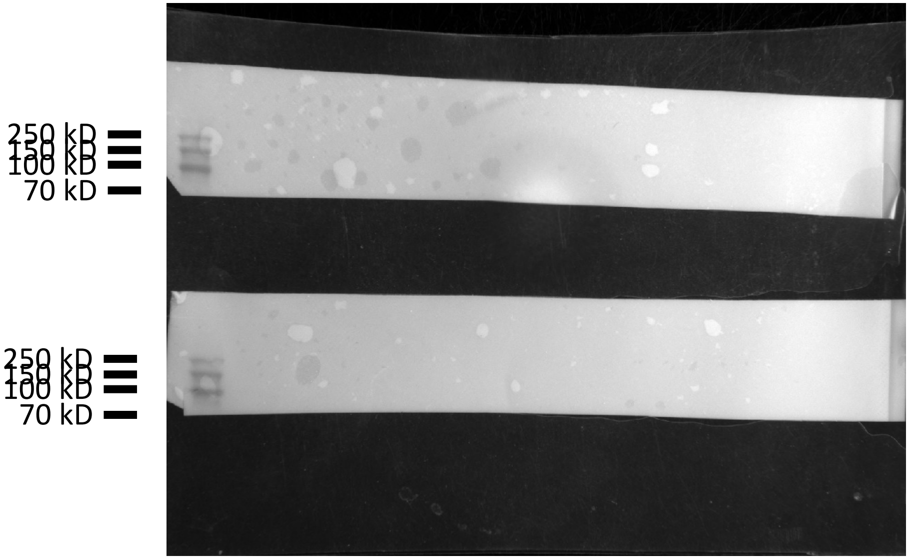

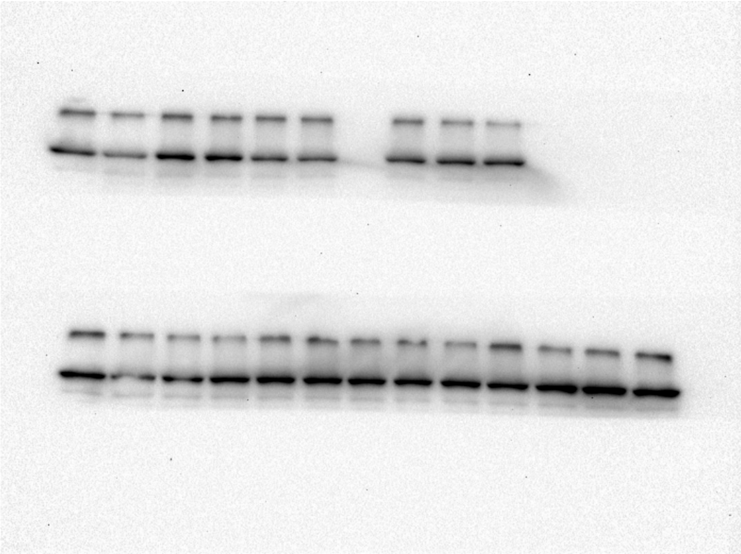

Without annotation

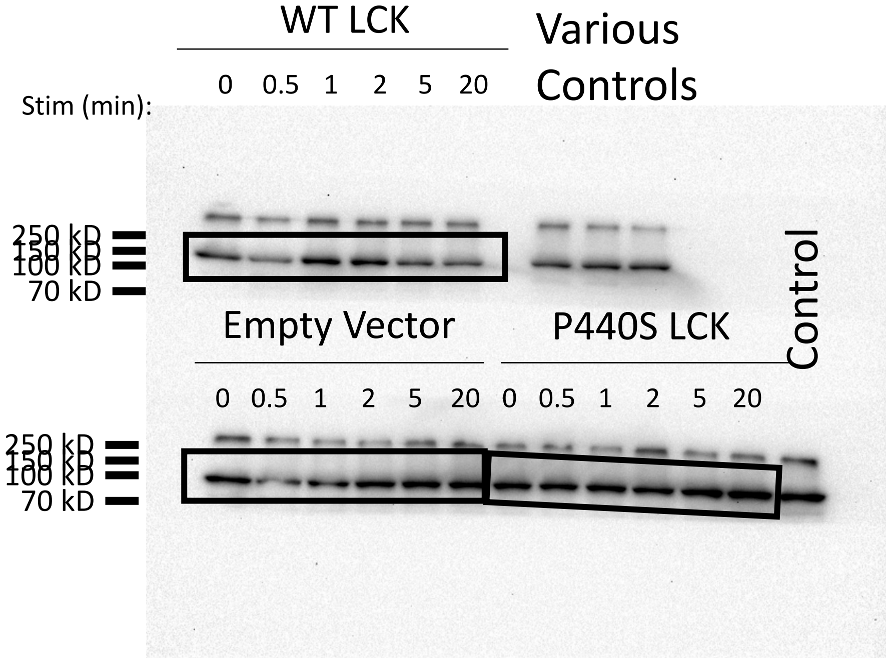

Annotated

Colorimetric picture for ladder visualization

anti-ERK (pT202/pT204)
MW=42kDa, 44kDa

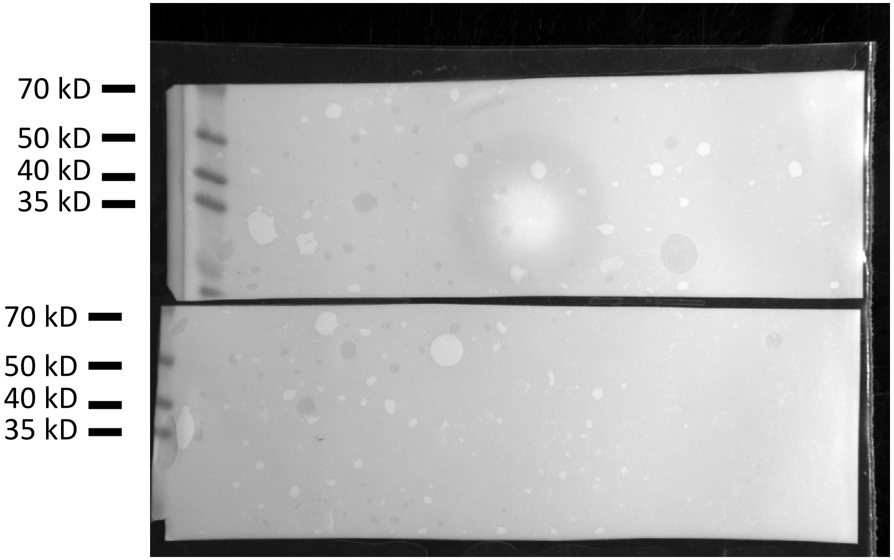

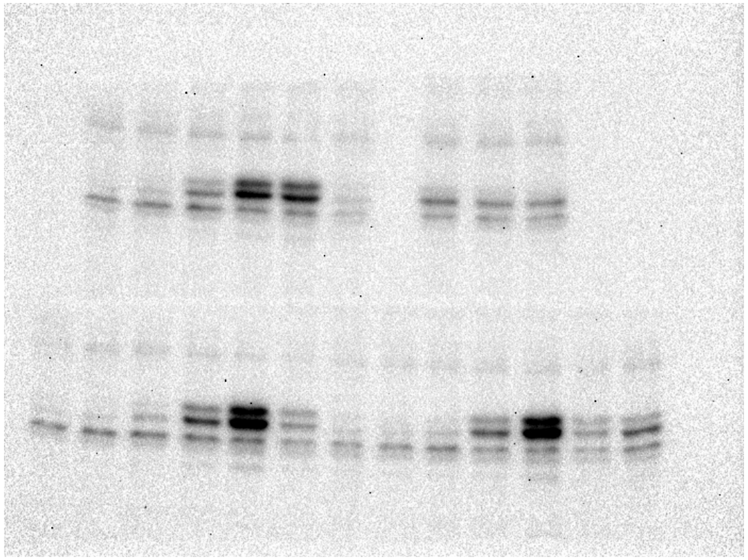

Without annotation

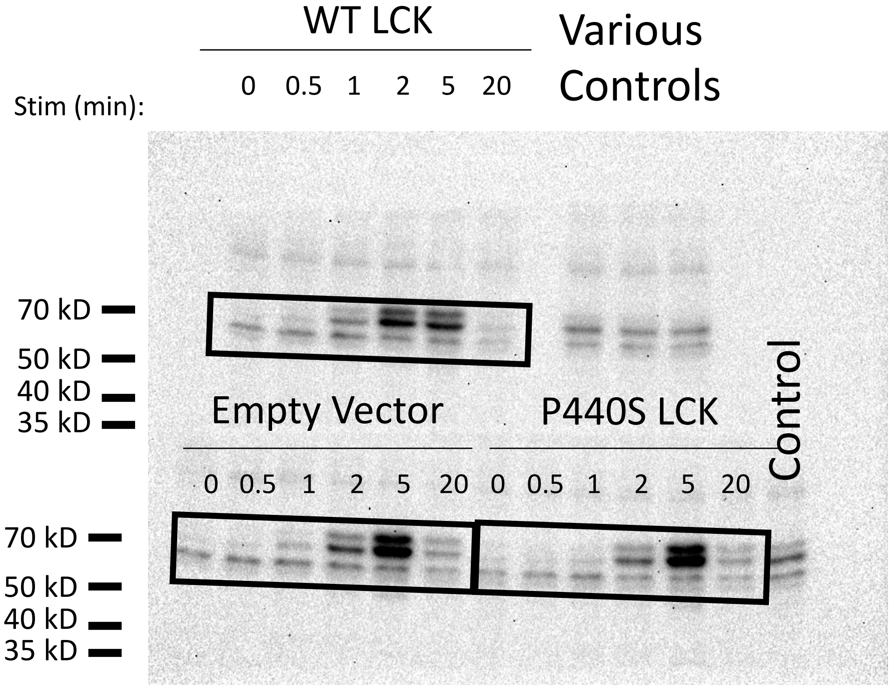

Annotated

Colorimetric picture for ladder visualization

anti-ERK
MW=42kDa, 44kDa

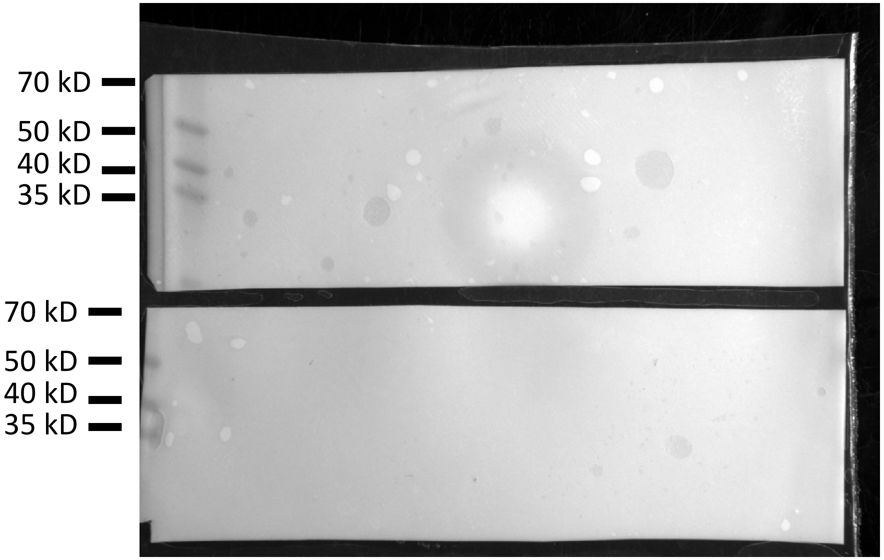

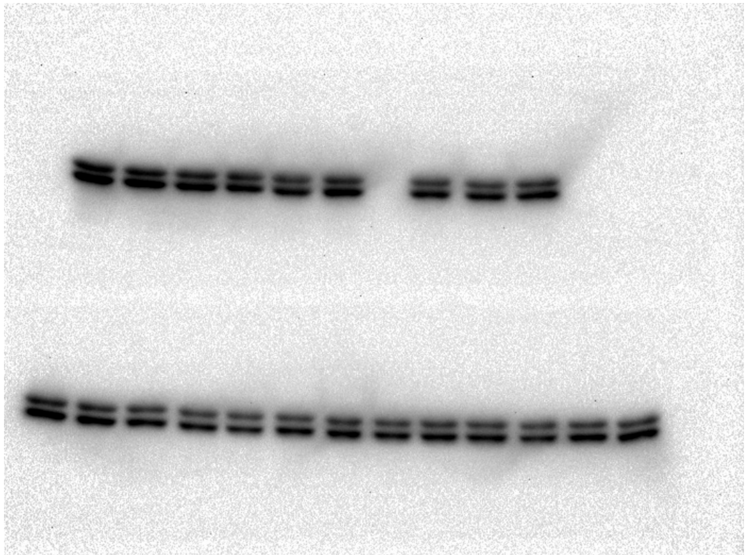

Without annotation

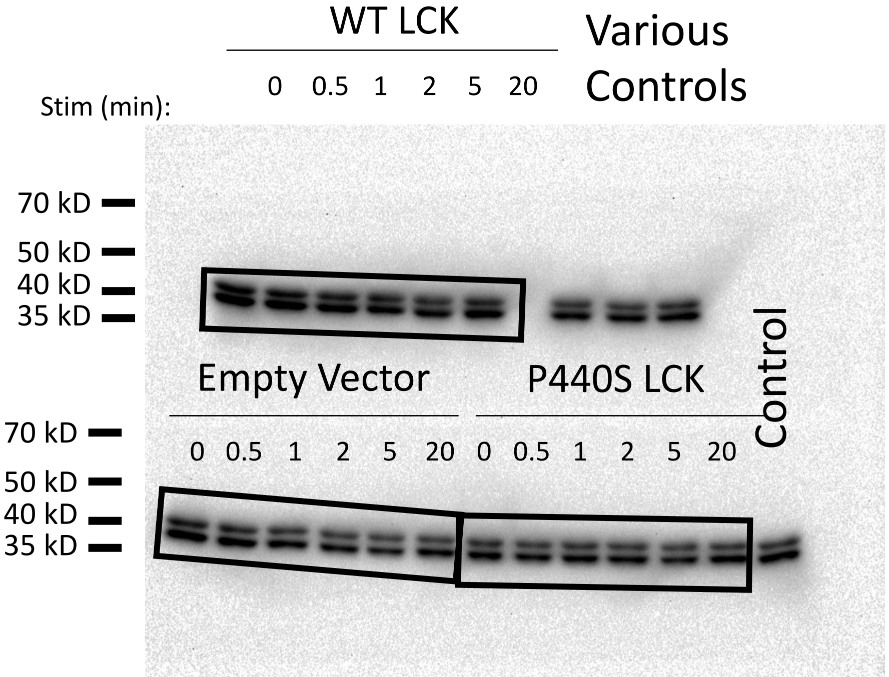

Annotated

Colorimetric picture for ladder visualization

actin

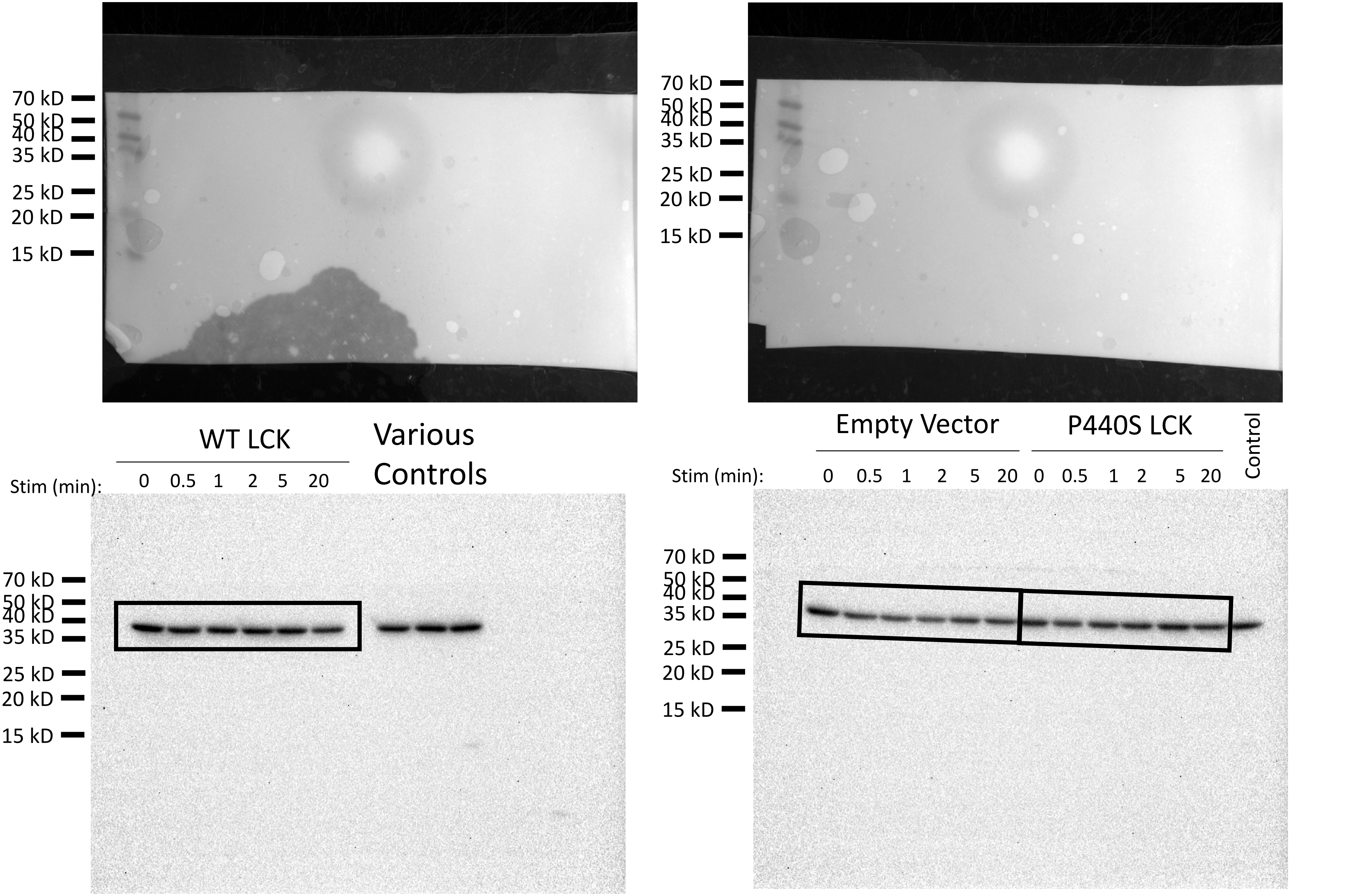

Supplement: SourceData FS2 — contains original blots for Fig. S2. [file JEM_20230927_SourceDataFS2.pdf]
