# Supplementary material for: A partial human LCK defect causes a T cell immunodeficiency with intestinal inflammation
Source: J Exp Med. 2023 Nov 14;221(1):e20230927. doi: 10.1084/jem.20230927 (PMC10644909; doi:10.1084/jem.20230927)
Supplement: SourceData FS3 — contains original blots for Fig. S3. [file JEM_20230927_SourceDataFS3.pdf]

Figure SF3A

Colorimetric picture for ladder visualization

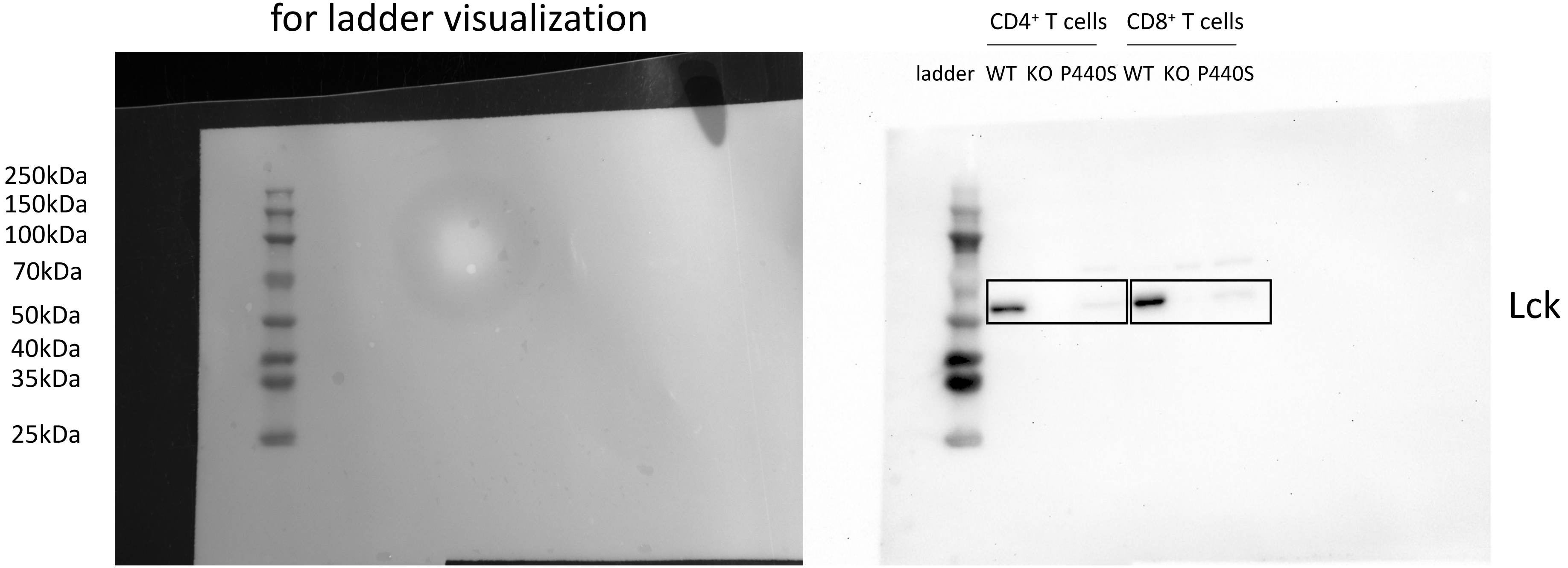

LCK MW=56kDa

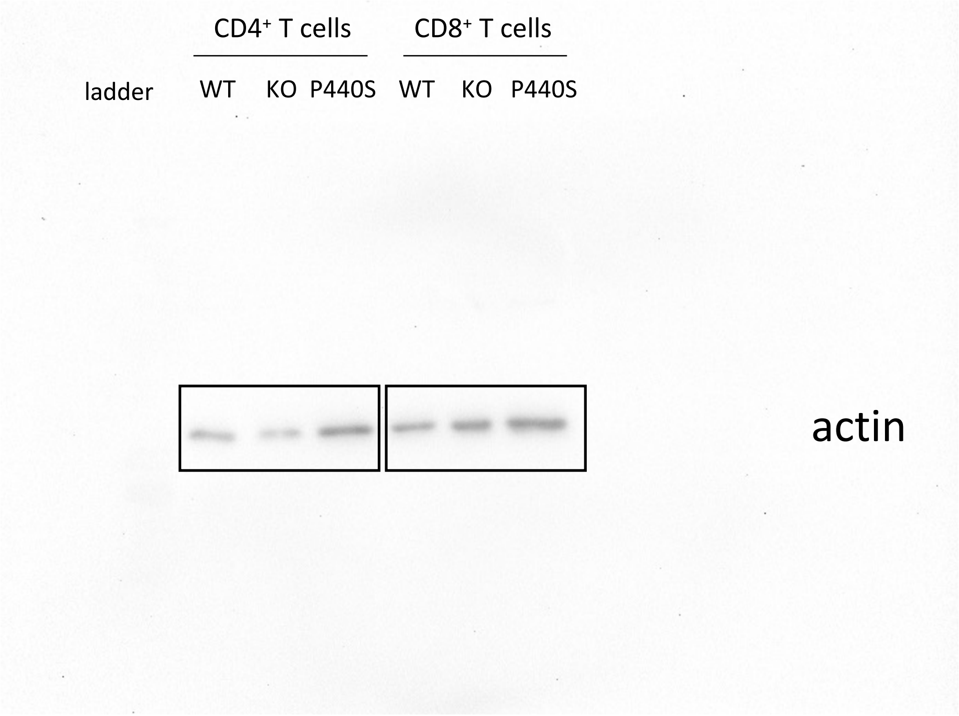
CD4+ T cells
CD8+ T cells
ladder
WT
KO
P440S
WT
KO
P440S
actin
